# An Open-Label, Randomised Study of Dihydroartemisinin-Piperaquine Versus Artesunate-Mefloquine for Falciparum Malaria in Asia

**DOI:** 10.1371/journal.pone.0011880

**Published:** 2010-07-30

**Authors:** Neena Valecha, Aung Pyae Phyo, Mayfong Mayxay, Paul N. Newton, Srivicha Krudsood, Sommay Keomany, Maniphone Khanthavong, Tiengkham Pongvongsa, Ronnatrai Ruangveerayuth, Chirapong Uthaisil, David Ubben, Stephan Duparc, Antonella Bacchieri, Marco Corsi, Bappanad H. K. Rao, Prabash C. Bhattacharya, Nagesh Dubhashi, Susanta K. Ghosh, Vas Dev, Ashwani Kumar, Sasithon Pukittayakamee

**Affiliations:** 1 National Institute of Malaria Research, Delhi, India; 2 Shoklo Malaria Research Unit, Tak, Thailand; 3 Wellcome Trust-Mahosot Hospital-Oxford University Tropical Medicine Research Collaboration, Mahosot Hospital, Vientiane, Lao People's Democratic Republic; 4 Faculty of Postgraduate Studies and Research, University of Health Sciences, Vientiane, Lao People's Democratic Republic; 5 Centre for Clinical Vaccinology and Tropical Medicine, Churchill Hospital, Oxford, United Kingdom; 6 Faculty of Tropical Medicine, Mahidol University, Bangkok, Thailand; 7 Salavan Provincial Hospital, Salavan, Lao People's Democratic Republic; 8 Centre of Malariology, Parasitology and Entomology, Vientiane, Lao People's Democratic Republic; 9 Savannakhet Provincial Malaria Station, Savannakhet, Lao People's Democratic Republic; 10 Mae Sod Hospital, Tak, Thailand; 11 Mae Ramat Hospital, Tak, Thailand; 12 Medicines for Malaria Venture, International Centre Cointrin, Geneva, Switzerland; 13 Sigma-Tau Industrie Farmaceutiche Riunite, Pomezia, Italy; 14 Wenlock District Government Hospital, Mangalore, India; 15 Down Town Hospital, Guwahati, India; 16 Department of Medicine, Goa Medical College and Hospital, Bambolim, India; 17 National Institute of Malaria Research Field Unit, Bangalore, India; 18 National Institute of Malaria Research Field Unit, Guwahati, India; 19 National Institute of Malaria Research Field Unit, Panaji, India; The George Washington University Medical Center, United States of America

## Abstract

**Background:**

The artemisinin-based combination treatment (ACT) of dihydroartemisinin (DHA) and piperaquine (PQP) is a promising novel anti-malarial drug effective against multi-drug resistant falciparum malaria. The aim of this study was to show non-inferiority of DHA/PQP vs. artesunate-mefloquine (AS+MQ) in Asia.

**Methods and Findings:**

This was an open-label, randomised, non-inferiority, 63-day follow-up study conducted in Thailand, Laos and India. Patients aged 3 months to 65 years with *Plasmodium falciparum* mono-infection or mixed infection were randomised with an allocation ratio of 2∶1 to a fixed-dose DHA/PQP combination tablet (adults: 40 mg/160 mg; children: 20 mg/320 mg; n = 769) or loose combination of AS+MQ (AS: 50 mg, MQ: 250 mg; n = 381). The cumulative doses of study treatment over the 3 days were of about 6.75 mg/kg of DHA and 54 mg/kg of PQP and about 12 mg/kg of AS and 25 mg/kg of MQ. Doses were rounded up to the nearest half tablet. The primary endpoint was day-63 polymerase chain reaction (PCR) genotype-corrected cure rate. Results were 87.9% for DHA/PQP and 86.6% for AS+MQ in the intention-to-treat (ITT; 97.5% one-sided confidence interval, CI: >−2.87%), and 98.7% and 97.0%, respectively, in the per protocol population (97.5% CI: >−0.39%). No country effect was observed. Kaplan-Meier estimates of proportions of patients with new infections on day 63 (secondary endpoint) were significantly lower for DHA/PQP than AS+MQ: 22.7% versus 30.3% (p = 0.0042; ITT). Overall gametocyte prevalence (days 7 to 63; secondary endpoint), measured as person-gametocyte-weeks, was significantly higher for DHA/PQP than AS+MQ (10.15% versus 4.88%; p = 0.003; ITT). Fifteen serious adverse events were reported, 12 (1.6%) in DHA/PQP and three (0.8%) in AS+MQ, among which six (0.8%) were considered related to DHA/PQP and three (0.8%) to AS+MQ.

**Conclusions:**

DHA/PQP was a highly efficacious drug for *P. falciparum* malaria in areas where multidrug parasites are prevalent. The DHA/PQP combination can play an important role in the first-line treatment of uncomplicated falciparum malaria.

**Trial Registration:**

Controlled-Trials.com ISRCTN81306618

## Introduction

The bisquinoline piperaquine (PQP) was first synthesised in the 1960s independently by teams in China and France. In 1978, because of its greater potency and tolerability relative to chloroquine, PQP replaced chloroquine in the Chinese National Malaria Control Programme. Such was the success of the programme, that the following decade saw the use of PQP decrease because of the decrease in patients suffering from malaria.

In 1990, Chinese scientists working to develop alternative anti-malarial treatments that might offer higher cure rates with better tolerability profiles included PQP phosphate in an artemisinin-based combination treatment (ACT), known as China-Vietnam 4 (CV4®), that also included dihydroartemisinin (DHA), trimethoprim and primaquine phosphate [1], [2]. During the development programme [3], doses of each component of the combination were modified resulting in a new formulation, CV8®, which was tested in Vietnam [4], [5] and administered in the Vietnamese Malaria Control Programme in 2000. This programme was widely successful despite the presence of chloroquine resistance in Vietnam [6]. However, concerns about the association of red cell haemolysis with primaquine in populations with glucose-6-phosphate dehydrogenase deficiency [7] and the questionable anti-malarial potency of trimethoprim led to the removal of these two components of the drug combination in CV8®. The remaining two components of the regimen, the artemisinin DHA and PQP (Artekin®), provide a combination that is relatively inexpensive and has been shown to be effective both in curing malaria and preventing re-infection [2], [8]–[12].

Since their introduction in the 1990s, ACTs have been found to be highly effective treatments for malaria [13]. Across Asia, Africa and South America, clinical and parasitological responses to the combination of DHA and PQP have generally exceeded the 95% value that the World Health Organization (WHO) recommend for anti-malarial treatments [14]. However, in recent years there have been suggestions that the overall efficacy of ACTs in Thailand and Cambodia may be declining. Evidence of falling efficacy has been characterised by reductions in the proportions of patients clearing their parasitaemia by day 2 of treatment in the Thailand-Myanmar border region [15], [16], and by reductions in polymerase chain reaction (PCR)-corrected cure rates at 28- and 42-day follow-up assessments in the Cambodia-Thailand border region [17]. Relatively low cure rates were also reported from Papua New Guinea [18] and, most recently, there has been evidence of reduced artesunate susceptibility in Western Cambodia [19], [20]. Historically, anti-malarial drug resistance has spread westwards from Cambodia through South Asia to Africa [21]. Consequently, the recent reports of potential resistance to artemisinins alone and ACTs are of great concern.

In order to assess the safety and efficacy of the treatment combination of DHA and PQP in Asia, we conducted a randomised trial in Thailand, Laos and India comparing DHA/PQP with another ACT, artesunate (AS) plus mefloquine (MQ). All study sites were located in areas of notable chloroquine resistance. Dihydroartemisinin plus PQP was administered as a single tablet (DHA/PQP) and AS plus MQ were administered as separate loose tablets (AS+MQ). This study had the largest sample size to date of any study assessing DHA/PQP in South East Asia.

## Methods

The protocol and amendments for this trial and supporting CONSORT checklist are available as supporting information; see Protocol S1 to S8 and Checklist S1.

### Study regions

This study was conducted in six centres in Thailand (Hospital for Tropical Diseases, Faculty of Tropical Medicine, Mahidol University, Bangkok; Suanphung Hospital, Ratchaburi; Proppra Hospital, Proppra District, Tak; Shoklo Malaria Research Unit, Mae Sod District, Tak; Mae Sod Hospital, Muang District, Tak; and Mae Ramat Hospital, Mae Ramat District, Tak), two centres in Laos (Phalanxay District Hospital, Savannakhet Province; Xepon District Hospital, Savannakhet Province) and three centres in India (Down Town Hospital, Guwahati, Assam; Goa Medical College and Hospital, Goa; and Wenlock District Government Hospital, Mangalore) over two malaria seasons, with patients screened from June 2005 to February 2007.

The study regions provided a range of transmission conditions and standard treatments. Study regions in Thailand were located in malarious forest on the Thai-Myanmar border in regions of unstable, low and seasonal malaria transmission that used AS+MQ as a first-line treatment. Nearly all infections with *Plasmodium falciparum* and *P. vivax* in the region are symptomatic and *P. falciparum* is multi-drug resistant, with high levels of resistance to chloroquine [22], [23]. Malaria transmission in the Phalanxay and Xepon districts in Laos was seasonal with a peak just after the heavy rainy months of July to August. Previous studies in the Phalanxay district showed high levels of chloroquine resistance [24], [25]. The first-line treatment was artemether/lumefantrine. In India, study regions included areas of perennial transmission (Assam), perennial transmission with a seasonal peak from June to September (Goa), and transmission active in post-monsoon months (Mangalore). Chloroquine resistance was present at all study sites, with site records showing 28-day treatment failure rates of 32% in Assam (in 2002), 54% in Goa (in 2007) and 67% in Mangalore City (in 2005). Artemisinin-based combination treatments are used as standard treatments in Goa, Mangalore and Assam (specifically AS plus sulphadoxine/pyrimethamine).

### Patients

Males and females aged from 3 months to 65 years weighing at least 5 kg with fever or history of fever (≥37.5°C) and microscopically confirmed mono-infection with *P. falciparum* (asexual forms parasitaemia ≥80 per µL ≤200,000 per µL) or mixed infection were eligible for the study. Local regulations were followed in India that required all recruited patients to be aged 18 years and over. Key exclusion criteria were: severe malaria, treatment with MQ in the 60 days prior to screening, treatment with DHA/PQP in the 3 months prior to screening and >4% parasitised red blood cells. Pregnant or lactating women were not eligible for the study.

### Study design

This was a randomised, Phase III, open-label study with two treatment arms: DHA/PQP and the active comparator AS+MQ. At the time the study was designed, there was no single first line treatment in the countries involved in the study. However, AS+MQ was first line treatment in Thailand, was well characterized and was widely used in all the considered countries. This made AS+MQ suitable as the control treatment against which the efficacy and safety of DHA/PQP would be tested. As the study was not blinded, to limit bias, the following procedures were put in place: 1) Randomisation was conducted under blinded conditions: the blind to the investigator and patient in the randomisation process was maintained by the use of sealed envelopes. 2) Evaluation of the PCR test results was blinded (centralised at the Prince Leopold Institute of Tropical Medicine, Antwerp, Belgium, with quality control at Shoklo Malaria Research Unit, Mae Sod, Thailand). 3) The chairman and the statistician of the independent Data Monitoring Committee reviewed the most relevant safety data and participated in the final blinded data review meeting where all decisions about assessment of the primary outcome and patient allocation to the pre-defined populations were made under blinded conditions. The randomisation list was generated by an external contract research organisation (MDS Pharma Services) using the plan procedure of SAS (Cary, NC, USA). Patients were allocated to receive either DHA/PQP or AS+MQ following a 2∶1 randomisation schedule ratio. The unbalanced ratio was chosen to increase the chance of detecting rare adverse reactions and to provide more precise estimates of cure rates in the DHA/PQP arm.

Dihydroartemisinin/PQP (Eurartesim™, Sigma-Tau, Italy) was given once daily, on days 0, 1 and 2 of the study, at the standard dosage of 2.25 mg/kg and 18 mg/kg per dose of DHA and PQP, respectively, rounded up to the nearest half tablet. To facilitate the correct dosing of DHA/PQP, two formulations were used (DHA 20 mg + PQP 160 mg and DHA 40 mg + PQP 320 mg). Over the 3 days, the cumulative doses were of about 6.75 mg/kg of DHA and 54 mg/kg of PQP. Artesunate and MQ (Mepha Ltd, Switzerland) were administered as separate tablets containing AS 50 mg and MQ 250 mg; AS was administered at 24-h intervals on days 0, 1 and 2 with a daily dose of 4 mg/kg and MQ was administered at 15 mg/kg on day 1 and 10 mg/kg on day 2 at 24-h intervals, but was not administered on day 0.

As part of their routine treatment, febrile patients who attended the study centres underwent a thick blood smear test for malaria before any anti-malarial treatment was administered. If the smear was found to be positive for *P. falciparum*, patients were told about the study and offered the opportunity to participate. Eligible patients who agreed to participate were given detailed explanations of the trial from study staff, including the information that they would be given one of two different anti-malaria treatments on a random basis. Participating patients all provided written informed consent. Patients who declined to participate were provided with treatment for uncomplicated *P. falciparum* malaria, standard for the area in which they lived. Participating patients were managed as outpatients and doses were given under medical supervision, although at some centres patients could be hospitalised according to local practice, which ranged from 3 days at Mae Ramat Hospital, Thailand and at centres in Laos and India to 28 days at Mahidol University, Thailand. If not in hospital, patients were encouraged to return to the study centre for assessments on days 1, 2, 3, 7, 14, 21, 28, 35, 42, 49, 56 and 63, and they were also told that they could make unscheduled visits on any day on which they felt unwell. Patients were followed-up for 63 days.

In accordance with WHO (WHO 2003 and 2009) and standard practice in malaria clinical trials, the primary endpoint of the study was the PCR-corrected cure rate, based on the adequate clinical and parasitological response (ACPR), which was defined as the absence of parasitaemia irrespective of the patient's body temperature, with the patient not meeting any of the pre-defined criteria of early treatment failure or late clinical or parasitological failure (see below). In more detail, the primary endpoint was defined using two methods. The first method was based purely on the standard definitions of early/late clinical and parasitological failure as defined by the WHO [26]. This endpoint is referred to as the true treatment failure and is defined as the sum of early treatment failures and late recrudescences, which included late treatment failures that were assessed as recrudescences according to PCR analysis (100 minus the true treatment failure rate provides the WHO cure rate). The second method, agreed with the Data Monitoring and the Clinical Development Committees, was based on a pre-defined procedure that expanded the WHO definitions with a set of rules allowing the evaluation of each individually randomised patient (for definitions see Table 1). This approach was considered to be primary because it was deemed to be in line with the requirements of the most stringent regulatory authorities. All cases not strictly matching the WHO definitions and/or the described procedure were reviewed individually at the final blinded data review meeting. Day 63 was chosen as the primary time-point because of the long half lives of piperaquine and mefloquine and because the risk of new infection is lower than in other parts of the world.

**Table 1:** Rules used to determine patient outcome for the ITT and per protocol populations for the day 63 uncorrected adequate clinical and parasitological response (ACPR) and polymerase chain reaction (PCR)-corrected ACPR.

Step	Event to be assessed	ITT	Per Protocol
**Day-63 uncorrected adequate clinical and parasitological response**			
1	Informative withdrawal before or at day 63: any reason except lost to follow-up	Failure	Failure or excluded depending on reason
2	Non-informative withdrawal before or at day 63: lost to follow-up	Failure	Excluded
3^1^	Presence of major protocol violation	No effect	Excluded
4	ETF^2^, LCF^3^, and LPF^4^ in accordance with the WHO criteria	Failure	Failure
5^1^	Data collected on CRF (such as adverse events) raising the suspicion of recurrence of malaria	Failure	Failure
6^1^	Presence of missing parasitaemia at two or more consecutive scheduled visits or presence of an isolated missing parasitaemia not preceded and followed by a negative parasitaemia	Failure	Failure
7^1^	Administration of drugs with a known or suspected anti-malaria action as rescue treatment	Failure	Failure
8^1^	Administration of drugs with a known or suspected anti-malaria action as non rescue treatment	Failure	Excluded
**PCR-corrected adequate clinical and parasitological response**			
9	PCR: non interpretable or missing or not done at or after day 4	Failure	Excluded
10	PCR = new infection or uncorrected ACPR = failure for non-falciparum Plasmodia	Success	Success
11	PCR = recrudescence	Failure	Failure

ITT  =  intention to treat.

1 Cases in these categories were individually revised at the blind data review meeting. Protocol violations were pre-defined.

2 ETF  =  early treatment failure, defined as development of danger signs (recent convulsions, altered consciousness, lethargy, unable to drink or breast feed, recurrent vomiting, unable to stand/sit due to weakness) or severe malaria (unarousable coma, repeated convulsions, severe anaemia, respiratory distress, jaundice) on days 0, 1, 2 or 3, and the presence of parasitaemia; parasitaemia with a parasite count on day 2 greater than that on day 0 irrespective of body temperature; parasitaemia on day 3 with fever (temperature ≥37.5°C); or parasitaemia on day 3≥25% of count on day 0.

3 LCF  =  late clinical failure, defined as development of danger signs or severe malaria after day 3 in the presence of parasitaemia or presence of parasitaemia and temperature ≥37.5°C (or history of fever) on any day from day 4 to day 63, without previously meeting the criteria of early treatment failure.

4 LPF  =  late parasitological failure, defined as reappearance of parasitaemia after initial clearance between day 7 and day 63 and temperature <37.5°C, without previously meeting the criteria of early treatment failure or late clinical failure.

Secondary endpoints included PCR-corrected adequate clinical and parasitological response on days 28 and 42, PCR-uncorrected adequate clinical and parasitological response (PCR-uncorrected), proportion of patients with early and late treatment failure, proportion of aparasitaemic patients, proportion of afebrile patients, number of new infections, gametocyte carriage and the safety profile of the two treatments including adverse events and 12-lead electrocardiograph parameters (QT interval corrected according to the methods of Bazett, QTc_(B)_, and Fridericia, QTc_(F)_).

### Procedures

The presence of *P. falciparum* was verified at all study visits including screening, using thick and thin Giemsa-stained blood smears obtained from the patient to calculate parasite density, which was initially calculated by counting the number of asexual parasites per 500 leukocytes in the thick blood film, based on an assumed white cell count of 8,000 cells per µL. Blood smears were obtained from a finger prick applied directly to a microscope slide to create the blood film. Parasite density per µL was calculated as: (number of parasites counted ×8,000)/(number of leukocytes counted). For samples with higher levels of parasitaemia (>3 parasites/1000 red blood cells), parasite density was calculated from the thin film per 1000 red blood cells as: (number of *P. falciparum* trophozoites per 1000 red blood cells x haematocrit ×125.6). Gametocyte prevalence was also evaluated. Both the thick and thin blood smear readings were done locally following the above described procedure, while the gametocyte assessments were carried out in accordance with standard practice at each individual site. All technicians who read the slides had undergone appropriate training in malaria-related microscopy and had at least 5 years experience in reading blood smears. A process of quality control was used to monitor the values being provided by the local laboratories. One in five of every samples was sent to a central, independent laboratory (Department of Parasitology and Medical Entomology, Muhimbili University of Health and Allied Sciences, Dar Es Salaam, Tanzania) for review.

Three spots of blood were collected on 3MM filter paper (Whatman, UK) at the enrolment visit and at any visit after day 7 for PCR analysis. Filter papers were dried and individually stored in a plastic bag containing silica gel. All filter papers were subsequently transferred to the Institute of Tropical Medicine (Antwerp, Belgium) where centralised genotyping was conducted; deoxyribonucleic acid was purified as described elsewhere [27]. Three polymorphic genetic markers, MSP1, MSP2 and GluRP were used to distinguish recrudescence from new infections [28], [29]. Recrudescence was defined as at least one identical allele for each of the three markers in the pre-treatment and post-treatment samples. New infections were diagnosed when all alleles for at least one of the markers differed between the two samples. An independent expert read all gels under blinded conditions (National Museum of Natural History, Paris, France). For quality control, 20% of the filter papers were re-analysed and read by an independent laboratory (Shoklo Malaria Research Unit, Mae Sot District, Tak, Thailand).

Twelve-lead electrocardiograms were recorded at days 0, 2, 7, 28 (if abnormal on day 7), 63 and on the day of any recurrent parasitaemia that occurred using the CarTouch device with CarTouch version 1.4.1 software (Cardionics SA, Brussels, Belgium). The electrocardiogram was viewed during recording and then transmitted via modem to MDS Pharma Services Central Telemedicine Department (Paris, France) for interpretation and reporting. The data were analysed using both Bazett's (QTc_(B)_) and Fridericia's (QTc_(F)_) correction methods. Electrocardiogram results were tabulated as normal, borderline or prolonged according to gender and age normal ranges (adult males and children (1–12 years): normal: <430 msec; borderline: 430–450 msec; prolonged: >450 msec. Adult females: normal: <450 msec; borderline: 450–470 msec; prolonged: >470 msec).

Blood samples were taken for standard laboratory assessments of haematology, biochemistry and urinalysis on days 0, 28, 63 (if abnormal on day 28) and on the day of any recurrent parasitaemia.

### Ethical approval and informed consent

Study staff conducted all interviews with patients, and children's parents, in their native language and they explained their rights under International Conference on Harmonisation-Good Clinical Practice (ICH-GCP). When obtaining informed consent from patients the signature of a witness was obtained if patients were unable to write. Consideration was given to the ethical implications of the randomisation ratio assigning more patients to receive DHA/PQP than AS+MQ during the design of the study. As the safety and tolerability profile of DHA and PQP has been well characterised at the doses proposed for the present study, it was considered acceptable to adopt this approach in the present study that would result in more patients receiving DHA/PQP.

The study protocol was approved by the following ethics committees: Institutional Ethics Committee, National Institute of Malaria Research (ICMR), Delhi, India; Goa Medical College and Hospitals Local Ethics Committee, Goa, India; Institutional Ethics Committee, Kasturba Medical College, Mangalore, India; Laos National Ethics Committee for Health Research (NECHR), National Institute of Public Health, Vientiane, Laos; Oxford Tropical Research Ethics Committee (OXTREC), University of Oxford, Headington, United Kingdom; The Ethical Review Committee for Research in Human Subjects, Ministry of Public Health, Nontaburi, Thailand; Tropical Medicine Ethics Committee (TMEC), Mahidol University, Rachathewi District, Bangkok, Thailand. The Food and Drug Department, Government of Laos PDR, approved the use of DHA/PQP in that country. The trial was conducted under the provisions of the Declaration of Helsinki (1964 and its subsequent amendments up to 2002) and in accordance with ICH-GCP. A Study Steering Committee, a Data Monitoring Committee and a Clinical Development Committee were created prior to the beginning of the trial, and worked independently to harmonise and monitor the study. The trial was registered prior to the enrolment of the first patient in the International Standard Randomised Controlled Trials Register, number ISRCTN81306618, at http://www.controlled-trials.com/isrctn/trial/l/0/81306618.html.

### Statistical methods

The statistical analysis was conducted according to a pre-defined data analysis plan. Three populations were prospectively planned. The intention-to-treat (ITT) population was defined as all randomised patients who took at least one dose of study treatment. The per protocol population was defined as all randomised patients who were eligible according to the main (pre-defined) protocol inclusion and exclusion criteria, received at least 80% of the study medication, underwent the day 63 assessment, took no other anti-malarial drugs (excluding rescue therapies) and, in the presence of asexual parasite stages on thick or thin blood smears, had an evaluable PCR test. Patients who missed visits from day 0 to day 2 were excluded from the per protocol population. The third population, referred to as modified ITT, was midway between the two described populations. Although this was pre-defined as the co-primary population (together with the per protocol), results in this paper are presented only for the two most extreme populations, i.e., ITT and per protocol populations, because these are standard and the findings were very similar in all populations analysed.

The analysis of the PCR-corrected and uncorrected ACPR (as defined in Table 1) was based on simple proportions and 97.5% (one-sided) confidence intervals (CIs) computed on the difference between these proportions of the test and reference treatments. If the lower limit of this CI was greater than −5% DHA/PQP could be considered non-inferior to AS+MQ. The primary time point was day 63 but this analysis was repeated also at days 42 and 28. Various sensitivity analyses were carried-out on the primary endpoint for verifying the robustness of findings towards different assumptions. These included an analysis where all patients with missing parasitaemia were treated as failures, an analysis where patients with new infections as detected by PCR were excluded and an analysis on two enlarged per protocol populations where all patients/all early failures “not receiving at least 80% of study treatment” or “failing to attend a visit at days 0–2” were not excluded.

The true treatment failures defined in accordance with the WHO handbook were analysed by means of the survival analysis (Kaplan-Meier estimates). All sources of uncertainty (i.e. withdrawals, new infections, patients with PCR results either not available or indeterminate) were censored. Survival analysis techniques were also applied to the analysis of time to parasite clearance and the estimation of the rate of new infections (in the latter analysis recrudescent infections were censored). For descriptive purposes, the proportions of early, late and true treatment failures were also computed as simple rates for each treatment arm together with the relevant CIs.

By-country heterogeneity in cure rates was assessed by the Breslow-Day test or by logistic regression, when the former was not applicable. The study was conducted over two malaria seasons with different centres working in the two study periods. To evaluate how the 63-day PCR-corrected cure rates varied across the cohorts from the two seasons, a logistic regression model was fitted with cohort and treatment as the explanatory variables. The treatment by cohort interaction was evaluated as a candidate to enter this model through a residual score test. All tests for heterogeneity and interaction were evaluated at the 10% significance level.

Rates of person-fever-days and person-gametocyte-weeks were calculated as the number of weeks in which fever was present or blood slides were positive for gametocytes, respectively, divided by the number of follow-up weeks and expressed per 100 person-weeks.

The safety population, which coincides with the ITT population, was used for all safety assessments. Adverse events were coded using the MedDRA dictionary (MedDRA V8.1). Proportions of patients experiencing at least one adverse event were compared between treatments using the Pearson Chi square test.

To determine the sample size of this study, the PCR-corrected cure rate at day 63 was estimated to be at least 92% for AS+MQ in the ITT population using a literature search [30]–[35]. Expert opinion was used to define the non-inferiority margin, which was set at 5%. The planned sample size of 700 in the DHA/PQP arm and 350 in the AS+MQ arm (1050 patients in total) provided 80% power to show non-inferiority with a non-inferiority margin of −5% (test minus reference) and a one-sided α level of 2.5%. The rate of patient attrition in the per protocol population was expected to be 20% compared with the ITT population, but the PCR-corrected cure rate was expected to be higher than in the ITT population (95% in the per protocol population), therefore the projected power of 80% was maintained also for the analysis on the per protocol population. When India was included in the study to increase the speed of recruitment, the total sample size was increased by 150 patients to ensure that 100 Indian patients were treated with DHA/PQP, in accordance with Indian requirements. Consequently, the power of the study exceeded 80%.

## Results

### Patient disposition and demographic characteristics

**Figure 1:**
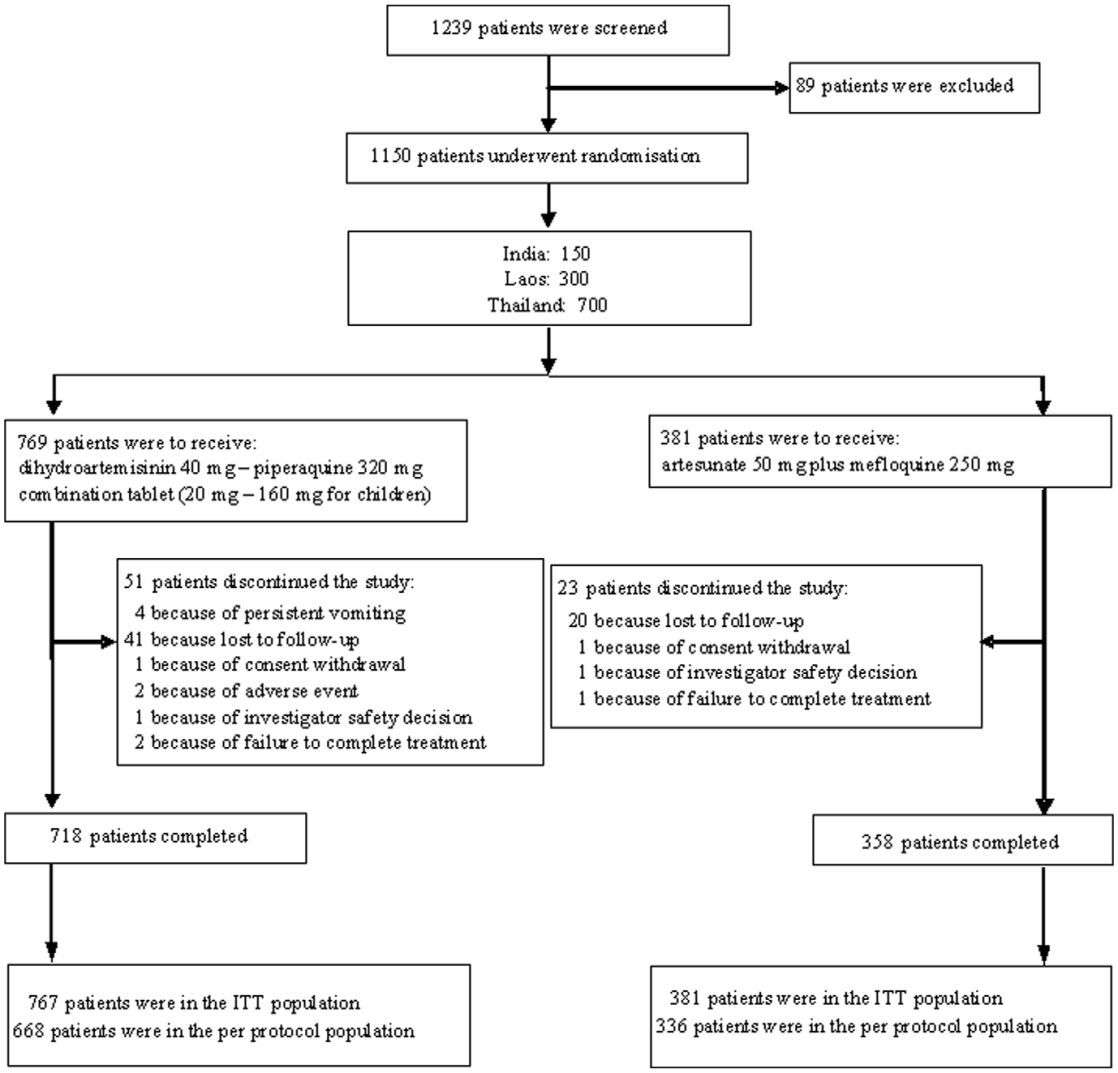
Flow chart of patient disposition.

One thousand two hundred and thirty-nine patients were screened, with 769 patients randomised to DHA/PQP and 381 patients randomised to AS+MQ. Figure 1 illustrates the number of patients completing the study and included in the different study populations. Sixty-one percent of patients were recruited in Thailand, 26% in Laos and 13% in India (Table 2).

**Table 2:** Baseline characteristics (ITT population).

Variable	DHA/PQP	AS+MQ
	N = 767	N = 381
Sample size by country; n (%)		
Thailand	466 (61)	234 (61)
Laos	200 (26)	98 (26)
India	101 (13)	49 (13)
Gender; n : n		
Male : Female	582∶185	295∶86
Age; years		
Mean ± SD	25.4±13.3	25.8±13.7
≤5 years, n (%)	57 (7)	32 (8)
>5–≤12 years, n (%)	68 (9)	31 (8)
>12–≤18 years, n (%)	76 (10)	31 (8)
>18–≤64 years, n (%)	566 (74)	287 (75)
Weight; kg		
Mean ± SD	44.3±15.1	44.6±15.1
Race; n (%)		
Asian	767 (100)	381 (100)
Presence of fever		
n (%)	509 (66)	258 (68)
Temperature in °C		
Mean ± SD	37.9 (1.01)	37.9 (1.02)
Parasite density		
Geometric mean	7923.8	9735.4
Haemoglobin; g/L		
Normal range (min - max)*: (105–180)		
Mean ± SD	118.2±24.5	120.0±23.2
n Missing (%)	4 (0.52)	2 (0.52)
n <100 g/L (%)	162 (21.12)	72 (18.90)
n≥100 g/L (%)	601 (78.36)	307 (80.58)
White cells; ×10^9^/L		
Normal range (min - max)*: (3.6–11.0)		
Mean ± SD	6.3±2.6	6.3±2.3
Platelets; ×10^9^/L		
Normal range (min - max)*: (140–500)		
Mean ± SD	127.7±70.6	124.8±65.5
ALT; U/L		
Normal range (min - max)*: (0–50)		
Mean ± SD	31.1±29.3	32.9±41.5
Total bilirubin; mg/dl		
Normal range (min - max)*: (0–1.5)		
Mean ± SD	1.19±0.85	1.20±0.78
Creatinine; µmol/L		
Normal range (min - max)*: (0–150.28)		
Mean ± SD	75.5±28.7	76.2±30.7
**Study populations; n (%)**		
Safety/ITT	767 (99.7)	381 (100.0)
Per protocol	668 (86.9)	336 (88.2)

DHA/PQP  =  dihydroartemisinin-piperaquine; AS+MQ  =  artesunate-mefloquine; SD  =  standard deviation; Hb  =  haemoglobin; ALT  =  alanine aminotransferase.

*Since the normal laboratory reference ranges vary across centres, the minimum of the lower limits and the maximum of the upper limits are reported.

There were no notable differences in demographic parameters between treatment arms overall or by country (Table 2). Children ≤5 years of age comprised approximately 8% of the study population, with most of the study population over 18 years of age (approximately 75%). Median [range] doses received by patients of the ITT population are presented by age class in Table 3.

**Table 3:** Doses (mg/kg) received for each age class (ITT population).

Age range (years)	Doses received over 3 days; median [range]			
	DHA	PQP	AS	MQ
≤5	6.7 [1.8–9.2]	53.3 [14.5–73.8]	12.5 [10.7–13.6]	24.0 [21.7–27.8]
>5–≤12	7.2 [5.0–10.0]	57.3 [40.0–80.0]	12.5 [10.7–13.2]	25.0 [22.3–26.3]
>12–≤18	7.9 [2.9–9.7]	63.3 [22.9–77.8]	12.0 [11.4–12.5]	25.0 [24.2–31.3]
>18–≤64	7.1 [2.1–10.0]	56.5 [16.8–80.0]	12.0 [3.9–12.5]	25.0 [0.0–26.5]

DHA  =  dihydroartemisinin; PQP  =  piperaquine; AS  =  artesunate; MQ  =  mefloquine.

One hundred and forty-four patients were excluded from the per protocol population (Table 2). There were no notable differences between the treatment arms in the number of patients excluded from the per protocol population, the most frequent reasons for exclusion were lost to follow-up before or at day 63 and PCR missing or indeterminate before or at day 63 (DHA/PQP: 35 patients: AS+MQ: 19 patients).

### Primary Endpoint

The analysis of the PCR-corrected cure rate at day 63 confirmed that DHA/PQP was non-inferior to AS+MQ. For the ITT population, PCR-corrected cure rates were 87.9% for DHA/PQP and 86.6% for AS+MQ (97.5% CI: >−2.87%; Table 4). As expected, better absolute but comparatively similar results were obtained for the per protocol population with PCR-corrected cure rates of 98.7% for DHA/PQP and 97.0% for AS+MQ (97.5% CI: >−0.39%). Sensitivity analyses confirmed these results, with similar treatment differences between cure rates and relevant CIs compared with those described above.

**Table 4:** Polymerase chain reaction (PCR)-corrected and uncorrected cure rates (ITT and per protocol populations).

	DHA/PQP	AS+MQ	Lower limit of the 97.5%	p-value^2^
	% (n)	% (n)	(one-sided) CI^1^	
**ITT**	**N = 767**	**N = 381**		
Day 63				
PCR-corrected cure rate	87.9 (674)	86.6 (330)	−2.87	0.544
Uncorrected cure rate	67.3 (516)	59.6 (227)	1.75	0.010
Day 42				
PCR-corrected cure rate	90.5 (694)	88.2 (336)	−1.56	0.228
Uncorrected cure rate	83.2 (638)	77.4 (295)	0.79	0.019
Day 28				
PCR-corrected cure rate	93.7 (719)	91.9 (350)	−1.36	0.236
Uncorrected cure rate	92.3 (708)	88.2 (336)	0.37	0.022
**Per protocol**	**N = 668**	**N = 336**		
Day 63				
PCR-corrected cure rate	98.7 (659/668)	97.0 (326/336)	−0.39	0.074
Uncorrected cure rate	75.5 (504/668)	66.4 (223/336)	3.07	0.002
Day 42				
PCR-corrected cure rate	99.3 (663)	97.6 (328)	−0.12	0.031
Uncorrected cure rate	91.2 (609)	85.4 (287)	1.41	0.006
Day 28				
PCR-corrected cure rate	99.9 (667)	97.9 (329)	0.38	0.001
Uncorrected cure rate	98.2 (656)	93.8 (315)	1.68	<0.001

DHA-PQP  =  dihydroartemisinin-piperaquine; AS+MQ  =  artesunate-mefloquine; CI  =  confidence interval.

1 Confidence interval for the difference DHA/PQP minus AS+MQ.

2 Chi-squared test.

### Secondary endpoints

Non-inferiority was also proved for the uncorrected cure rates at day 63: the cure rates were 67.3% in the DHA/PQP arm and 59.6% in the AS+MQ arm (ITT population) with a CI >1.75%. This treatment difference was also statistically significant (Chi-squared test: p = 0.010; Table 4). Similar results were obtained for the per protocol population (Table 4).

Significantly more patients who received AS+MQ experienced new infections compared with those receiving DHA/PQP. Kaplan-Meier estimates for the proportion of patients with new infection at day 63 were 22.7% for DHA/PQP and 30.3% for AS+MQ (ITT population; p = 0.0042). Similar results were obtained for the other populations.

Non-inferiority of DHA/PQP versus AS+MQ was also shown at days 28 and 42 in all study populations (Table 4). On days 28 and 42, in the per protocol population, the PCR-corrected cure rates defined in Table 1 were significantly greater for DHA/PQP than AS+MQ (p = 0.001 and p = 0.031, respectively) while these differences were not statistically significant in the ITT population (Table 4).

**Figure 2:**
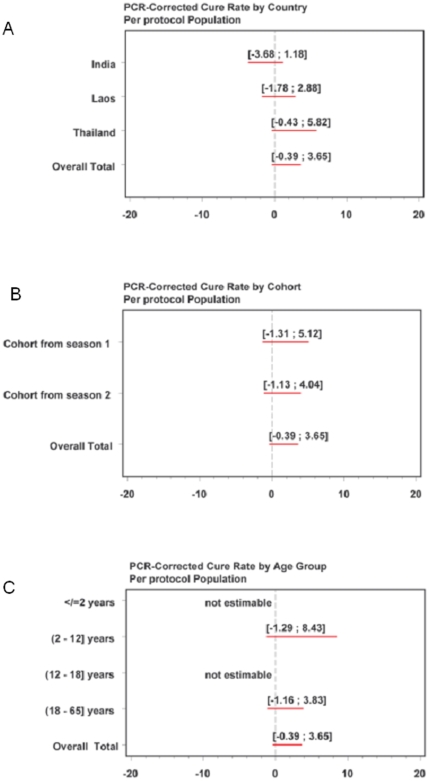
Ninety-five percent (two-sided) confidence intervals of PCR-corrected cure rates in the ITT population, by country, cohort of enrolment from the two seasons and age group.

A logistic regression test to assess by-country heterogeneity in the PCR-corrected cure rates (evaluated according to Table 1) showed no significant differences between countries (ITT: p = 0.688; per protocol: p = 0.988;). Similar results were obtained for a Breslow-Day test conducted to assess by-country heterogeneity in the uncorrected cure rate (ITT: p = 0.896; per protocol: p = 0.728). The 95% (two-sided) CIs at each individual country level for the PCR-corrected cure rate at day 63 for the per protocol population are shown in Figure 2A. No effect due to the cohorts from the two seasons (per protocol: p = 0.760) was shown in this study (Figure 2B shows the relevant CIs) and no difference was observed by age class (per protocol: p = 0.998). Classification by age was: ≤2 years; 2–12 (included) years; 12–18 (included) years and >18 years (relevant CIs are shown in Figure 2C). Results in the other populations for country, cohort (season) of enrolment and age groups were similar.

When the PCR-corrected cure rates were assessed and analysed according to the WHO recommendations, i.e. as 100 minus the true treatment failure rate, the results were similar to those in the PCR-corrected cure rate as defined in Table 1, although cure rates were always slightly higher. The Kaplan-Meier estimates of the cure rates were 97.6% (DHA/PQP) versus 96.5% (AS+MQ) in the ITT population and 98.2% (DHA/PQP) versus 96.8% (AS+MQ) in the per protocol population. The simple rates of early, late and true treatment failures on days 63, 42 and 28 are shown in Table 5 together with the relevant CIs.

**Table 5:** Early, late and true treatment failure rates^1^ as defined by WHO (ITT and per protocol populations).

	DHA/PQP	AS+MQ	95% CI
	% (n)	% (n)	
**ITT**			
Early treatment failures	0.52 (4)	0.26 (1)	−0.46, 0.98
Day 63			
Late treatment failures	13.17 (101)	14.96 (57)	−6.10, 2.52
True treatment failures	2.09 (16)	2.62 (10)	−2.44, 1.36
Day 42			
Late treatment failures	5.74 (44)	9.71 (37)	−7.37, −0.58
True treatment failures	1.17 (9)	2.10 (8)	−2.56, 0.70
Day 28			
Late treatment failures	1.17 (9)	5.25 (20)	−6.44, −1.71
True treatment failures	0.65 (5)	1.84 (7)	−2.65, 0.28
**Per protocol**			
Early treatment failures	0	0.30 (1)	−0.89, 0.28
Day 63			
Late treatment failures	12.87 (86)	15.48 (52)	−7.23, 2.02
True treatment failures	1.65 (11)	2.98 (10)	−3.39, 0.73
Day 42			
Late treatment failures	5.84 (39)	9.82 (33)	−7.63, −0.34
True treatment failures	0.75 (5)	2.38 (8)	−3.39, 0.12
Day 28			
Late treatment failures	0.90 (6)	4.76 (16)	−6.25, −1.48
True treatment failures	0.15 (1)	2.08 (7)	−3.49, −0.38

1 The rates reported in this table are simple rates.

Kaplan-Meier estimates of median time to parasite clearance were 2 days for each treatment (ITT population), while the Kaplan-Meier estimate of the rate of aparasitaemic patients at day 3 (i.e. 24 h after completion of study treatment) was 97.6% for DHA/PQP and 97.6% for AS+MQ. Similar results were obtained for the per protocol population. Proportions of afebrile patients showed very similar profiles to aparasitaemic patients, with profiles being virtually superimposable. Fever incidence measured in person-fever-days (per 100 person-days) was similar for DHA/PQP and AS+MQ: 1065/5315 (20.04%) versus 524/2636 (19.88%), p = 0.929 (Table 6; ITT population).

**Table 6:** Prevalence of gametocytes and fever according to day of follow-up (ITT population).

	DHA/PQP	AS+MQ	p-value^1^
	N = 767	N = 381	
	n/N (%)	n/N (%)	
**Gametocyte prevalence** 2			
Day 7	59/749 (7.88)	15/369 (4.07)	0.016
Day 14	30/742 (4.04)	3/365 (0.82)	0.003
Day 21	16/733 (2.18)	0/362	0.005
Day 28	9/722 (1.25)	0/353	0.035
Day 35	1/715 (0.14)	0/339	1.000
Day 42	1/692 (0.14)	0/328	1.000
Day 49	2/666 (0.30)	0/316	1.000
Day 56	1/651 (0.15)	1/312 (0.32)	0.543
Day 63	1/623 (0.16)	2/301 (0.66)	0.249
Overall (from day 7 up to day 63)	76/749 (10.15)	18/369 (4.88)	0.003
Person-gametocye-weeks^3^ (/100 person-weeks)	130/6420 (2.02)	23/3108 (0.74)	0.014
**Fever prevalence** 2			
Day 0	509/767 (66.36)	258/381 (67.72)	0.646
Day 1	244/767 (31.81)	129/381 (33.86)	0.486
Day 2	80/765 (10.46)	44/379 (11.61)	0.555
Day 3	51/764 (6.68)	21/379 (5.54)	0.457
Day 7	40/753 (5.31)	16/373 (4.29)	0.458
Overall (from day 0 up to day 7)	566/767 (73.79)	298/381 (78.22)	0.102
Person-fever-days^4^ (/100 person-days)	1065/5315 (20.03)	524/2636 (19.88)	0.929

DHA/PQP  =  dihydroartemisinin-piperaquine; AS+MQ  =  artesunate-mefloquine.

1 Pearson Chi-square or Fisher's exact test, as appropriate.

2 Calculated, at a given time, as number of patients with gametocytes, or fever ≥37.5°C, at that time divided by the number of patients having reached that time.

3 Calculated as number of weeks in which blood slides were positive for gametocytes during the whole study (up to a maximum duration of 70 days) divided by the number of all follow-up weeks and expressed per 100 person-weeks. Withdrawal patients were analysed up to the date of withdrawal recorded in the efficacy dataset even if they performed additional gametocyte assessments.

4 Calculated as number of days in which temperature was greater or equal to 37.5 during the first study week divided by the number of all first follow-up weeks and expressed per 100 person-days. Withdrawal patients were analysed up to the date of withdrawal recorded in the efficacy dataset even if they performed additional assessments for temperature.

The overall gametocyte prevalence throughout the study (from day 7 to day 63), measured as person-gametocyte-week rates, was significantly higher in the DHA/PQP arm in all populations (DHA/PQP: 76/749, 10.15%; AS+MQ: 18/369, 4.88%; p = 0.003; Table 6, ITT population). However, the treatment difference for prevalence was not statistically significant from day 35 onwards (Table 6; ITT population).

There were no deaths during the study. In the DHA/PQP group, there were 12 (1.56%) events judged by the investigator to be serious, which occurred in 12 patients. Six (0.78%) of these events were considered by the investigators to be related to DHA/PQP. These were two cases of anaemia, one from day 7 to day 90, the other from day 7 to day 35; one viral infection (possibly Dengue fever) from day 15, fully recovered; and one Wolf Parkinson White (WPW) syndrome from day 2 to day 90. Wolf Parkinson White syndrome is a congenital defect of accessory conduction pathways. Electrocardiograms for this patient were submitted to two cardiologists to provide expert opinions. Both considered that the failure to diagnose WPW at baseline could be attributed to the increased heart rate caused by the patients' fever which hid the electrocardiographic characteristics of WPW. Other events considered serious and related were one convulsion on day 0, and one encephalitis on day 45 which resulted in a left-sided hemiplegia. The patient was diagnosed with *P. falciparum* malaria on day 48, which was treated with i.v. artesunate and oral mefloquine. The other six (0.8%) serious events were judged unrelated to DHA/PQP by the investigator: two cases of pyelonephritis, one case of aspiration pneumonia, and three cases of *P. falciparum* malaria. In the AS+MQ group three (0.8%) events in three patients were judged to be serious and all were judged related to study treatment: one case of anaemia from day 8, one case of convulsion on day 1, and one case of encephalitis from day 16 to day 31.

Adverse event profiles for DHA/PQP and AS+MQ were very similar in terms of type and frequency of events and were consistent with those expected in adult patients with acute malaria. Most patients in the study experienced adverse events: 69.4% (532/767) of patients in the DHA/PQP arm and 72.4% (276/381) of patients in the AS+MQ arm. There was no statistically significant difference between the treatments in the incidence of adverse events (p = 0.282, Chi-square test). The most frequently reported adverse events were malaria symptoms, with headache the most commonly reported (Table 7). The frequencies of individual adverse events were generally similar between treatments, although the frequencies of nausea, vomiting and dizziness appeared to be higher in the AS+MQ arm (Table 7). Approximately 3% of patients in each arm experienced at least one adverse event related to skin or subcutaneous tissue; the most frequent was pruritus (DHA/PQP: 17 patients [2.2%]; AS+MQ: 8 patients [2.1%]). One patient in each arm experienced allergic dermatitis.

**Table 7:** Most frequently reported adverse events (>5% of either treatment arm; ITT population).

Event	DHA/PQP	AS+MQ	Chi-Square
	N = 767	N = 381	p-value
	n (%)	n (%)	
Headache	138 (18.0)	77 (20.2)	0.3644
Malaria^1^	111 (14.5)	86 (22.6)	0.0006
*P. falciparum* infection	103 (13.4)	58 (15.2)	0.4097
Pyrexia	81 (10.6)	43 (11.3)	0.7092
Eosinophilia	65 (8.5)	38 (10.0)	0.4026
Cough	60 (7.8)	37 (9.7)	0.2786
Anaemia	55 (7.2)	25 (6.6)	0.7027
Myalgia	46 (6.0)	22 (5.8)	0.8801
Arthralgia	42 (5.5)	21 (5.5)	0.9799
Prolonged QTc interval	41 (5.4)	16 (4.2)	0.3999
Abdominal pain	40 (5.2)	20 (5.3)	0.9804
Asthenia	38 (5.0)	29 (7.6)	0.0705
Anorexia	38 (5.0)	21 (5.5)	0.6871
Nausea	22 (2.9)	26 (6.8)	0.0016
Vomiting	19 (2.5)	24 (6.3)	0.0013
Dizziness	11 (1.4)	24 (6.3)	<.0001

DHA/PQP  =  dihydroartemisinin-piperaquine; AS+MQ  =  artesunate-mefloquine.

1 Reporting of malaria as an adverse event was not complete in this study. Some study centres chose not to report malaria as it was known that to enter the study all patients had to have *Plasmodium falciparum* infection.

As would be expected in patients with malaria, anaemia and thrombocytopenia were common in each arm, approximate proportions were 30% for low red blood cells (normal range for >15 years of age: 3.5−5.5×10^12^/L for women and 4−5.5×10^12^/L for men), 50% for low haemoglobin (normal range for >15 years of age: 110−150 g/L for women and 120−160 g/L for men), and 67% for low platelets (normal range for all ages: 100−300×10^9^/L). At the end of the study following treatment, these proportions had decreased to approximately, 20%, 40% and 17%, respectively. There was no apparent difference between treatments. Increases in mean haemoglobin levels were observed over the 63-day follow-up period. In the ITT population, mean (standard deviation) haemoglobin values on day 0 were 118.7 (24.4) g/L for DHA/PQP and 119.8 (23.4) g/L for AS+MQ. At day 63, mean (standard deviation) changes from day 0 were 12.8 (22.2) g/L for DHA/PQP and 14.21 (21.2) g/L for AS+MQ. Similar results were observed for the per protocol population. Other than elevated liver parameters, as might be expected in this population, there were no relevant changes in biochemistry parameters.

At baseline, using the QTc_(B)_ method, there was a statistically significant difference between treatments (Mantel-Haenszel Chi-square test, p = 0.026), with a higher proportion of patients in the DHA/PQP group having borderline QTc_(B)_ values (16.6% vs. 12.2%; p = 0.066). No statistically significant difference between treatments was observed at baseline for QTc_(F)_ (2.9% for DHA/PQP vs. 1.6% in AS+MQ). On day 2, there was a statistically significant difference between treatments (Mantel-Haenszel Chi-square test p<0.001), with a higher proportion of patients in the DHA/PQP group having borderline (21.4%; p = 0.043) or prolonged (8.6%; p = 0.007) QTc_(B)_ intervals than in the AS+MQ group (16.3% and 4.2%, respectively). This difference was also observed for the QTc_(F)_ method: borderline, 13.0% for DHA/PQP vs. 5.3% for AS+MQ (p<0.001); prolonged, 4.7% for DHA/PQP vs. 5.3% for AS+MQ (p<0.001). By day 7, there was no difference between treatments.

The proportion of patients with QTc_(B)_ increase >60 msec from baseline to day 2 was 0.9% for DHA/PQP vs. 0.8% for AS+MQ (not significant), and 4.6% for DHA/PQP vs. 2.9% for AS+MQ when the same increase was assessed with QTc_(F)_ (p<0.001). However, QTc and QT prolongation were reported as adverse events by 43 (5.6%) patients in the DHA/PQP group and 16 (4.20%) patients in the AS+MQ group; these were judged by the investigator to be related to study treatment for 28 (3.65%) patients in the DHA/PQP group and 13 (3.41%) patients in the AS+MQ group.

Mean QTc_(F)_values on day 0 were 387.70 msec for DHA/PQP and 385.54 msec for AS+MQ. Mean increases from baseline to day 2 were 22.93 msec and 14.65 msec, respectively. This difference between treatments was statistically significant (p <0.001). On day 7, the mean increase from day 0 for DHA/PQP had fallen to 10.47 msec with the value for AS+MQ being 13.39 msec; this difference was not statistically significant (p = 0.075).

## Discussion

In this study conducted in Thailand, Laos and India, we have shown that both the DHA/PQP and AS+MQ treatment combinations are efficacious treatments of *P. falciparum* malaria. The day-63 PCR-corrected cure rates (as defined in Table 1) were 87.9% for DHA/PQP and 86.6% for AS+MQ in the ITT population and 98.7% for DHA/PQP and 97.0% for AS+MQ in the per protocol population. In terms of this primary outcome variable, DHA/PQP was non-inferior to AS+MQ. Review of the data by country found no differences between the primary outcome measures, further confirming that DHA/PQP was similarly active against the *P. falciparum* found in India, Laos and Thailand, relative to AS+MQ. The PCR-corrected cure rates observed for DHA/PQP in this study were in line with the day-63 PCR-corrected cure rates noted in a previous study conducted in Thailand [32], day-42 rates observed in Myanmar (Burma) [11] and Laos [36] and day-28 cure rates observed in Cambodia [8]. Likewise, PCR-corrected cure rates for AS+MQ were also similar to previous studies of AS+MQ conducted in Thailand [10], [37]–[39], Laos [40], [41] and India [38], which generally showed rates ranging from approximately 95% to 100% in the per protocol population. Polymerase chain reaction-corrected cure rates for both DHA/PQP and AS+MQ exceeded 95% on days 28, 42 and 63. The 95% threshold on day 28 is the level the WHO recommend for adoption of a new treatment [14].

This is the first study of the use of DHA/PQP in the Indian population, with 101 patients receiving the combination. Although this was only 13% of the patients in our study, we feel that it provides sufficient numbers to enable an initial assessment of the response to DHA/PQP in communities in Assam, Goa and Mangalore. India, Thailand and Laos have different backgrounds in terms of parasite transmission, resistance and seasonality of infection, yet a heterogeneity analysis showed no statistically significant difference in the relative responses to the two treatments between India and the other countries in the study. Indian communities that we sampled in this study had notable levels of chloroquine resistance, with historical site records showing treatment failure rates for chloroquine treatment ranging from 32% to 67% (28 days of follow-up). The sites in Laos and Thailand were also in regions with high levels of chloroquine resistance [22], [24], [25], [42]. Based on the structural similarities of chloroquine and PQP, there was the potential for cross-resistance and a low response to DHA/PQP may have been expected. However, the DHA/PQP combination appeared to be unaffected by any potential cross-resistance and PCR-corrected cure rates were similar for India (96.5%), Thailand (96.6%) and Laos (98.0%).

The DHA/PQP combination exerted a significant post-treatment prophylactic effect in this study. This is supported by, first, significant reductions in the incidence of new infections for DHA/PQP compared with AS+MQ and second, by higher uncorrected cure rates in the DHA/PQP arm than AS+MQ, as the uncorrected cure rate endpoint includes new infections as well as recurrence of infection. This effect is thought to be modulated by levels of drug remaining in the blood because of the long half-life of PQP. The post-treatment prophylaxis was significantly better for DHA/PQP reflecting the differential terminal half lives of PQP and MQ (4–5 weeks for PQP and 14 days for MQ). The extended post-treatment prophylactic effect is of particular importance in countries with a high risk of new infection and can also reduce the risk of anaemia by allowing patients more time for haematological recovery between infections. The DHA/PQP combination, like all ACTs, rapidly reduces parasite biomass in the patient through the brief yet potent activity of DHA (the artemisinin component). Subsequent removal of uncleared parasites is achieved by the less active but more slowly eliminated partner drug; in this case PQP with a half-life of 4–5 weeks [43], [44]. The slightly shorter half-life of MQ [45] may explain the difference observed in the post-treatment prophylactic effect of the two regimens. The DHA/PQP regimen has previously been shown to exert a superior post-treatment prophylactic effect to another ACT, artemether-lumefantrine [46]. Overall gametocyte prevalence was significantly higher in the DHA/PQP arm than AS+MQ. The viability of gametocytes remaining post-treatment was not assessed in this study. One potential point of interest for future studies would be to determine whether the viability of any remaining gametocytes is compromised by treatment with DHA/PQP.

Although formal statistical comparison of the tolerability profile of the two combinations was not planned or conducted, some individual adverse events appeared to be reported more frequently in patients receiving AS+MQ than those receiving DHA/PQP. Mefloquine has been linked with adverse events of gastrointestinal and central nervous system origin. In this study, the frequency of reporting of nausea, vomiting and dizziness was 2–3 fold higher in the AS+MQ arm than the DHA/PQP arm. Vomiting soon after dosing is an important determinant of treatment efficacy as attaining and maintaining effective systemic levels of anti-malarial drugs is essential to disease outcome. This is of particular importance where there are powerful influences on adherence to dosing regimens. Increases in QTc_(F)_ interval were seen for both DHA/PQP and AS+MQ after the start of treatment, but a statistically significant increase from baseline was observed in QTc interval for DHA/PQP relative to AS+MQ on day 2. An important element in assessing the clinical importance of QTc prolongation is the extent of change from baseline: an increase in QTc interval from baseline of >60 msec could be clinically significant and this is the recognised threshold for drug-induced arrhythmias in non-cardiovascular indications (ICH E14 guideline). There was a statistically significant treatment difference for the proportion of patients with such an increase in QTc_(F)_, while no treatment differences were observed for the same increase in QTc_(B)_ and for the incidence of cardiac events. Finally, by day 7 there were no significant differences between treatments. Based on our results, it is difficult to attribute a particular clinical relevance to the QTc increase observed with DHA/PQP. In fact, it is expected that malaria shortens the QT interval during the acute illness, leading to increases after the start of treatment. Most studies of antimalarial drugs report some transient prolongation of the QT interval in the days after the start of treatment [47]. No warnings were found in the literature about QTc prolongation with DHA/PQP.

For patients to access DHA/PQP via public sector healthcare systems, this fixed dose combination requires regulatory approval. One requirement for registration is that the formulation must be manufactured according to GMP. This study was conducted as one of a series of pharmacokinetic and ICH-GCP-compliant randomised, controlled trials at sites across Africa and South East Asia using DHA/PQP manufactured according to GMP.

In conclusion, the fixed dose DHA/PQP combination tablet in this study emerged as a highly efficacious treatment for *P. falciparum* malaria. The effects were observed across the three Asian countries in which the study was conducted, with no country effect. The combination of DHA/PQP provided greater protection against new infections than AS+MQ. Results from this study indicate that, although there may be evidence that suggests that the overall efficacy of ACTs may be falling, DHA/PQP can play an important role in, possibly, a new policy of multiple first-line treatments of uncomplicated falciparum malaria.

## Supporting Information

Checklist S1 CONSORT checklist.
(0.19 MB DOC)

Protocol S1 Trial protocol.
(1.32 MB PDF)

Protocol S2 Protocol amendment 1.
(0.14 MB PDF)

Protocol S3 Protocol amendment 2.
(0.23 MB PDF)

Protocol S4 Protocol amendment 3.
(0.19 MB PDF)

Protocol S5 Protocol amendment 3A.
(0.08 MB PDF)

Protocol S6 Protocol amendment 3B.
(0.09 MB PDF)

Protocol S7 Protocol amendment 4.
(0.09 MB PDF)

Protocol S8 Protocol amendment 5.
(0.10 MB PDF)
