# Correction: An Open-Label, Randomised Study of Dihydroartemisinin-Piperaquine Versus Artesunate-Mefloquine for Falciparum Malaria in Asia

**DOI:** 10.1371/annotation/73589846-034d-4b6f-9668-54f278fa03e8

**Published:** 2010-09-09

**Authors:** Neena Valecha, Aung Pyae Phyo, Mayfong Mayxay, Paul N. Newton, Srivicha Krudsood, Sommay Keomany, Maniphone Khanthavong, Tiengkham Pongvongsa, Ronnatrai Ruangveerayuth, Chirapong Uthaisil, David Ubben, Stephan Duparc, Antonella Bacchieri, Marco Corsi, Bappanad H. K. Rao, Prabash C. Bhattacharya, Nagesh Dubhashi, Susanta K. Ghosh, Vas Dev, Ashwani Kumar, Sasithon Pukrittayakamee

The last author's name was spelled incorrectly. The correct name is: Sasithon Pukrittayakamee. 

