# Correction: An Open-Label, Randomised Study of Dihydroartemisinin-Piperaquine Versus Artesunate-Mefloquine for Falciparum Malaria in Asia

**DOI:** 10.1371/annotation/5603e1d7-03ca-4127-ab99-1d1e07d905d4

**Published:** 2010-09-09

**Authors:** Neena Valecha, Aung Pyae Phyo, Mayfong Mayxay, Paul N. Newton, Srivicha Krudsood, Sommay Keomany, Maniphone Khanthavong, Tiengkham Pongvongsa, Ronnatrai Ruangveerayuth, Chirapong Uthaisil, David Ubben, Stephan Duparc, Antonella Bacchieri, Marco Corsi, Bappanad H. K. Rao, Prabash C. Bhattacharya, Nagesh Dubhashi, Susanta K. Ghosh, Vas Dev, Ashwani Kumar, Sasithon Pukrittayakamee

The second sentence in the Methods and Findings section of the Abstract contained an error. The sentence should read: Patients aged 3 months to 65 years with *Plasmodium falciparum* mono-infection or mixed infection were randomised with an allocation ratio of 2:1 to a fixed-dose DHA/PQP combination tablet (adults: 40 mg/320 mg; children: 20 mg/160 mg; n = 769) or loose combination of AS+MQ (AS: 50 mg, MQ: 250 mg; n = 381). 

