# Supplementary material for: An Open-Label, Randomised Study of Dihydroartemisinin-Piperaquine Versus Artesunate-Mefloquine for Falciparum Malaria in Asia
Source: PLoS One. 2010 Jul 30;5(7):e11880. doi: 10.1371/journal.pone.0011880 (PMC2912766; doi:10.1371/journal.pone.0011880)

σ sigma-tau
Industrie Farmaceutiche Riunite S.p.A.
Sede Legale: Roma, V.le Shakespeare 47
Cap. Sociale: 30.500.000.000 inter. versato
Cod. Fiscale: 00410650584
P. IVA n°: 00885531004
Canc. Comm. Trib. di Roma n° 1468/57
Iscriz. Sch. Naz. Ric. Cod. G0120YKG
c.c.i.a.a.: Roma n° 205785

MEDICAL DEPARTMENT - *Research & Development Department*

Artekin

Study Protocol No.: ST3073-ST3074 DM040010

(Final Version – 10th January, 2005)

A Phase III, randomized, non-inferiority trial, to assess the efficacy and safety of Dihydroartemisinin+Piperaquine (DHA+PPQ, Artekin) in comparison with Artesunate+Mefloquine (AS+MQ) in patients affected by acute, uncomplicated *Plasmodium falciparum* malaria.

# - MULTICENTRE STUDY IN ASIA-

Co-ordinating Investigator
Professor Nicholas J White
Wellcome Trust Southeast Asian Tropical Medicine Research Units
Faculty of Tropical Medicine Mahidol University
420/6 Rajvithi Rd, Phayathai 10400
Bangkok, THAILAND

**Protocol No.:**	ST3073-ST3074 DM040010
**Date:**	January 10th, 2005
**Version:**	Final version
**Title:**	A Phase III, randomized, non-inferiority trial, to assess the efficacy and safety of Dihydroartemisinin+Piperaquine (DHA+PPQ, Artekin™) in comparison with Artesunate+Mefloquine (AS+MQ) in patients affected by acute, uncomplicated Plasmodium *falciparum* malaria.
**Countries :**	3 countries: Thailand, China and Laos
**Sites :**	5 sites in Thailand, 1 in China, 1 in Laos
**Product:**	Dihydroartemisinin+Piperaquine (DHA+PPQ, Artekin™)
**Pharm. Form.:**	Tablets containing 40 mg of Dihydroartemisinin and 320 mg of Piperaquine for adult patients or containing 20 mg of Dihydroartemisinin and 160 mg of Piperaquine for infants and children.
**Control Drug:**	Artesunate+Mefloquine
**Pharm. Form.:**	Tablets containing 50 mg of Artesunate and tablets containing 250 mg of Mefloquine
**Sponsor:**	Sigma-Tau i.f.r. S.p.A., Pomezia (Rome) - Italy
**Co-ordinating Investigator:**	Professor Nick White
**Address:**	Wellcome Trust Southeast Asian Tropical Medicine Research Units, Faculty of Tropical Medicine Mahidol University, 420/6 Rajvithi Rd, Phayathai 10400, Bangkok, THAILAND
**Telephone:**	66 2 3549172
**Fax:**	66 2 3549169

STUDY ACKNOWLEDGMENT/CONFIDENTIALITY

By signing this protocol, the Investigator(s) acknowledges and agrees:

The protocol contains all necessary information for conducting study. The Investigator will conduct this study as detailed herein and will make every reasonable effort to complete the study within the time designated.

The protocol and all relevant information on the drug relating to pre-clinical and prior clinical experience, which was provided by the Sponsor, will be made available to all physicians, nurses and other personnel who participate in conducting this study. The Investigator will discuss this material with them to assure that they are fully informed regarding the drug(s) and the conduct of the study.

This document contains information that is privileged or confidential. As such, it may not be disclosed unless specific permission is granted in writing by Sigma-Tau i.f.r. S.p.A., Pomezia (Rome) - Italy or such disclosure is required by federal or other laws or regulations. In any event, persons to whom the information is disclosed must be informed that the information is privileged or confidential and may not be further disclosed by them. These restrictions on disclosure will apply equally to all future information supplied which is indicated as privileged or confidential.

Sigma-Tau i.f.r. S.p.A., Pomezia (Rome) - Italy will have access to any source documents from which Case Report Form information may have been generated. The Case Report Forms and any other data pertinent to this study are the property of Sigma-Tau i.f.r. S.p.A., Pomezia (Rome) - Italy, who may utilise the data in various ways, such as for submission to government regulatory authorities, or in publication of the results of this multicentre study.

The conduct and results of this study will be kept confidential until all sites have completed the study, unless an interim publication or presentation is agreed upon. The results of this multicentre study will be published. Furthermore, each Investigator may independently publish the data at that particular site. However, to prevent premature disclosure of confidential information, the timing of a separate presentation or publication of the study by the Investigator will be subject to mutual agreement in advance between Sigma-Tau i.f.r. S.p.A., Pomezia (Rome) - Italy and the Investigator. Both parties will have an opportunity to review and comment on any manuscripts or abstracts arising from this study.

**Approval**	**Signature**	**Date**
Prof./Dr. ………..……..	________________________________<br>Investigator	____/____/2005___
Prof. Nick White	________________________________<br>Co-ordinating Investigator	____/____/2005___
Dr. Marco Corsi	________________________________<br>Sigma-Tau - Medical Department<br>Director	____/____/2005___
Dr. Antonella Bacchieri	________________________________<br>Sigma-Tau - Medical Department<br>Head of Biostatistics and Data Management	____/____/2005___

Signing this document I declare to have read the paragraph relevant to study acknowledgement and confidentiality and authorise Sigma-Tau i.f.r. S.p.A., Pomezia (Rome) - Italy to record my data on a computerised archive containing all the data pertinent to the study.

SYNOPSIS

Title	**A Phase III, randomized, non-inferiority trial, to assess the efficacy and safety of Dihydroartemisinin+Piperaquine (DHA+PPQ, Artekin) in comparison with Artesunate+Mefloquine (AS+MQ) in patients affected by acute, uncomplicated *Plasmodium falciparum* malaria.**
Protocol Number	ST3073-ST3074 DM040010
Phase	III
Methodology	A phase III, randomized, open label, two arms study.<br>Randomization will stratify infants, children and adults by age.
Study Duration	Each patient will be followed for 63 days.
Country	3 countries: Thailand, China and Laos
Study Centers	5 sites in Thailand, 1 site in China and 1 in Laos
Objectives	Primary objective:<br>The primary objective of the study is to measure the Day 63, PCR corrected cure rates of Artekin and AS+MQ and demonstrate that:<br>• the cure rate of Artekin is non-inferior to that of AS+MQ (non-inferiority margin = 5%);<br>• the cure rate of Artekin is at least 90%.<br>This cure rate is defined as the proportion of patients with adequate clinical and parasitological response at Day 63 plus those treatment failures identified as new infection by PCR.<br>Secondary objectives:<br>- The comparison of the uncorrected Day 63 cure rates of both drugs [(also known as Adequate Clinical and Parasitological Response (ACPR)].<br>- The comparison of the amount of overall treatment failure.<br>- The comparison of the safety profiles of the two treatments.<br>- Proportion of patients with Treatment Failure (TF).<br>- Proportion of aparasitaemic patients.<br>- Proportion of afebrile patients.<br>- Gametocytes carriage.<br>- Fractional change in haemoglobin/haematocrit.
Number of Subjects	1050 patients (700 DHA+PPQ; 350 AS+MQ).
Diagnosis and Main Inclusion Criteria	Males and Females aged between 3 months and 65 years inclusive, body weight at least 5 Kg, microscopically confirmed, monoinfection of Plasmodium *falciparum* or mixed infection, history of fever or presence of fever (tympanic temperature at ≥ 37.5 °C), written informed consent.
Study Product, Dose, Route, Regimen	Dihydroartemisinin+Piperaquine (DHA+PPQ, Artekin™).<br>Tablets containing 40 mg of Dihydroartemisinin and 320 mg of Piperaquine for adults patients or containing 20 mg of Dihydroartemisinin and 160 mg of Piperaquine for infants and children.
Duration of administration	Three days
Reference therapy	Artesunate+Mefloquine.<br>Tablets containing 50 mg of Artesunate and tablets containing 250 mg of Mefloquine

Criteria for efficacy evaluation	<u>Primary efficacy endpoint:</u><br>The primary efficacy endpoint will be the PCR-corrected adequate clinical and parasitological response (ACPR) at D63.<br><br><u>Secondary efficacy criteria</u>:<br>The secondary efficacy criteria will be:<br>- Crude or PCR uncorrected adequate clinical and parasitological response (PCR uncorrected ACPR).<br>The comparison of the uncorrected Day 63 cure rates of both drugs will be performed.<br>- Proportion of patients with Treatment Failure (TF).<br>TF is defined as:<br>• Failure to clear parasitaemia within the first 7 days.<br>• Patients with parasitaemia on any day after Day 7, irrespective of symptoms will be counted as TF and classified accordingly the following possibilities:<br>° Patients with resistant infection: recurrent parasitaemia and paired PCR results (matching paired genotypes).<br>° Patients with recurrent parasitaemia and ambiguous PCR results.<br>° Patients with recurrent parasitaemia and with missing PCR analysis (at pre- or post-dose).<br>° If PCR shows a different genotype they will be classified as new infections.<br>• Any Serious Adverse Event (SAE), which is considered by the Investigator as drug-related, and which results in withdrawal of study drug.<br>- Proportion of aparasitaemic patients.<br>The proportion of aparasitaemic patients will be evaluated on Days 1, 2 and 3.<br>- Proportion of afebrile patients.<br>The proportion of afebrile patients will be evaluated on Days 1, 2 and 3. Fever clearance may be calculated independently of relationship to parasite clearance.<br>- Gametocytes carriage.<br>The proportion of patients with gametocytes will be evaluated.<br>Gametocytes are the sexual or infective forms that are ingested by the mosquito that are responsible for transmission of malaria from one person to another one. Some drugs clear gametocytes and so they reduce transmissibility.<br>The presence of gametocytes on a blood slide is called gametocyte carriage.<br>- Fractional change in haemoglobin (Hb) and haematocrit.<br>The fractional change in haemoglobin/haematocrit will be evaluated weekly.
Criteria for safety evaluation	Adverse events, laboratory parameters, vital signs, ECG.

Statistical Methodology	The primary analysis will be based on a 97.5% (one-sided) Confidence Interval (CI) computed on the difference between the 63-day PCR corrected cure rates of the test and the reference treatments, respectively. In order to claim that DHA+PPQ is efficacious, the lower limit of the CI must be > -0.05 and the point estimate for the 63-day PCR corrected cure rate of DHA+PPQ must be > 0.90, in both the ITT and the Evaluable populations.
First patient in	April, 2005 (expected date)

TABLE OF CONTENTS

1 BACKGROUND INFORMATION 12
1.1 CURRENT CHALLENGES IN MALARIA CHEMOTHERAPY 12
1.2 NEW ANTIMALARIAL THERAPIES 13
1.2.1 Chlorproguanil–dapsone (CD) (LapDap) 13
1.2.2 Artemether –Lumefantrine (AL)-Coartemether (Riamet®, Coartem®) 14
1.2.3 Artesunate-amodiaquine and artesunate-sulfadoxine/pyrimethamine 14
1.2.4 Artesunate-pyronaridine 14
1.2.5 Drugs that are chemical alterations of well-established antimalarial drugs 15
1.2.6 Dihydroartemisinin-piperaquine (DHA+PPQ) (Artekin™) 15
1.3 RATIONALE 15
1.4 STUDY POPULATION 16
1.5 ETHICAL ASPECTS 16
2 TRIAL OBJECTIVES AND PURPOSE 17
2.1 PRIMARY OBJECTIVE 17
2.2 SECONDARY OBJECTIVES 17
3 TRIAL DESIGN 17
3.1 STUDY DESIGN 17
3.2 PRIMARY ENDPOINT 17
3.3 SECONDARY ENDPOINTS 18
3.4 SAMPLE SIZE 19
3.5 DURATION OF THE STUDY 20
3.6 FLOW-CHART 20
3.7 SELECTION OF THE PATIENTS 22
3.7.1 Inclusion Criteria 22
3.7.2 Exclusion Criteria 22
3.8 STUDY PROCEDURES 23
3.9 RANDOMISATION 26
3.10 TREATMENT ALLOCATION 26
3.11 STUDY MEDICATIONS 26
3.12 PACKAGING AND LABELLING 27
3.13 DOSAGE AND DOSING SCHEDULE 27
3.14 DRUG DISPENSING 27
4 CONCOMITANT THERAPIES 28
4.1 CONCOMITANT DRUG THERAPIES DISALLOWED 28
4.2 CONCOMITANT DRUG THERAPIES ALLOWED 28
4.3 SPECIAL CONDITIONS 28
4.4 RESCUE TREATMENTS 28
5 PATIENT WITHDRAWAL CRITERIA 28
6 SAFETY VARIABLES 28
6.1 ADVERSE EVENTS 28
6.1.1 Definition of an Adverse Event 28
6.1.2 Severity, Relationship of Event to Study Drug, and outcome 29
6.1.3 Defintion of a Serious Adverse Event 30
6.1.4 Reporting of adverse events 30
6.2 LABORATORY EVALUATIONS 31

6.3 VITAL SIGNS 31
7 CASE REPORT FORM (CRF) 31
8 STATISTICS 32
8.1 POPULATION ANALYSED 32
8.2 EFFICACY: PRIMARY ANALYSIS 32
8.3 EFFICACY: SECONDARY ANALYSIS 32
8.4 SAFETY AND TOLERABILITY 32
9 MONITORING AND QUALITY ASSURANCE 33
10 DATA MANAGEMENT 33
11 INVESTIGATOR RESPONSIBILITY 33
12 TRIAL REPORTS 34
12.1 STATISTICAL REPORT 34
12.2 FINAL INTEGRATED REPORT 34
13 ADMINISTRATIVE PROCEDURES 34
13.1 REGULATORY AUTHORITIES AND ETHICAL REVIEW COMMITTEE 34
13.2 INFORMED CONSENT 34
13.3 CONFIDENTIALITY AND PUBLICATION OF RESULTS 34
13.4 PROTOCOL AMENDMENTS 34
14 DETAILS ON STUDY SITES 34
15 STUDY COMMITTEES 35
15.1 STEERING COMMITTEE 35
15.2 DATA MONITORING BOARD 35
16 PROTOCOL VIOLATION 35
17 REFERENCES 35
18 APPENDICES 39

LIST OF ABBREVIATIONS

ACPR	:	Adequate Clinical and Parasitological Response
AE	:	Adverse Event
ALAT	:	ALanine AminoTransferase
AMMS	:	Academy of Military Medical Sciences
AS	:	Artesunate
ASAT	:	ASpartate AminoTransferase
β-HCG	:	Beta-Human Chorionic Gonadotropin
BP	:	Blood Pressure
CI	:	Confidence Interval
CITIC	:	China International Trust and Investment Corporation
CPMP	:	Committee for Proprietary Medicinal Products
CRF	:	Case Report Form
CRO	:	Contract Research Organization
DBP	:	Diastolic Blood Pressure
DHA	:	Dihydroartemisinin
ECG	:	Electrocardiogram
EDTA	:	Ethylenediaminetetraacetic Acid
ETF	:	Early Treatment Failure
FCT	:	Fever Clearance Time
GCP	:	Good Clinical Practice
GGT	:	Gamma-Glutamyl Transferase (Gamma-Glutamyl Transpeptidase)
GLP	:	Good Laboratory Practice
GMP	:	Good Manufacturing Practice
h	:	Hour
Hb	:	Haemoglobin
HR	:	Heart Rate
ICH	:	International Conference on Harmonization
IEC	:	Independent Ethics Committee
IRB	:	Institutional Review Board
ITT	:	Intention To Treat
IU	:	International Unit
IVR	:	Interactive Voice Response
KPF	:	Kunming Pharmaceutical Factory

LCF	:	Late Clinical Failure
LPF	:	Late Parasitological Failure
MMV	:	Medicines for Malaria Venture
MQ	:	Mefloquine
mg	:	Milligram
NA	:	Not Applicable
PCR	:	Polymerase Chain Reaction
PCT	:	Parasite Clearance Time
PP	:	Per Protocol
PPQ	:	Piperaquine
RBC	:	Red Blood Cells
R&D	:	Research and development
SAE	:	Serious Adverse Event
SAS	:	Statistical Analysis System
SBP	:	Systolic Blood Pressure
SP	:	Sulfadoxine Pyrimethamine
TDR	:	Tropical Disease Research
TF	:	Treatment Failure
URI	:	Upper Respiratory Infection
WBC	:	White Blood Cells
WHO	:	World Health Organization

1 BACKGROUND INFORMATION

1.1 Current challenges in malaria chemotherapy

The impact of malaria on the health and economic development of human populations is greatest in the tropics and sub-tropics. The number of currently available antimalarial drugs is few[1]. Those that are easily affordable by many malaria-endemic countries are restricted to chloroquine, sulfadoxine/pyrimethamine, quinine and amodiaquine. In Southeast Asia, mefloquine alone or in combination with artesunate tends to be used. Mefloquine+Artesunate is recommended in Thailand and Cambodia. Dihydroartemisinin+ piperaquine (CV8) is used in Vietnam. Sulphadoxine/pyrimethamine (SP) is used in Laos and Mefloquine+Artesunate in Myanmar. Artemisinin derivatives as monotherapy are increasingly used throughout the tropical world, although the World Health Organization recommends their use only with another antimalarial drug[2]. Today, we have several loose (non-fixed)-dose and one fixed-dose artemisinin-based combinations, artemether-lumefantrine.
Antimalarial drug resistance is a critical factor that undermines malaria control and hinders the achievement of the roll back malaria (RBM) targets. Equally important, on an individual level drug resistance accounts for rising mortality from malaria, *Plasmodium falciparum* resistance to chloroquine and sulfadoxine/pyrimethamine is now widespread. One factor in the emergence of drug-resistant *P. falciparum* has been the widespread use (sometimes over the counter) of two principal classes of antimalarial drugs; namely, the quinoline (e.g. chloroquine and amodiaquine) and antifolate (sulfadoxine/pyrimethamine) drugs; but the real problem was continuing to use these drugs after resistance had already emerged. Because the latter drugs are inexpensive, they have been used and recommended even in the face of poor efficacy. In addition, access to good diagnosis and treatment is low in most Asia settings. Moreover, poverty and the inability to travel to a local clinic has often resulted in the purchase of sub-therapeutic doses of chloroquine in many parts of rural Asia and treatment at the home. Such widespread drug use leads to sub-therapeutic drug concentrations in the plasma and causes a potent selective pressure for the development of resistant parasites[3]. Overcoming or reducing resistance requires the adoption of several strategies; central to these is the use of effective chemotherapy for those who need it. In addition, there is need for the development of new molecules and to implement strategies to protect drugs against the rapid emergence of resistance. Resistance to single-drug therapies will inevitably occur. Consequently, drug combinations targeting different metabolic pathways, a standard concept for viral and bacterial diseases, are now being adopted for the treatment of malaria as well.

The artemisinin derivatives in combination with standard antimalarials are presently the best therapeutic option for treating drug-resistant malaria and retarding the development of resistance[3, 4, 5]. The artemisinin derivatives are the most rapidly-acting and efficacious antimalarial drugs[6]. Their pharmacodynamic properties such as rapid absorption and activity against many stages of the malaria life cycle, including young asexual forms (rings) and early sexual forms (gametocytes[7]), make them an ideal choice. Their short elimination half-lives (<1 hour for artesunate), protects them from resistance. Furthermore, they reduce gametocyte carriage and infectivity and have been the main reason why transmission has been reduced on the Thai–Myanmar border[8, 9, 10]. Tolerability of these drugs is also very good[11]. Data on their safety during pregnancy are limited but encouraging[12], but they are not generally recommended during the first trimester unless better alternatives are unavailable or unsuitable.

Over the last 40-50 years, new antimalarial drugs have been developed slowly, but at present prospects are brighter. Prior to the Second World War, quinine, pamaquine, chloroquine and mepacrine were developed. These were followed by proguanil and amodiaquine in the 1940s; primaquine and pyrimethamine in the 1950s; S/P in the 1960s; artemisinin in the 1970s; in China and several drugs in the 1980s such as mefloquine, halofantrine and various Chinese compounds – pyronaridine, piperaquine and the artemisinin derivatives (artemether and artesunate). More recently dihydroartemisinin has been developed. However, the use of the Chinese compounds has been

confined almost exclusively to China and a few Asian countries. Only recently have the artemisinin derivatives been adopted more widely. In the past decade, three new antimalarial drugs have been registered in Western countries and made available on the open market: atovaquone–proguanil (for uncomplicated malaria treatment and prophylaxis in travellers), injectable artemether (artemotil) for the treatment of severe malaria and artemether–lumefantrine (coartem) for the treatment of uncomplicated malaria.

Despite these developments, there are presently few affordable and efficacious antimalarial drugs for the resource constrained countries in Africa. The vast majority of malaria cases are treated with cheap but largely ineffective drugs (chloroquine or sulfadoxine/pyrimethamine). The current R&D model is largely based (with few exceptions) on public sector basic research and discovery of products, which are then further developed by the private sector. However, the latter model has been ineffective in providing new antimalarial drugs. Only four of approximately 1400 drugs registered worldwide during 1975–1999 were antimalarials[13]. The distribution of Artemisinin and its derivatives is still limited because their development does not meet international criteria. The rules and criteria regulating drug development have changed significantly in the recent past and stricter criteria are being applied currently than in the past, particularly after the adoption of the guidelines of the International Conference for Harmonisation guidelines. As a consequence, developing a new drug is now more expensive and takes longer. The latter, along with the low solvency of the markets explains in part the low output of new antimalarial drugs by the Western, research-based pharmaceutical industry. The world's drug market is highly skewed; more than 80% of the US$ 337 billion market in 1999 was in the USA, Europe and Japan, which account for less than 20% of the world's population. Traditionally, Western countries promoted antimalarial drug research during the colonial era, and in case of war in endemic countries (e.g. mefloquine during the Vietnam war). Today, none of these conditions exist, yet the traveller market alone is insufficient. Nonetheless, a number of drugs and drug combinations are in various stages of development, the majority of which will be for the treatment of uncomplicated falciparum malaria.

1.2 New antimalarial therapies

The majority of new therapies are combinations of existing antimalarial drugs with an artemisinin derivative.

1.2.1 Chlorproguanil–dapsone (CD) (LapDap)

This is a fixed-dose antifolate combination that has been developed jointly by GlaxoSmithKline and WHO/TDR (Tropical Disease Research). CD received approval from the UK Regulatory Agency in July 2003, and GlaxoSmithKline and WHO are collaborating on operational research on public health and access issues of CD in resource-poor settings, ahead of its anticipated introduction. The two components in CD have been in use singly for several years and they were combined in order to obtain an antifolate combination with shorter elimination time than sulfadoxine/pyrimethamine. In addition the combination could lower the probability of selecting resistant parasites. CD compares well with sulfadoxine/pyrimethamine[14] in controlled studies in East Africa and is effective in patients who have failed on sulfadoxine/pyrimethamine[15]. However, there is some debate regarding its optimal role in treating falciparum malaria in Africa because it shares similar genetic mechanisms for resistance as sulfadoxine/pyrimethamine. Consequently its therapeutic life span might be short, an observation that might compromise the use of LapDap monotherapy and in combination with artesunate (chlorproguanil–dapsone–artesunate). Chlorproguanil–dapsone–artesunate (CDA) is at an early phase of development, with funding from the Medicines for Malaria Venture (MMV), GlaxoSmithKline and WHO/TDR, having completed pre-clinical toxicological studies and phase II clinical trials are near completion.

1.2.2 Artemether –Lumefantrine (AL)-Coartemether (Riamet®, Coartem®)

This is a fixed-dose combination of artemether (an artemisinin) and lumefantrine (a longer-resident drug also referred to as benflumetol). The registered indications and branding for this drug cover treatment, including standby treatment, of uncomplicated malarial episodes caused by pure or mixed *Plasmodium falciparum* infections. The combination is expected to confer mutual protection against resistance and prevent recrudescence after artemether therapy. The components of this combination were originally studied and developed in China by the Academy of Military Medical Sciences (AMMS), Beijing and Kunming Pharmaceutical Factory (KPF), Kunming. The combination product has been registered in China since 1992 but underwent further development when Novartis signed a collaborative agreement in 1994 with AMMS, KPF and CITITEC, the technology arm of the China International Trust and Investment Corporation (CITIC). Studies for international registration started in 1995. The drug was registered in Switzerland in 1999 and has since received marketing authorisation in several endemic and non-endemic countries. Coartemether is marketed under a dual-branding, dual-pricing strategy. Riamet® (six doses over either 3 days or 5 days) is available in developed, non-endemic countries as a standby treatment for travellers at a price comparable with the latest antimalarial introductions. Coartem® is registered and marketed in malaria-endemic countries as either a four (no longer recommended)- or six-dose treatment of uncomplicated falciparum malaria at prices comparable with locally available products. Recently, an agreement was reached between Novartis and WHO for the drug to be made available to the public sector of developing countries at a preferential price. Few clinical trials have been conducted both with the original Chinese combination product and the subsequent product for international registration, mostly with the four-dose regimen. The six-dose regimen was equivalent to mefloquine-artesunate but was reported to be better tolerated [17, 18]. Work between Novartis and WHO has led to a more user-friendly packaging of the six-dose treatment for improved adherence, which is now being field tested. Paediatric formulation is also being developed. Trials have been conducted for regulatory submission to extend the label to cover children who weigh less than 10 kg.

1.2.3 Artesunate-amodiaquine and artesunate-sulfadoxine/pyrimethamine

Several clinical trials involving the latter drugs have been completed in Africa, while Artesunate-mefloquine has been widely used in Thailand and other parts of Southeast Asia as a loose formulation, and has been proven to be highly efficacious in areas of mefloquine resistance.
The addition of artesunate to mefloquine and the splitting of the mefloquine dose have shown to improve the absorption and tolerability of mefloquine. In large studies in Thailand the combination was shown to reduce transmission of falciparum malaria while the sensitivity to mefloquine was preserved. Artesunate-mefloquine is now the first line treatment for uncomplicated malaria in several countries in Southeast Asia. Artesunate-amodiaquine and artesunate-mefloquine will be further developed as fixed-dose combinations by WHO/TDR (Drugs for Neglected Diseases/populations) and will undergo all the relevant pre-clinical and clinical studies before registration. In addition, a blister pack of artesunate-sulfadoxine/pyrimethamine and artesunate-amodiaquine have been developed using age-based dosing and is being used in clinical trials in Africa.

1.2.4 Artesunate-pyronaridine

Artesunate-pyronaridine builds on the rationale of using an artemisinin derivative with a longer-acting partner drug. **Pyronaridine** is an antimalarial compound that has been synthesized and developed in China, where it obtained marketing authorization for the treatment of malaria in the 1980s. It has proven efficacy against drug-resistant falciparum malaria in Africa[19]. The efficacy in Thailand of the polybioavailable formulation against multidrug-resistant malaria was less than 90% [20]. In the same setting, a better formulation was >95% efficacious[21].

1.2.5 Drugs that are chemical alterations of well-established antimalarial drugs

Work is being planned for drugs that are chemical alterations of well-established antimalarial drugs. **Artemisone** is a metabolically stable semi-synthetic derivative of artemisinin that is being developed by Bayer & MMV. Several trioxanes obtained by total synthesis are now available and are being assessed for further development by MMV. **Isoquine** is an isomeric derivative of amodiaquine that might not generate the toxic quinone-imine metabolites that are thought to have a role in the development of neutropenia and hepatitis. The development of the latter drug is also being coordinated by MMV. Short-chain chloroquine analogues with better efficacy on chloroquine-resistant isolates are being researched at Tulane University. A novel compound, **fosmidomycin**, has recently been tested in small numbers of patients. Fosmidomycin inhibits 1-deoxy-D-xylulose 5-phosphate reductoisomerase, an enzyme of the nonmevalonate pathway of isoprenoid biosynthesis, which is absent in humans but present in many pathogens and plants. Fosmidomycin was well tolerated and produced modest cure rates[22, 23]. However, it is a drug worthy of more trials and could be used in combination with other antimalarials.

1.2.6 Dihydroartemisinin-piperaquine (DHA+PPQ) (Artekin™)

Artekin™ was developed in China and is registered in China and Cambodia. It has been evaluated extensively in clinical trials in Thailand, Vietnam, Cambodia and China[24]. Efficacy has been high and tolerability uniformly excellent in all trials in these multidrug-resistant areas including Hai Nan, China, where piperaquine-resistance was common after extensive use of mass prophylaxis. Initially, the co-formulation also included primaquine and trimethoprim (CV8). This product is still part of national policy in Vietnam. Piperaquine is an orally active bisquinoline discovered by Rhône-Poulenc in the early 1960s and developed for clinical use in China in 1973. Piperaquine is approximately equivalent to chloroquine against sensitive parasites but is significantly more effective than chloroquine against resistant *P. falciparum*. Piperaquine replaced chloroquine as the recommended treatment for falciparum malaria in China in 1978. Overall, 194 140 kg of piperaquine phosphate, equivalent to 140 000 000 adult doses, were used for mass prophylaxis and treatment. Surveillance at the time found no adverse events other than rare cases of a rash. Piperaquine is well known for its association with gastrointestinal disturbance. Dihydroartemisinin is the active metabolite of artesunate and artemether. It has equivalent clinical efficacy to the more widely used artesunate. A development programme has been developed between Holleykin Pharmaceuticals and Guangzhou University (China), The University of Oxford, MMV, and Sigma-Tau Industrie Farmaceutiche Riunite SpA to support the international registration of the drug.

1.3 Rationale

The fight against malaria, which the WHO reactivated in 1999 with its Roll Back Malaria programme, emphasizes early curative treatment of malaria, particularly in children, in order to decrease mortality and morbidity [31]. Recent assessments confirm a disturbing persistence of endemic malaria with an estimated 400 million cases and 1.5–2.7 million deaths per year. In sub-Saharan Africa alone, *Plasmodium falciparum* is responsible for approximately 220 million cases and one million deaths per year, 75% of them being children under 5 years old.

The drug resistance of *P. falciparum* to CQ was first reported in the 1960s. Resistance to SP was reported later in the 1980s, and has now spread globally. After several centuries of use, natural quinine is still effective against *P. falciparum* everywhere in the world except in Southeast Asia and South America, where decreased susceptibility is reported. No clinical resistance to the artemisinin derivatives has been observed, despite their use for 15 years in Asia and, more recently in Africa. Nonetheless, the current state of parasite resistance to other antimalarial drugs worldwide is worrying, hence the need to develop new therapeutic options for the treatment of *P. falciparum* malaria by simultaneously reassessing old and forgotten drugs, while searching for new drugs, and identifying synergistic drug combinations.

The elimination half-life of antimalarial drugs is an important factor with respect to erythrocytic schizonticidal action (the killing of parasitized red cells with asexual forms) because sufficiently high drug concentrations must be maintained for at least three life cycles, i.e., 6 days. Therefore, treatment with drugs that have a short half-life of less than 1 day must be continued for 6–7 days if given as monotherapy. Compounds with a long half-life, i.e., greater than 5 days, have the advantage of allowing treatment for 1–3 days. Conversely, however, antimalarial drugs with a long half-life allow sub-inhibitory concentrations to remain in the blood and this promotes the selection of resistant strains.
Presently, there are two philosophies that guide the selection of drug combinations: 1) combining two drugs with similar and preferably short (chlorproguanil and dapsone) or intermediate (sulfadoxine and pyrimethamine) half-lives; 2) combining two drugs with different but complementary, half-lives; one short and the other long, which leads to a risk of de facto monotherapy when the shorter half life product is eliminated, a risk that could be minimized by choosing a compound that is rapidly effective on 95% of the parasite population such as the artemisinin derivatives. The partner drug with the longer half-life such as mefloquine, lumefantrine, amodiaquine or piperaquine has a smaller parasite biomass to clear and hence a low risk for the selection of resistant parasites.

Moreover, compliance is another important factor to take into consideration when developing a new combination. DHA+PPQ is considered a highly efficacious and safe combination that is likely to have a positive risk/benefit ratio in malaria therapy. Artekin™ is a second generation Artemisinin-based Combination Therapy (ACT) with similar efficacy to that of Coartem (artemether+lumefantrine) or Artesunate+Mefloquine but with a simpler dosing scheme that will aid better compliance. Moreover, its good safety profile and affordable cost make it ideal for resource constrained countries. Most of the clinical trials evaluating Artekin™ have been conducted without the sponsorship of a pharmaceutical company. In addition, the formulation of DHA+PPQ used in some trials[24, 25, 26, 27, 28, 29], though compliant with Chinese GMPs, was not compliant with the GMP standards required by the European or US Health Authorities. For the latter reasons Sigma-Tau has used the information/data generated from previous trials to support the design of a new Phase III development strategy using Artekin produced according to good GMP standards.

1.4 Study Population

Patients selected for this trial will be adult and paediatric patients of both sexes, affected by acute, uncomplicated falciparum malaria.

1.5 Ethical Aspects

Before the start of the study, the study protocol will be submitted to the relevant Independent Ethical Committee (IEC) or Institutional Review Board (IRB) for approval. The EEC/IRB's written approval of the study shall be appended to the standard study documents at the sponsor and at the location of the investigator.
All patients will receive a full explanation of the proposed study with the potential risks and benefits from the Investigator or one of the medical research personnel specifically designated by the Investigator. Prior to entering the study, written informed consent will be obtained and a copy of it will be provided to the patient (see Appendix IV).
Patients are free to withdraw from the study at any time for any reason. Their decision to discontinue participation will in no way jeopardise the subsequent availability of health care at the Investigational Centre.
The Investigator declares by signing this protocol that he/she will perform the study in compliance with the protocol, scrupulously following the Good Clinical Practice (GCP) and all the applicable regulatory requirements.

2 TRIAL OBJECTIVES AND PURPOSE

The aim of this study is to show that a new formulation of DHA+PPQ is as good as (not inferior to) artesunate combined with mefloquine (AS+MQ) for the treatment of acute uncomplicated falciparum malaria in non pregnant patients of all ages. Safety and tolerability will also be assessed.

2.1 Primary objective

The primary objective of the study is to measure the Day 63, PCR corrected cure rates of Artekin and AS+MQ and demonstrate that:

- the cure rate of Artekin is non-inferior to that of AS+MQ (non-inferiority margin = 5%);
- the cure rate of Artekin is at least 90%.

This cure rate is defined as the proportion of patients with adequate clinical and parasitological response at Day 63 plus those treatment failures identified as new infection by PCR.

2.2 Secondary objectives

The secondary objectives of the study will be the between treatment comparison of the :

- uncorrected Day 63 cure rates of both drugs;
- safety profiles of the two treatments;
- proportion of patients with Treatment Failure (TF);
- proportion of aparasitaemic patients;
- proportion of afebrile patients;
- gametocytes carriage;
- fractional change in haemoglobin/haematocrit.

3 TRIAL DESIGN

3.1 Study Design

This is a phase III, randomized, open label, two arms study.
Investigational centres will be involved in the enrolment of 1050 patients (700 DHA+PPQ; 350 AS+MQ).
The main justification for not performing a double-blind study is the requiremnt to use the double-dummy technique that, in turn, would require a placebo for DHA+PPQ and a placebo for AS+MQ, and a treatment schedule which would be very heavy for the patient.
In order to ensure concealmemt of treatment allocation and avoid other biases, the following requirements will be assured:

1 the randomization will be under blind conditions and treatment allocation will be concealed until the final recruitment of the patient (see section 3.9);
2 the PCR reading will be blinded/masked (see section 3.8);
3 an independent Data Monitoring Board will review a significant percentage of efficacy data and all safety data (see section 15.2).

3.2 Primary Endpoint

The primary endpoint will be the PCR-corrected adequate clinical and parasitological response (PCR corrected ACPR) at D63.
ACPR is defined as the absence of parasitaemia on D63 irrespective of the tympanic temperature and not meeting any of the criteria of early treatment failure or late clinical or parasitological failure.

Patients classified as failures by clinical and parasitological criteria will be considered ACPR if the PCR analysis will show a new infection rather than a recrudescence.

The total treatment failure is defined according to the WHO criteria (WHO 2003) as the sum of early* and late** treatment failures.

* Early Treatment Failure (ETF)
(i) development of danger signs or severe malaria on Days 0, 1, 2 or 3, and the presence of parasitaemia,
(ii) parasitaemia on Day 2 > Day 0 count irrespective of axillary temperature,
(ii) parasitaemia on Day 3 with fever (temperature ≥ 37.5°C),
(iv) parasitaemia on Day 3 ≥ 25 % of count on Day 0.

** Late treatment failure (LTF)
Late Clinical Failure (LCF):
(i) development of danger signs or severe malaria after Day 3 in the presence of parasitaemia (See Appendix V for the critera for severe malaria/danger signs),
(ii) presence of parasitaemia and temperature ≥ 37.5°C (or history of fever) on any day from Day 4 to Day 63, without previously meeting the criteria of ETF.

Late Parasitological Failure (LPF):
Reappearance of parasitaemia after initial clearance between Day 7 and Day 63 and temperature < 37.5°C, without previously meeting the criteria of ETF or LCF.

3.3 Secondary Endpoints

The secondary endpoints will be:

- Crude or PCR uncorrected adequate clinical and parasitological response (PCR uncorrected ACPR).

The comparison of the uncorrected Day 63 cure rates of both drugs will be performed.

- Proportion of patients with Treatment Failure (TF).

TF is defined as:

- Failure to clear parasitaemia within the first 7 days.
- Patients with parasitaemia on any day after Day 7, irrespective of symptoms will be counted as TF and classified according to the following possibilities:
  ° Patients with resistant infection: recurrent parasitaemia and paired PCR results (matching paired genotypes).
  ° Patients with recurrent parasitaemia and ambiguous PCR results.
  ° Patients with recurrent parasitaemia and with missing PCR analysis (at pre- or post-dose).
  ° If PCR shows a different genotype they will be classified as new infections.
- Any Serious Adverse Event (SAE), which is considered by the Investigator as drug-related, and which results in withdrawal of study drug.

- Proportion of aparasitaemic patients.

The proportion of aparasitaemic patients will be evaluated on Days 1, 2 and 3.

- Proportion of afebrile patients.

The proportion of afebrile patients will be evaluated on Days 1, 2 and 3.
Fever clearance may be calculated independently of relationship to parasite clearance.

- Gametocytes carriage.

The proportion of patients with gametocytes will be evaluated.
Gametocytes are the sexual or infective forms that are ingested by the mosquito that are responsible for transmission of malaria from one person to another. Some drugs clear gametocytes and so they reduce transmissibility.
The presence of gametocytes on a blood slide is called gametocyte carriage.

- Fractional change in haemoglobin (Hb) and haematocrit.

The fractional change in haemoglobin/haematocrit will be evaluated weekly.

- Safety profiles of the two treatments

Adverse events, vital signs, blood chemistry and haematology, electrocardiogram (ECG).

3.4 Sample Size

The study is designed as a non-inferiority trial.
The level of the PCR-corrected cure rate at day 63 for Artesunate+Mefloquine has been estimated, through a literature search, to be in the range 92%-95%.
The opinion of physicians and experts in malaria treatment has been sought for defining the non-inferiority margin, and this has been set at 5%. In addition, the experts have agreed that, in order for the new treatment to be considered efficacious in Asia, its PCR-corrected cure rate at day 63 should not be lower than 90%. Therefore, in the present trial, DHA+PPQ will be considered efficacious if both the following conditions are met (see section 2.1):

- (i) the PCR-corrected cure rate at day 63 of DHA+PPQ is not inferior to the cure rate of AS+MQ by 5% or more, and independently of the reference drug performance,
- (ii) the cure rate of DHA+PPQ is at least 90%.

In summary, for the non-inferiority analysis, the clinical and statistical specifications for the sample size computations are as follows:

- Primary endpoint = PCR-corrected cure rate at day 63.
- Primary analysis = based on 97.5% one-sided confidence intervals for the difference in cure rates.
- Alpha = 0.025 (one-sided).
- Power = 80%.
- Cure rate of Artesunate+Mefloquine = at least 92%.
- Non-inferiority margin for the difference (test-reference) = -0.05.
- Randomization: based on a 2 :1 allocation scheme (test : reference).
- Rate of patient attrition in the Evaluable Population (i.e. rate of withdrawals and protocol violations) = 15%.
- Primary Analysis Populations = both the Intention To Treat (ITT) and the Evaluable Populations.

A simulation was performed for estimating the overall power that this study would have for addressing both of its primary objectives, i.e. power was computed for both the lower bound of a 97.5 one-sided confidence interval for the treatment difference being above –0.05 and the point estimate for the test treatment being at least 90%. The scenario in which both treatments have cure rates of 90% has the contra-indicating limitation of a power of 50% for the point estimate for the test treatment to exceed 0.90, no matter how large the sample size is (in fact, if the true success rate is 90% for the test treatment, then 50% of studies will have an observed success rate > 90% and 50% of studies will have an observed success rate < 90%). Thus, one inherently needs to make the assumption that both cure rates are at least 91% in order to identify a realistic sample size with acceptable power.
Details on the above mentioned simulation are provided in a separate document (see Sample Size and Design Justification for The Pivotal Clinical Trials of Dihydroartemisinin+ Piperaquine**)**.
Based on the above assumptions, the recommended sample size is 350 in the Artesunate+Mefloquine arm and 700 in the Dihydroartemisinin+Piperaquine arm (1050 patients in total).
The sample size of 1050 patients will concern the ITT population. For both the test and the reference compounds, the cure rates are expected to be higher in the Evaluable population as compared to the ITT population. Therefore, even if the Evaluable population is smaller than the ITT population, no impact on sample size is expected.
An unequal randomization with 2:1 structure was chosen because it assures a larger sample size for the DHA+PPQ group which provides the following advantages:

- a more precise estimate of the DHA+PPQ cure rate;
- a bigger sample for the integrated safety data base of DHA+PPQ, that renders more likely the elucidation of rare adverse reactions, if any.

From an ethical point of view, this choice appears reasonable in view of the previous data on DHA+PPQ, in terms of both safety and efficacy.

3.5 Duration of the Study

Each patient will be followed for 63 days.

3.6 Flow-Chart

As part of routine practice in outpatient care facilities, all febrile patients have a thick blood smear for malaria before antimalarial treatment is administered. Patients attending the study health facility who have a positive blood smear for *P. falciparum* will be informed by the health facility staff about the malaria clinical trial and they will be requested to freely consent to partcipate in the trial. Those who decline to participate will receive the standard treatment for uncomplicated falciparum malaria. Patients who accept to paticipate in the study will receive detailed explanations about the trial from the study staff. Specifically they will be informed that two antimalarial drugs are being tested and the option given to the patient will be decided randomly (by chance). They will be asked to sign the informed consent form (see Appendix VI for enrollment).
At enrolment, patients will be assigned a sequential study number. Patients will be managed as outpatients and treatment doses will be given under direct medical supervision.
Patients will be encouraged to return to the clinic for follow up assessment on days 1, 2, 3, 7, 14, 21, 28, 35, 42, 49, 56, 63 and any unscheduled day that they feel ill.

Patients will be assessed as summarized in the following flow-chart.

Figure 1: Flow-chart

	Day 0 (pre-dose)	Day 1	Day 2	Day 3	Day 7	Day 14	Day 21	Day 28	Day 35	Day 42	Day 49	Day 56	Day 63	Day of any recurrent parasitemia
Visits	***V1***	***V2***	***V3***	***V4(4)***	***V5***	***V6***	***V7***	***V8***	***V9***	***V10***	***V11***	***V12***	***V13***	
**Demographic data/Medical history**	x													
**recording**	x													
**examination**	x	x	x	x	x	x	x	x	x	x	x	x	x	x
**Vital signs and weight(1)**	x(1)	x	x	x	x	x	x	x	x	x	x	x	x	x
**Blood smear(3) (thick and thin)**	x	x	x	x	x	x	x	x	x	x	x	x	x	x
**Electrocardiogram (ECG)**	x		x					x					x	x
**Pregnancy test**	x													
	x				x	x	x	x	x	x	x	x	x	x
**Hematology/biochemistry**	x							x					x(2)	x
**Urinalysis**	x							x					x(2)	
**Adverse events recording**	x	x	x	x	x	x	x	x	x	x	x	x	x	x
**PCR-sample**	x													x
	x	x	x	x	x	x	x	x	x	x	x	x	x	x
**Study medications**	x	x	x											

1. *Weight will be measured at D0*
2. *Only if abnormal at D28*
3. *Daily until negative for asexual forms of parasite*
4. *Omit visit 4 if smear negative on visit 3 (day 2)*

3.7 Selection of the Patients

3.7.1 Inclusion Criteria

In order to be eligible to enter the protocol, patients must meet the following criteria:

1. Males and Females aged between 3 months and 65 years inclusive.

2. Body weight at least 5 Kg.

3. Microscopically confirmed, monoinfection of *Plasmodium falciparum* (asexual forms parasitaemia ≥ 5/500 WBC or mixed infection).

4. History of fever or presence of fever (tympanic temperature at ≥ 37.5 °C).

5. Informed consent obtained by patients and in the case of children, by parents or guardians.

6. Willingness and ability to comply with the study protocol for the duration of the trial.

3.7.2 Exclusion Criteria

Patients will not be admitted to the study if they meet any of the following exclusion criteria:

1. Participation in any investigational drug study during the previous 30 days.

2. Known hypersensitivity to the study drugs.

3. Severe malaria.

4. Presence of intercurrent illness or any condition which in the judgement of the investigator would place the subject at undue risk or interfere with the results of the study. Particular attention should be taken in excluding patients with liver or renal diseases.

5. Pregnant or lactating women. Urine test for β-HCG to be performed on any woman of child bearing age unless menstruating.

6. Mefloquine treatment in the previous 60 days.

7. Artekin treatment in the previous 3 months.

8. Parasitaemia of *P.falciparum* trophozoites > 40/1000 RBC.

3.8 Study procedures

Visit 1 (Day 0, pre-dose): screening visit/ administration of the study drug

1. Informed Consent
   A signed informed consent from the patient or parent/guardian must be obtained before any tests or evaluations related to the study eligibility are carried out.

2. Demographic Data and Medical History
   Demographic data and a general history of past and present illnesses will be collected.

3. Physical and Clinical Examination
   A general physical examination will be performed (see Table B of Appendix I). Vital signs (Systolic and Diastolic Blood Pressure, Heart Rate) and Weight will be also collected.
   A clinical examination will be performed (see Appendix I): symptoms, hearing, aural temperature (electronic thermometer).

4. Vital Signs and Weight
   Vital signs (Systolic and Diastolic Blood Pressure, Heart Rate) and Weight will be collected.

5. PCR
   A blood sample on a filter paper Whatman 3 M to genotype the infecting malaria parasites will be collected at baseline and at time of reappearance. PCR will be done for all cases of recurrent parasitaemia.

6. Blood Slide
   A thick and thin blood smear will be obtained from the subject to verify the presence of P. *falciparum* and to calculate the parasite density. Thick and thin blood films will be prepared and stained with Giemsa stain.
   Parasite density will be calculated by counting the number of asexual parasites per 500 leukocytes in the thick blood film, based on an assumed WBC of 8,000 /µl. The parasite density per microliter will be calculated using the following formula:

   $$\text{Parasite density} / \mu l = \frac{\text{Number of parasites counted x 8,000 (or WBC)}}{\text{Number of leukocytes counted}}$$

   Or per 1000 RBC on the thin film for higher parasitaemias.
   Parasite density then = No. PF trophozoites per 1000 RBC x Hct x 125.6

7. Laboratory Tests
   Blood haemoglobin, haematocrit, total white blood cell count, differential count.
   Total bilirubin, direct bilirubin, alkaline phosphatase, ASAT, ALAT, GGT, urea nitrogen, creatinine, total proteins, albumin.
   Urinalysis (Glucose, proteins, haemoglobin, ketones, bilirubin).
   The aim is for all patients to have these tests. However if parents of very young children refuse they may still be included in the study.

8. Electrocardiogram (ECG)
   A 12 lead ECG will be performed.
   QT/QTc interval prolongation will be evaluated.

9. Inclusion/Exclusion Checklist
   All inclusion and exclusion criteria must be met before enrolment in the study.

10. Administration of the study drugs

11. Pregnancy test
    In women of child bearing age (unless menstruating), a urine test for β-HCG will be performed.

Visit 2 (Day 1): *Open Label Treatment Period*)

During this visit, the following tests and evaluations will be performed:

1. Physical and Clinical Examination, Vital Signs
   A general physical examination and a clinical examination will be performed: symptoms, aural temperature (electronic thermometer).

2. Vital Signs
   Vital signs (Systolic and Diastolic Blood Pressure, Heart Rate) will be collected.

3. Blood Slide
   A thick and thin blood smear will be obtained from the subject to verify the presence of *P. falciparum* and to calculate the parasite density.

4. Concomitant Pharmacological Treatments
   Concomitant medications being taken by the patients will be recorded. For a list of allowed and disallowed medications, see section 4.

5. Adverse Event Report
   The appearance or absence of adverse events from the previous visit and during the visit will be recorded.

6. Administration of the study drugs

Visit 3 (Day 2): *Open Label Treatment Period*)

1. Physical and Clinical Examination, Vital Signs
   A general physical examination and a clinical examination will be performed: symptoms, aural temperature (electronic thermometer).

2. Vital Signs
   Vital signs (Systolic and Diastolic Blood Pressure, Heart Rate) will be collected.

3. Blood Slide
   A thick and thin blood smear will be obtained from the subject to verify the presence of *P. falciparum* and to calculate the parasite density.

4. Concomitant Pharmacological Treatments
   Concomitant medications being taken by the patients will be recorded. For a list of allowed and disallowed medications, see section 4.

5. ECG

A 12 lead ECG will be performed.
QT/QTc interval prolongation will be evaluated.

6. Adverse Event Report
The appearance or absence of adverse events from the previous visit and during the visit will be recorded.

7. Administration of the study drugs

Visit 4 (Day 3): *Open Label Treatment Period*)

1. Physical and Clinical Examination, Vital Signs
A general physical examination and a clinical examination will be performed: symptoms, aural temperature (electronic thermometer).

2. Vital Signs
Vital signs (Systolic and Diastolic Blood Pressure, Heart Rate) will be collected.

3. Blood Slide
A thick and thin blood smear will be obtained from the subject to verify the presence of *P. falciparum* and to calculate the parasite density.

4. Concomitant Pharmacological Treatments
Concomitant medications being taken by the patients will be recorded. For a list of allowed and disallowed medications, see section 4.

5. Adverse Event Report
The appearance or absence of adverse events from the previous visit and during the visit will be recorded.

Visits 5, 6, 7 (Days 7, 14, 21)): *Open Label Treatment Period*)

During these visits, the same procedures as the visit 4 will be applied.

Moreover, gametocyte prevalence will be evaluated at D7, D14, D21.

Visit 8 (Day 28): *Open Label Treatment Period*)

1. Physical and Clinical Examination
A general physical examination and a clinical examination will be performed (see Appendix I): symptoms, aural temperature (electronic thermometer).

2. Vital Signs
Vital signs (Systolic and Diastolic Blood Pressure, Heart Rate) will be recorded.

3. Blood Slide
A thick and thin blood smear will be obtained from the subject to verify the presence of *P. falciparum* and to calculate the parasite density. Thick and thin blood films will be prepared and stained with Giemsa stain.
Parasite density will be calculated by counting the number of asexual parasites per 500 leukocytes in the thick blood film, based on an assumed WBC of 8,000 /µl.
Gametocyte prevalence will also be evaluated.

4. Laboratory Tests
   Blood haemoglobin, haematocrit, total white blood cell count, differential count.
   Total bilirubin, direct bilirubin, alkaline phosphatase, ASAT, ALAT, GGT, urea nitrogen, creatinine, glucose, total proteins, albumin.
   Urinalysis (Glucose, proteins, haemoglobin, ketones, bilirubin).

5. Concomitant Pharmacological Treatments
   Concomitant medications being taken by the patients will be recorded. For a list of allowed and disallowed medications, see section 4.

6. Electrocardiogram (ECG)
   A 12 lead ECG will be performed.
   QT/QTc interval prolongation will be evaluated.

Visits 9, 10, 11, 12 (Days 35, 42, 49, 56)

During these visits, the same procedures as the visit 5 will be applied.

Visit 13 (Days 63)

During this visit, the same procedures as the visit 8 will be applied.

The PCR readings will be carried out in a blinded manner, i.e. by personnel different from the treating physician/investigator. In addition, a centralized and independent double-check of a significant percentage of parasitological slides and PCR films will be carried out by the members of the Data Monitoring Board (see section 15.2). The percentages of slides and films to be reviewed and the corresponding statistical justifications will be specified in the Statistical Analyis Plan.

3.9 Randomisation

Patients will be randomly assigned to one of the two treatment groups (DHA+PPQ or AS+MQ). Blinding in the randomization process will be attained by the use of sealed envelopes. A procedure for guiding to the correct use of the sealed envelopes will be put in place.

Complete blocks of treatment materials will be sent to the investigation centers.

An unequal randomization, 2:1 (Artekin:AS+MQ), will be used to provide more precise estimates of the DHA+PPQ cure rates, as well as to provide more patients for the integrated safety data base of the combination therapy under evaluation.

The randomization list will be generated by MDS, using the plan procedure of SAS.

3.10 Treatment Allocation

Treatment allocation and administration of medications will be performed by the Investigator or the study nurse.
To allocate patients to the appropriate treatment group, the study nurse will select the next available treatment number and corresponding study regimen. The study nurse will record the data and time of treatment assignment and the patient's study number.

3.11 Study Medications

The medications to be evaluated in this study will be prepared by Sigma-Tau i.f.r. S.p.A., Pomezia (Rome), Italy.

Dihydroartemisinin (DHA) and Piperaquine (PPQ) = Artekin (Sigma-Tau)

Medication will consist of tablets containing 40 mg of Dihydroartemisinin and 320 mg of Piperaquine (for adult patients) or tablets containing 20 mg of Dihydroartemisinin and 160 mg of Piperaquine (for infants and children).

Artesunate (AS) and Mefloquine (MQ)

Medication will consist of tablets containing 50 mg of Artesunate (Arsumax, Sanofi-Synthélabo) and tablets of 250 mg of Mefloquine (Lariam, Roche).

The stability of the products used in the study will be known to assure suitability for use throughout the study.
Drug supplies should be stored in a secure area until use.

3.12 Packaging and Labelling

Each centre will receive a number of packages containing tablets of study drugs. Each package will be identified by a label indicating at least the following information:

- Product name
- Trial reference
- Name and address of the Sponsor
- Lot number and expiry date
- Storage directions
- Date dispensed
- Patient number

3.13 Dosage and Dosing Schedule

Dihydroartemisinin (DHA, 20 mg or 40 mg) and Piperaquine (PPQ, 160 or 320 mg) = Artekin (Sigma-Tau)

Medication will be administered approximately at the following times:

Day 0: hour "X"
Day 1: hour "X" + 24 hours
Day 2: hour "X" + 48 hours

The number of tablets will be based on bodyweight (see the relevant table in Appendix II).

Artesunate (AS) and Mefloquine (MQ)

Medications will be administered approximately at the following times:
Artesunate (50 mg): Day 0, D1 (Day 0+24 hours), D2 (Day 0+48 hours)
Mefloquine (250 mg): D1 (Day 0+24 hours), D2 (Day 0+48 hours)

Mefloquine will be administered at 15 mg/kg on Day 1 and 10 mg/kg on Day 2.

3.14 Drug Dispensing

The study medication will be administered at the clinic. Study medications given to young children will be crushed, mixed with water, and administered as a slurry if they are unable to swallow. Study medications administered to older children and adults will be given as tablets or fractions of tablets to be taken orally with a glass of water. The investigator or the study nurse will directly observe consumption of study medications. Patients will be observed for 1 hour to ensure that the medications are not vomited or spat out. Any patient who vomits the medication within 30 minutes of administration will be retreated with a second dose. Vomiting 30 min- 1 hour afterwards, half dose again will be given.

4 CONCOMITANT THERAPIES

4.1 Concomitant Drug Therapies Disallowed

Any antimalarial, or antibiotic with antimalarial activity (erythromycin or other macrolides, co-trimoxazole or other sulfonamides, any tetracycline including doxycycline, and quinolones). The exception to this is chloroquine which may be prescribed for non-falciparum infections during follow up.

4.2 Concomitant Drug Therapies Allowed

During the trial patients can receive certain prescribed drugs e.g. paracetamol, and non malarial antibiotics (penicillins, cephalosporins), chloroquine.
Haematinics where indicated after day 7 can be prescribed.
In case of microscopic evidence of ova/cysts/parasites, or if adult worms are visualized (in stool or vomit), drugs (mebendazole) can be prescribed.

4.3 Special Conditions

Any concurrent treatments that are prescribed by the study physicians will be recorded and administered on site while the patient is in the clinic. Patients, parents or guardians will be discouraged from obtaining drugs from private pharmacies or physicians. Parents/guardians will be told to bring their children to the study clinic if their child is unwell or are worried about their child's health.

4.4 Rescue Treatments

Patients with treatment failure will be withdrawn from the study, treated, and followed up as per local practice. They will not have study investigations performed thereafter.
Patients who require clinical rescue therapy (ETF, LCF, or LPF) will be treated with artesunate 2mg/kg/ day for 7 days plus doxycycline if no contraindication. Any patient who is diagnosed with severe malaria or danger signs during follow-up, will be referred for appropriate treatment with parenteral artesunate at the local facility.

5 PATIENT WITHDRAWAL CRITERIA

During the study, the following conditions are reasons for excluding the patients from further participation in the trial:

1) Use of antimalarial drugs outside of the study protocol.
2) Withdrawal of informed consent.
3) Lost to follow-up: patients who fail to attend a follow-up visit and are unable to be located within 48 hours on Days 1-14 or for more than 2 subsequent visits between Days 15-63.
4) Patient's request to discontinue for any reason.
5) At the investigator's request for safety reasons.

6 SAFETY VARIABLES

Safety and tolerability of the treatments will be evaluated by recording Adverse Events (AEs), laboratory, ECG and vital signs evaluations.

6.1 Adverse Events

At each visit, the Investigator will ask the patient about the occurrence of any adverse events during the preceding period. Any event must be recorded on the appropriate CRF.

6.1.1 Definition of an Adverse Event

An AE is any untoward medical occurrence in a patient or clinical investigation subject administered a pharmaceutical product and which does not necessarily have a causal relationship with this treatment.
An AE can therefore be any unfavourable and unintended sign (that could include a clinically significant abnormal laboratory finding), symptom or disease temporally associated with the use of a

medicinal product, whether or not considered related to the medicinal product.

6.1.2 Severity, Relationship of Event to Study Drug, and outcome

The severity of an adverse event is to be scored according to the following scale :

1	Mild	Awareness of sign or symptom, but easily tolerated
2	Moderate	Discomfort enough to cause interference with usual activity
3	Severe	Incapacitating with inability to work or perform usual activity

The relationship of an adverse event to study drug is to be assessed according to the following definitions :

1 Definitely unrelated

Should be reserved for those events which occur prior to test drug administration (e.g., washout or single-blind placebo) or for those events which cannot be even remotely related to study participation (e.g. injury caused by a third party).

2 Unlikely

There is no reasonable temporal association between the study drug and the suspected event and the event could have been produced by the subject's clinical state or other modes of therapy administered to the subject.

3 Possible

The suspected adverse event may or may not follow a reasonable temporal sequence from study drug administration but seems to be the type of reaction that cannot be dismissed as unlikely. The event could have been produced or mimicked by the subject's clinical state or by other modes of therapy concomitantly administered to the subject.

4 Probable

The suspected adverse event follows a reasonable temporal sequence from study drug administration, abates upon discontinuation of the drug, and cannot be reasonably explained by the known characteristics of the subject's clinical state.

5 Definitely related

Should be reserved for those events which have no uncertainty in their relationship to test drug administration: this means that a rechallenge was positive.

The outcome of each AE must be assessed according to the following classification:

- completely recovered :	The patient has fully recovered with no observable residual effects
- not yet completely recovered :	Improvement in the patient’s condition has occurred, but the patient still has some residual effects
- deterioration :	The patient’s overall condition has worsened
- permanent damage :	The AE has resulted in a permanent impairment
- death :	the patient died due to the AE
- ongoing :	the AE has not resolved and remains the same as at onset
- unknown :	The outcome of the AE is not known because the patient did not return for follow-up (lost to follow-up)

6.1.3 Defintion of a Serious Adverse Event

A serious adverse event (experience) (SAE) or reaction is any untoward medical occurrence that at any dose :

* results in death;
* is life-threatening;
* requires in subject hospitalization (other than for drug administration) or prolongation of existing hospitalization;
* results in persistent or significant disability/incapacity; or
* is a congenital anomaly/birth defect.

And also

* other important medical events (jeopardise the subject or require intervention to prevent one of the other outcomes listed in the definition above).

All serious adverse events, whether or not deemed drug-related, or expected, must be reported immediately or within 24 hours (one working day), using the Serious Adverse Event Notification Form , by telefax to :

Dr Souad Amel Kechairi
MDS Pharma Services
Safety Group

Fax: +33 1 46 90 26 27
(Phone: +33 1 46 90 26 65)

Fax should state "Urgent Serious Adverse Event" on cover page.
MDS Pharma Services will be in charge of SAE notifications to relevant Authorities, according to local regulations.
MDS Pharma Services must forward the report to Sigma-Tau to the attention of Dr Marco Corsi, (fax number +39 06 91393757) within one working day.
A written first follow-up report from the Investigator must be provided to MDS Pharma Services within 5 working days and is to include a full description of the event and sequelae.
All other AEs not fulfilling the criteria of immediate reporting must be recorded on the Case Report Form. This AE information will be collected on a regular basis during the clinical trial by the Clinical Research Associate of MDS Pharma Services.

6.1.4 Reporting of adverse events

For all adverse events identified, an adverse event report form will be completed.

For each possible adverse event identified and graded as moderate, severe, an adverse event report form will be completed. An adverse event report form will not be completed for events classified as mild as these symptoms are common and difficult to distinguish from signs and symptoms due to malaria.

The following information will be recorded for all adverse events:

1) Description of event
2) Date of event onset
3) Date event reported
4) Severity of the event
5) Relationship of the event to study medication
6) Is the event serious ?
7) Initials of the person reporting the event
8) Was the event episodic or intermittent in nature ?

9) Outcome of adverse event
10) Action taken
11) Date event resolved.

A severity grading scale, based on toxicity grading scales developed by the WHO and the National Institutes of Health, Division of Microbiology and Infectious Diseases, will be used to grade severity of all symptoms, physical exam findings, and haemoglobin results (see Appendix I). Any new event, or an event present at baseline that is increasing in severity, will be considered as an adverse event.

6.2 Laboratory Evaluations

Blood samples will be properly labelled with patients' initials, randomisation number, protocol number, and the date the sample is taken. Haematology assessments will be performed locally at sites. Blood chemistry tests will be sent to MDS Pharma Services central laboratory.

All laboratory results will be reported in Standard International Units or in conventional units.

For laboratory analysis, a total volume of blood of about 6 ml will be drawn from each patient/child throughout the study.

a) Haematology and Blood Chemistry Tests
Haemoglobin, haematocrit, red blood cells count, total white blood cell count, differential count, platelet count, total bilirubin, direct bilirubin, alkaline phosphatase, ASAT/ALAT, GGT, urea nitrogen, creatinine, glucose, total proteins, albumin.

b) Urinalysis using dipstick will be performed at site
Glucose, proteins, haemoglobin, ketones, bilirubin.

Abnormal Laboratory Test Results

The Investigator will mark in the CRF the laboratory values out of normal ranges and will indicate those of clinical importance. These will be considered as AEs, and the proper AE reporting procedure should be followed by the Investigator.

6.3 Vital Signs

For a comprehensive safety and tolerability evaluation of the study drug, vital signs (systolic and diastolic blood pressure, heart rate) will be recorded at each visit of the study.
The Investigator should mark in the CRF the heart rate and arterial pressure values out of normal ranges and indicate those of clinical importance. These will be considered as AEs, and the proper AE reporting procedure will be followed by the Investigator.

7 CASE REPORT FORM (CRF)

<u>Presentation of the CRF</u>

The CRF to be used for the study consists of pages headed with the study code and other relevant information. It is composed of an introductive part for the selection and inclusion of patients in the study and special forms for the different evaluation times; at the end of the CRF are the forms for registration of possible adverse events and for any suspension of the study.

<u>How to use the CRF</u>

It is recommended that the CRF be filled out using a ballpoint pen with black ink.
All requested information must be entered on the CRFs. If an item is not available or is not applicable, this fact should be indicated; there should be no blank spaces. A correction should be made by striking through the incorrect entry with a single line and by entering the correct information adjacent to it. The correction must be initialled, dated and explained if necessary by the Investigator or by a qualified individual specifically designated by the Investigator. Each completed Case Report Form must be reviewed, signed and dated by the Investigator.

8 STATISTICS

The statistical analysis will be performed by MDS Pharma Services and detailed in the statistical analysis plan (SAP) that will be prepared within the first month of study initiation.

8.1 Population Analysed

The following populations will be considered for the statistical analysis.

Intention-to-Treat (ITT) Population: Defined as all randomized patients who will take at least one dose of the study treatment.
Evaluable Population (or Per-Protocol (PP) Population): Defined as all randomized patients who will be eligible according to the study protocol, will take at least 80% of the study medication, will not take other anti-malarial drugs and, if presence of malarial symptoms or signs, will have an evaluable PCR.

8.2 Efficacy: primary analysis

The primary analysis will be based on a 97.5% (one-sided) Confidence Interval (CI) computed on the difference between the 63-day PCR corrected cure rates of the test and the reference treatments, respectively.
In order to claim that DHA+PPQ is efficacious, the lower limit of the CI must be > -0.05 and the point estimate for the 63-day PCR corrected cure rate of DHA+PPQ must be > 0.90, in both the ITT and the Evaluable populations.
The method for computing the CI will be specified in the SAP.
All patients who withdrew from the study for one of the reasons listed in section 5 will be evaluated as failures in the ITT population and, if they qualify as Evaluable patients, also in the Evaluable population.
For computing the PCR-corrected cure rate, the following rules will be applied:

- the patients who are diagnosed to be re-infected are counted as successes (they have to be terminated on that day of follow-up);
- the patients for whom the PCR is not interpretable or is missing are counted as failures in the ITT population, while they are excluded from the Evaluable population. The proportion of these patients is less than 10% of patients who experienced a re-infection (i.e. those in whom the PCR evaluation is performed).

Robustness of results for the primary end-point will be investigated by using other analyses, such as the survival techniques.
The Breslow Day test or logistic regression will be used to evaluate homogeneity across centers. In this respect, each center would need to have at least two failures for each treatment in order for large sample chi-square approximations to be applicable. If this type of condition does not apply, exact logistic regression will be used to address homogeneity across centers. In addition, if the sample sizes are sufficiently large within centers so that each treatment has at least five failures within each center, a confidence interval adjusted for centers as strata will be computed (see Koch and others, Statistics in Medicine, 1998).

8.3 Efficacy: secondary analysis

All the secondary statistical analysis will be detailed in the statistical analysis plan (SAP).

8.4 Safety and Tolerability

All randomised patients will be considered for the following analysis.

All Adverse Events (AEs) will be listed by providing adequate details on degree of seriousness, time of treatment at AE onset, severity, relation to study drug, action taken, if any, and final outcome.

Vitals signs and ECG will be analysed descriptively.

ECG results will be classified as normal and abnormal. The evaluation of ECG and QT interval will be done according to the EMEA directive "point to consider: the assessment of the potential for QT interval prolongation by non-cardiovascular medicinal products". EMEA (CPMP), London 17/12/1997

(CPMP/986/96).

9 MONITORING AND QUALITY ASSURANCE

The task of the Monitor is to verify the best conduct of the study through frequent contacts by phone and in person with the Principal Investigator and site staff, in accordance with the Standard Operating Procedures and Good Clinical Practice, with the purposes of facilitating the work and obtaining the objectives of the study. These visits will enable the Monitor to maintain current, personal knowledge of the study through review of the records, comparison with source documents, observation and discussion of the conduct of the study with the Investigator.

The Investigator by signing this protocol declares that he will permit trial-related monitoring, audits, Independent Ethical Committee review, and regulatory inspections, providing direct access to source data/documents.

The Investigator agrees to conduct the present study in full agreement with the principles of the "Declaration of Helsinki" and subsequent relevant amendments (see Appendix III).

10 DATA MANAGEMENT

All data management procedures will be detailed in separate, specifically identified files that collectively will be referenced as the Data Management Plan (DMP).

Appropriate CRFs will be prepared for collection of data requested by the protocol. All response variables will be entered into a database maintained by MDS Pharma services. This database will be compiled and reviewed for accuracy in accordance with MDS Pharma services internal Standard Operating Procedures concerning data management and data quality control. All data will be tabulated to generate data listings for the final report.

These data will be then exported for the creation of Statistical Analysis System (SAS®) datasets that comprise the SAS® database. Edit and consistency checks will also be performed as outlined in the DMP. Once all questions have been be resolved, the database will be locked. The locked SAS® database will be used to generate the subject listings, tabulations, and analyses.

11 INVESTIGATOR RESPONSIBILITY

Except where the Principal Investigator's signature is specifically required, it is understood that the term "Investigator" as used in this protocol and on the CRFs refers to the Principal Investigator or a member of the staff that the Investigator designates to perform a certain duty under this protocol. The Investigator is ultimately responsible for the conduct of all aspects of the study.

For all other relevant Investigator responsibilities see "CPMP/ICH/135/95 Topic E6 - Guideline for Good Clinical Practice", Chapter 4.

12 TRIAL REPORTS

12.1 Statistical Report

The Statistical Analysis will be performed by the Biostatistics and Data Management Department of MDS Pharma Services after having received all the validated CRFs.

12.2 Final Integrated Report

The Final Clinical Study Report will be written by MDS Pharma Services. It is structured as an Integrated Clinical Report in accordance with the ICH guideline E3.

13 ADMINISTRATIVE PROCEDURES

13.1 Regulatory Authorities and Ethical Review Committee

This study will be declared to the National Health Authority.
The clinical protocol will be submitted for approval to the relevant Independent Ethic Committee (IEC) or Institutional Review Board (IRB) before patients can be enrolled.
Copy of the IEC/IRB approval will be transmitted from the Investigator to the Sponsor before starting the study.

13.2 Informed Consent

All interviews will be conducted in the native language of the patients by the study personnel. Consent forms will be provided to the patients / parents or guardians for their review (see Appendix IV). The patients / parents or guardians will be asked to sign consent to participate in a research study. The informed consent will describe the purpose of the study, the procedures to be followed, and the risks and benefits of participation. If a patient, parent or guardian is unable to read or write, a signature from a witness to the informed consent discussion will be obtained. Patients / parents or guardians will be informed that participation in the study is completely voluntary and that they may withdraw from the study at any time.
The age of consent will be considered as > 14 years.

13.3 Confidentiality and Publication of Results

All study documents are provided by the Sponsor in confidence to the investigators and his/her appointed staff. None of this material may be disclosed to any part not directly involved in the study without written permission from Sigma-Tau i.f.r. S.p.A., Pomezia (Rome) – Italy.
Presentation and publication of the study results will be carried out by Investigators jointly with the Sponsor (i.e.: Sigma-Tau), that will be informed well in advance before disclosure of the data, in order to discuss the content of the presentation or manuscript.

13.4 Protocol Amendments

Once the final clinical protocol has been issued and signed by the Investigator and the authorised signatories, it must not be informally altered. Clinical protocol amendments are alterations to a legal document (the clinical protocol) and have the same legal status and must pass through the appropriate steps before being implemented. In general, any change must be approved by the IEC prior to be effective. Administrative changes need only notification to the IEC without approval.
Any subsequent amendments must be made on separate sheet and must pass through the approval process. It must be clear to the Investigator that he can not change the clinical protocol without prior discussion with Sponsor, which should give its approval.

14 Details on study sites

The Shoklo Malaria Research Unit (SMRU) is attached to the Wellcome Trust -Mahidol University, Oxford Tropical Medicine Research Programme in the Faculty of Tropical Medicine, Mahidol University, Bangkok. This unit has worked on the north-western border of Thailand in camps for displaced Karen since 1986 and has conducted antimalarial drug studies in over 10,000 patients and pioneered artemisinin based combination therapy (ACT) and conducted several GCP trials on antimalarials and vaccine. This area has low, seasonal malaria transmission of both multidrug

resistant *P. falciparum* and chloroquine sensitive *P. vivax*. Patients will be recruited from Mae La, a camp for 38 000 displaced persons of the Karen ethnic group 60km north of Mae Sot and from 3 mobile clinics in Wang Pa, Muruchai and Mawker Thai villages along the border south of Mae Sot, serving mainly Karen and Burmese migrant workers.

15 Study Committees

15.1 Steering Committee

The Steering Committee (SC) comprises at least one investigator from each participating region and will assess the progress of the trial. The members of the SC will address policy and operational issues related to the protocol. The SC has responsibility for protecting the scientific conduct and integrity of the trial. Its functions include:

- Review of the protocol before ethic committee approval,
- Formulation of recommendation for any change in the design and operations of the trial during the course of the trial, when needed,
- Exclusion of patients from the per protocol analysis.

The members of the Steering Committee are :
- Professor Umberto D'Alessandro (Prince Leopold Institut of Tropical Medicine,Nationalestraat 155, B-2000 Antwerp-Belgium).
- Dr John Solomon (MDS Pharma Services).
- Professor Nicholas J White (Wellcome Trust Southeast Asian Tropical Medicine Research Units, Faculty of Tropical Medicine Mahidol University, 420/6 Rajvithi Rd, Phayathai 10400, Bangkok, THAILAND).
- Dr. Antonella Bacchieri (Sigma-Tau - Medical Department, Head of Biostatistics and Data Management).

15.2 Data Monitoring Board

The Data Monitoring Board (DMB) will be composed of one statistician and two or three clinicians, all of them independent from the Sponsor and its Designee. At least one of the clinicians will have experience in treating patients with the disease under study and at least one will have expertise in the underlying biology.
The main role of the DMB is that of monitoring all safety data recorded while the study is ongoing and revising in blind a significant percentage (to be defined in the SAP) of the primary efficacy data.

The members of the DMB will be identified prior to enrolling the first patient.

16 PROTOCOL VIOLATION

A protocol violation occurs when a study patient is removed from the study because of an event that does not allow for continued accurate interpretation of response to treatment.
Patients meeting any of the following criteria will be withdrawn from follow-up:

- withdrawal of consent,
- failure to complete the treatment,
- persistent vomiting of study drugs on day 0,
- severe side effects necessitating hospitalisation,
- PCR unclassifiable results,
- Self-medication with antimalarial (or antibiotics with antimalarial activity),
- Severe malaria at Day 0,
- failure to attend enough of the scheduled visits,
- loss to follow-up.

17 REFERENCES

1. **Olliaro P. L. and Taylor W. R. J.**
Review. Antimalarial compounds: from bench to bedside.
*J. Experim. Biol.**206**, 3753-3759, 2003*

2. **World Health Organization**
Antimalarial Drug Combination Therapy. Report of a WHO Technical Consultation. WHO/CDS/RBM/ 2001.35.Geneva, Switzerland: World Health Organization.

3. **White N. J.**
Antimalarial drug resistance: the pace quickens.
*J. Antimicrob. Chemother. **30**, 571-585, 1992*

4. **White, N. J.**
Delaying antimalarial drug resistance with combination chemotherapy.
*Parasitologia **41**, 301-308, 1999*

5. **White, N. J., Nosten, F., Looareesuwan, S., Watkins, W. M., Marsh, K., Snow, R. W., Kokwaro, G., Ouma, J., Hien, T. T., Molyneux, M. E. et al.**
*Averting a malaria disaster. Lancet **353**, 1965-1967, 1999*

6. **White, N. J.**
Assessment of the pharmacodynamic properties of antimalarial drugs in vivo.
*Antimicrob. Agents Chemother. **41**, 1413-1422, 1997*

7. **Kumar, N. and Zheng, H.**
Stage-specific gametocytocidal effect in vitro of the antimalaria drug qinghaosu on Plasmodium *falciparum*.
*Parasitol. Res. 7**6**, 214-218, 1990*

8. **Targett, G., Drakeley, C., Jawara, M., von Seidlein, L., Coleman, R., Deen, J., Pinder, M., Doherty, T., Sutherland, C., Walraven, G. et al.**
Artesunate reduces but does not prevent posttreatment transmission of Plasmodium *falciparum* to *Anopheles gambiae*.
*J. Infect. Dis. **183**, 1254-1259, 2001*

9. **Price, R. N., Nosten, F., Luxemburger, C., ter Kuile, F. O., Paiphun, L., Chongsuphajaisiddhi, T. and White, N. J.**
Effects of artemisinin derivatives on malaria transmissibility.
*Lancet **347**, 1654-1658, 1996*

10. **Nosten, F., van Vugt, M., Price, R., Luxemburger, C., Thway, K. L., Brockman, A., McGready, R., ter Kuile, F., Looareesuwan, S. and White, N. J.**
Effects of artesunate-mefloquine combination on incidence of Plasmodium falciparum malaria and Mefloquine resistance in western Thailand: a prospective study.
*Lancet **356**, 297-302, 2000*

11. **White, N. J. and Olliaro, P.**
Artemisinin and derivatives in the treatment of uncomplicated malaria.
*Med. Trop. **58**(Suppl), 54-56, 1998*

12. **McGready, R., Cho, T., Keo, N. K., Thwai, K. L., Villegas, L., Looareesuwan, S., White, N. J. and Nosten, F.**

Artemisinin antimalarials in pregnancy: a prospective treatment study of 539 episodes of multidrug-resistant Plasmodium *falciparum*.
Clin. Infect. Dis. ***33****, 2009-2016, 2001*

13. **Trouiller, P., Olliaro, P., Torreele, E., Orbinski, J., Laing, R. and Ford, N.**
Drug development for neglected diseases: a deficient market and a public-health policy failure.
Lancet ***359****, 2188-2194, 2002*

14. **Sulo, J., Chimpeni, P., Hatcher, J., Kublin, J. G., Plowe, C. V., Molyneux, M. E., Marsh, K., Taylor, T. E., Watkins, W. M. and Winstanley, P. A.**
Chlorproguanil-dapsone versus sulfadoxine-pyrimethamine for sequential episodes of uncomplicated *falciparum* malaria in Kenya and Malawi: a randomised clinical trial.
Lancet ***360****, 1136-1143, 2002*

15. **Mutabingwa, T., Nzila, A., Mberu, E., Nduati, E., Winstanley, P., Hills, E. and Watkins, W.**
Chlorproguanil-dapsone for treatment of drug resistant falciparum malaria in Tanzania.
Lancet ***358****, 1218-1223, 2001*

16. **Omari, A. A., Preston, C. and Garner, P.**
Artemether-lumefantrine for treating uncomplicated falciparum malaria.
Cochrane Database Syst. Rev. ***2****, CD003125, 2003*

17. **Vugt, M. V., Wilairatana, P., Gemperli, B., Gathmann, I., Phaipun, L., Brockman, A., Luxemburger, C., White, N. J., Nosten, F. and Looareesuwan, S.**
Efficacy of six doses of artemether-lumefantrine (benflumetol) in multidrug-resistant Plasmodium *falciparum* malaria.
Am. J. Trop. Med. Hyg. ***60****, 936-942, 1999*

18. **van Vugt, M., Looareesuwan, S., Wilairatana, P., McGready, R., Villegas, L., Gathmann, I., Mull, R., Brockman, A., White, N. J. and Nosten, F.**
Artemether-lumefantrine for the treatment of multidrug-resistant falciparum malaria.
Trans. R. Soc. Trop. Med. Hyg. ***94****, 545-548, 2000*

19. **Ringwald, P., Bickii, J. and Basco, L.**
Randomised trial of pyronaridine versus chloroquine for acute uncomplicated falciparum malaria in Africa.
Lancet ***347****, 24-28, 1996*

20. **Looareesuwan, S., Kyle, D. E., Viravan, C., Vanijanonta, S., Wilairatana, P. and Wernsdorfer, W. H.**
Clinical study of pyronaridine for the treatment of acute uncomplicated falciparum malaria in Thailand. *Trans. R.*
Soc. Trop. Med. Hyg. ***54****, 205-209, 1996*

21. **S. Looareesuwan, V. Navaratnam and P. L. Olliaro**
Unpublished, personal communication.

22. **Lell, B., Ruangweerayut, R., Wiesner, J., Missinou, M. A., Schindler, A., Baranek, T., Hintz, M., Hutchinson, D., Jomaa, H. and Kremsner, P.G.**
Fosmidomycin, a novel chemotherapeutic agent for malaria.
Antimicrob. Agents Chemother. ***47****, 735-738, 2003*

23. **Missinou, M. A., Borrmann, S., Schindler, A., Issifou, S., Adegnika, A. A., Matsiegui, P. B., Binder, R., Lell, B., Wiesner, J., Baranek, T. et al.**
Fosmidomycin for malaria.
Lancet ***360****, 1941-1942, 2002*

24. **Denis, M. B., Davis, T. M., Hewitt, S., Incardona, S., Nimol, K., Fandeur, T., Poravuth, Y., Lim, C. and Socheat, D.**
Efficacy and safety of dihydroartemisinin-piperaquine (Artekin) in Cambodian children and adults with uncomplicated falciparum malaria.
Clin. Infect. Dis. ***35****, 1469-1476, 2002*

25. **Wilairatana, P., Krudsood, S., Chalermut, K., Pengroska, C., Srivilairit, S., Silachamroon, U., Treeprasertsuk, S. and Looareesuwan, S.**
An open randomized clinical trial of Artecom vs artesunate-mefloquine in the treatment of acute uncomplicated falciparum malaria in Thailand.
Southeast Asian J Trop. Med. Public Health ***33(3),*** *519-524, 2002*

26. **Hein, T.T., Dolecek, C., Mai, P.P., Dung, N.T., Truong, N.T., Thai, L.H., An, D.T.A., Thanh, T.T., Stepniewska, K., White, N., and Farrar, J.**
Dihydroartemisinin-piperaquine against multidrug-resistant Plasmodium *falciparum* malaria in Vietnam: randomized clinical trial.
Lancet ***363****, 18-22, 2004*

27. **Karunajeewa, H., Lim, C., Hung, T.Y., Ilett, K.F., Denis, M.B., Socheat, D. and Davis, T.M.E.**
Safety evaluation of fixed combination piperaquine plus dihydroartemisinin (Artekin) in Cambodian children and adults with malaria.
Br. J. Clin. Pharmacol. 57, 93-99, 2003

28. **Hung, T.Y., Davis, T.M.E., Ilett, K.F., Karunajeewa, H., Hewitt, S., Denis, M.B., Lim, C. and Socheat, D.**
Population pharmacokinetics of piperaquine in adults and children with uncomplicated falciparum or vivax malaria.
Br. J. Clin. Pharmacol. 57:3, 253-262, 2003

29. **Ashley, E.A., Krudsood, S., Phaiphun, L., Srivilairit, S., McGready, R., Leowattana, W., Hutagalung, R., Wilairatana, P., Brockman, A., Looareesuwan, S., Nosten, F., White, N.J.**
Dose optimization randomized controlled studies of dihydroartemisinin-piperaquine for the treatment of uncomplicated multi-drug resistant falciparum malaria in Thailand.
Personal communication, 2004

30. **Myint,H., Ashley, E. and White, N.**
Reported adverse effects of dihydroartemisinin-piperaquine (Artekin)
Personal communication, 2004

31. **Danis, M., Bricaire, F.**
The new drug combinations: their place in the treatment of uncomplicated Plasmodium falciparum malaria
Fundam. Clin. Pharmacol. ***17****, 155-160, 2003*

18 APPENDICES

APPENDIX I

Guidelines for Grading Patient Symptoms, signs and laboratory findings.

Table A. Guidelines for Grading Patient Symptoms.

	**Grade 1**<br>**MILD**	**Grade 2**<br>**MODERATE**	**Grade 3**<br>**SEVERE**	**Grade 4**<br>**LIFE THREATENING**
**Subjective fever in the past 24 h**	N/A	Present (Yes)	N/A	N/A
Weakness	Mild decrease in activity; For children – weak, but still playing	Moderate decrease in activity; For children – weak, and playing limited	Not participating in usual activities; For children – not playing	Prostration
**Muscle and/or joint aches***	Mild and/or localized complaints	Diffuse complaints	Objective weakness; function limited	N/A
**Headache***	Mild, no treatment required	Transient, moderate; treatment required	Severe, constant; requires narcotic therapy	Intractable; requires repeated narcotic therapy
**Anorexia**	Decreased appetite, but still taking solid food	Decreased appetite, avoiding solid food but taking liquids	Appetite very decreased; Refusing to breast feed, no solids or liquids taken (< 2 years ≤ 12 hr; > 2 years ≤ 24 hr)	Appetite very decreased; Refusing to breast feed, no solids or liquids taken (< 2 years > 12 hr; > 2 years > 24 hr)
**Nausea***	Mild, transient feeling of impending vomiting; maintains reasonable intake	Moderate and/or constant feeling of impending vomiting; intake decreased	Severe, constant feeling of impending emesis; intake decreased significantly	N/A
**Vomiting**	1 episode per day	2-3 episodes per day	Orthostatic hypotension or IV fluids required	Hypotensive shock or physiologic consequences requiring IV fluid therapy
**Abdominal pain***	Mild (1-3 on a scale of 1 to 10)	Moderate (4-6 on a scale of 1 to 10)	Moderate to severe (≥ 7 on a scale of 1 to 10)	Severe – hospitalization for treatment
**Diarrhea**	Transient 3-4 loose stools/day	5-7 loose stools/day	Orthostatic hypotension or > 7 loose stools/day or IV fluids required	Hypotensive shock or physiologic consequences requiring IV fluid therapy
**Cough**	Transient / intermittent	Persistent / constant	Uncontrolled	Cyanosis, stridor, severe shortness of breath
**Pruritis**	Transient pruritis	Pruritis that disturbs sleep	Severe, constant pruritis, sleep disturbed	N/A
**Tinnitus***	Mild, transient ringing or roaring sound	Moderate, persistent ringing or roaring sound	Severe ringing or roaring sound with associated hearing loss	N/A
**Behavioural changes**	Mild difficulty concentrating; mild confusion or agitation; activities of daily living unaffected; no treatment	Moderate confusion or agitation; some limitation of activities of daily living; minimal treatment	Severe confusion or agitation; Needs assistance for activities of daily living; therapy required	Toxic psychosis; □nrolment□ation required
**“Flu”**<br>**(viral URI)**	Mild nasal congestion, mild rhinorrhea	Moderate nasal congestion, moderate rhinorrhea	N/A	N/A
**Allergic reaction**	N/A	N/A	Urticaria	Severe urticaria anaphylaxis, angioedema
**Convulsion**	N/A	N/A	Localized or generalized seizure	Status epilepticus
*** Assess only in children ≥ 3 years of age. Answer N/A for younger children and those unable to answer.**				

Reference – Based on WHO Toxicity Grading Scale for Determining the Severity of Adverse Events

Table B. Guidelines for Physical Examination

**Dehydration**	Assess skin touch and turgor, mucous membranes, eyes, crying, fontanelle, pulse, urine output
**Jaundice**	Assess for yellowing of the sclera. Also evaluate the palpebral conjunctiva, lips, and skin.
**Chest**	Observe the rate, rhythm, depth, and effort of breathing. Check the patient's colour for cyanosis.<br><br>The maximum acceptable respiratory rate by age: < 2 months = 60, 2-12 months = 50, 1-5 years = 40, above 5 years = 30.<br><br>Inspect the neck for the position of the trachea, for supraclavicular retractions, and for contraction of the sternomastoid or other accessory muscles during inspiration.<br><br>Auscultate the anterior and posterior chest for normal breath sounds and any adventitious sounds (crackles or rales, wheezes, and rhonchi). *Crackles are intermittent, non-musical, fine or coarse sounds that may be due to abnormalities of the lungs (pneumonia, fibrosis, early congestive heart failure) or airways (bronchitis or bronchiectasis). Wheezes are high-pitched and result from narrowed airways. Rhonchi are relatively low-pitched and suggest secretions in large airways.*<br><br>If abnormalities are identified, evaluate for transmitted voice sounds. In addition, palpate the chest to assess for tactile fremitus, and percuss the chest to assess for areas of dullness. *Normal, air-filled lungs emit predominantly vesicular breath sounds, transmit voice sounds poorly with "ee" = "ee", and have no tactile fremitus. Airless lung, as in lobar pneumonia, emits bronchial breath sounds, transmits spoken words clearly with "ee" = "aay" (egophany), and has an increase in tactile fremitus.*
**Abdomen**	Inspect and ausculate the abdomen. Listen for bowel sounds in the abdomen before palpating it. Palpate the abdomen in all 4 quadrants lightly and then deeply. Assess the size of the liver and spleen. To assess for peritoneal inflammation, look for localised and rebound tenderness, and voluntary or involuntary rigidity.
**Skin**	Inspect the skin for colour, turgor, moisture, and lesions. If lesions are present, note their location and distribution (diffuse or localised), arrangement (linear, clustered, annular, dermatomal), type (macules, papules, vesicles) and colour.
**Tablet test**	For children ≥ 9 months of age, ask the patient to pick a tablet (or equivalent object) up off a flat surface using the thumb and index finger of their dominant hand. *This tests for co-ordination of the upper extremity assessing the function of the motor system, cerebellar system, vestibular system (for coordinating eye and body movements) and the sensory system, for position sense. When testing small children, be aware that they will likely attempt to put the object into their mouth.*

Table C. Grading Physical Examination Findings

	Grade 1 MILD	Grade 2 MODERATE	Grade 3 SEVERE	Grade 4 LIFE-THREATENING
**Temperature* (axillary)**	37.5-37.9°C	38.0-39.5°C	> 39.5°C	Sustained fever, equal or greater than 40.0°C for longer than 5 days
**Dehydration**	Less than 2 of the following:<br>Restless, irritable<br>Sunken eyes<br>Drinks eagerly, thirsty<br>Skin pinch goes back slowly	2 of the following:<br>Restless, irritable<br>Sunken eyes<br>Drinks eagerly, thirsty<br>Skin pinch goes back slowly	Two of the following:<br>Lethargic or unconscious<br>Sunken eyes<br>Not able to drink or drinking poorly<br>Skin pinch goes back very poorly	Two of the following + shock:<br>Lethargic or unconscious<br>Sunken eyes<br>Not able to drink or drinking poorly<br>Skin pinch goes back very poorly
**Jaundice**	Slight yellowing of sclera and conjunctiva	Moderate yellowing of sclera and conjunctiva, yellowing of mucous membranes	Severe yellowing of sclera and conjunctiva, yellowing of skin	N/A
**Chest**	Mildly increased RR (for age, temperature), transient or localised adventitious sounds	Moderately increased RR, diffuse or persistent adventitious sounds	Rapid RR (< 2 months > 60, 2-12 months > 50, 1-5 years > 40, adults > 30)* nasal flaring, retractions	Cyanosis
**Abdomen**	Normal bowel sounds, mild localised tenderness, and/or liver palpable 2-4 cm below the right costal margin (RCM), and/or spleen palpable, and/or umbilical hernia present	Normal or mildly abnormal bowel sounds, moderate or diffuse tenderness; and/or mild to moderately enlarged liver (4-6 cm below the RCM) and/or spleen palpable up to half-way between umbilicus and symphysis pubis	Severely abnormal bowel sounds, severe tenderness to palpation. Evidence of peritoneal irritation and/or significant enlargement of liver (> 6 cm below the RCM) and/or spleen palpable beyond half-way between umbilicus and symphysis pubis	Absent bowel sounds. Involuntary rigidity
**Skin†**	Localised rash, erythema, or pruritis	Diffuse, maculopapular rash, dry desquamation	Vesiculation, moist desquamation, or ulceration	Exfoliative dermatitis, mucous membrane involvement or erythema multiforme or suspected Stevens-Johnson or necrosis requiring surgery

	Grade 1<br>MILD	Grade 2<br>MODERATE	Grade 3<br>SEVERE	Grade 4<br>LIFE-THREATENING
**Hearing**	*< 4 years: N/A*<br>≥ 4 years: Decreased hearing in one ear	*< 4 years: N/A*<br>≥ 4 years: Decreased hearing in both ears or severe impairment in one ear	*< 4 years: Any evidence of hearing impairment*<br>≥ 4 years: Severe impairment in both ears	N/A
**Tablet test**	Difficulty grasping tablet but able to pick up	Unable to pick up tablet without dropping	Unable to grasp tablet	N/A
**Clinical symptoms / sign *(not otherwise specified)***	No treatment required; monitor condition	Treatment required	Requires treatment and possible hospitalisation	Requires active medical intervention, hospitalisation, or hospice care

Reference – The Harriet Lane Handbook, 15th edition, 2000
† Reference – WHO Toxicity Grading Scale for Determining the Severity of Adverse Events

TABLE D. Guidelines for Grading of Laboratory Abnormalities

	Grade 1<br>MILD	Grade 2<br>MODERATE	Grade 3<br>SEVERE	Grade 4<br>LIFE-THREATENING
**Haemoglobin** *(g/dL)*	9.0 – 9.9	7.0 – 8.9	5.0 – 6.9	< 5.0

Reference – The Harriet Lane Handbook, 15th edition, 2000†
Reference – WHO Toxicity Grading Scale for Determining the Severity of Adverse Events

APPENDIX II

Table A. Dihydroartemisinin will be given daily. The number of tablets per day is reported in brackets. One tablet of Artekin (pediatric tablets) contains 20 mg of DHA and 160 mg of PPQ. One tablet of Artekin (adult tablets) contains 40 mg of DHA and 320 mg of PPQ.

Weight in kg	mg of DHA to be given daily	Dose of DHA as mg/kg/d
4 - 6	10 (1/2 tablet with 20 mg)	1.67 - 2.5
7 - 12	20 (1 tablet with 20 mg)	1.67 - 2.86
13 - 23	40 (1 tablet with 40 mg)	1.74 - 3.08
24 - 35	80 (2 tablets with 40 mg)	2.29 - 3.33
36 - 75	120 (3 tablets with 40 mg)	1.6 - 3.64

Table B: Piperaquine will be given daily. The number of tablets per day is reported in brackets. One tablet of Artekin (pediatric tablets) contains 20 mg of DHA and 160 mg of PPQ. One tablet of Artekin (adult tablets) contains 40 mg of DHA and 320 mg of PPQ.

Weight in kg	mg of PPQ to be given daily	Dose of PPQ as mg/kg/d
4 - 6	80 (1/2 tablet with 160 mg)	13.36 - 20
7 - 12	160 (1 tablet with 160 mg)	13.36 – 22.88
13 - 23	320 (1 tablet with 320 mg)	13.92 – 24.64
24 - 35	640 (2 tablets with 320 mg)	18.32 – 26.4
36 - 75	960 (3 tablets with 320 mg)	12.8 – 29.12

SMRU mefloquine + artesunate dosing :

Artesunate 50 mg tablets (a Suspension (10 mg/ml) is made by dissolving 1 tablet in 5 ml water)

Weight (kg)	4 mg/kg (OD)	
	tab	ml
5		2.0
6	1/2	2.4
7	1/2	2.8
8	3/4	3.2
9	3/4	3.6
10	3/4	4.0
11	1	4.4
12	1	4.8
13 - 14	1	
15 - 16	1 1/4	
17 - 20	1 1/2	
21-23	1 3/4	
24 - 26	2	
27 - 29	2 1/4	
30 - 32	2 1/2	
33 - 35	2 3/4	
36 - 39	3	
40-42	3 1/4	
43 - 45	3 1/2	
46-48	3 3/4	
49 - 51	4	
52 - 54	4 1/4	
55 - 57	4 1/2	
58 - 60	4 3/4	
61 - 64	5	
65-67	5 1/4	
68 - 70	5 1/2	

Mefloquine 250mg (base) tablets given as 15mg/kg and 10mg/kg on the second and third days of treatment respectively (A suspension (50mg/ml) is made by dissolving 1 tablet in 5 mls water)

kg	d1, (15 mg/kg)		d2, (10 mg/kg)	
	tabs	(or mls)	tabs	(or mls)
5	1/4	1.5	1/4	1.0
6	1/4	1.8	1/4	1.2
7	1/2	2.1	1/4	1.4
8	1/2	2.4	1/4	1.6
9	1/2	2.7	1/2	1.8
10	1/2	3.0	1/2	2.0
11	1/2	3.3	1/2	2.2
12	3/4	3.6	1/2	2.4
13	3/4	3.9	1/2	2.6
14	3/4	4.2	1/2	2.8
15	1	4.5	1/2	3.0
16	1		1/2	
17-18	1 1/4		1/2	
19-21	1 1/4		3/4	
22-23	1 1/2		3/4	
2426	1 1/2		1	
27-28	1 3/4		1	
29-31	1 3/4		1 1/4	
32-33	2		1 1/4	
34-36	2		1 1/2	
37-38	2 1/4		1 1/2	
39-41	2 1/2		1 1/2	
42-43	2 1/2		1 3/4	
44-46	2 3/4		1 3/4	
47-48	2 3/4		2	
49-51	3		2	
52-53	3 1/4		2	
54-56	3 1/4		2 1/4	
57-58	3 1/2		2 1/4	
59-61	3 1/2		2 1/2	
62-63	3 3/4		2 1/2	
64-66	4		2 1/2	
67-68	4		2 3/4	
69-71	4		3	

APPENDIX III

WORLD MEDICAL ASSOCIATION DECLARATION OF HELSINKI

Ethical Principles for Medical Research Involving Human Subjects

Recommendations guiding medical physicians in biomedical research involving human subjects

Adopted by the 18th WMA General Assembly
Helsinki, Finland, June 1964
and amended by the
29th WMA General Assembly, Tokyo, Japan, October 1975
35th WMA General Assembly, Venice, Italy, October 1983
41st WMA General Assembly, Hong Kong, September 1989
48th WMA General Assembly, Somerset West, Republic of South Africa, October 1996
and the
52nd WMA General Assembly, Edinburgh, Scotland, October 2000
Note of Clarification on Paragraph 29 added by the WMA General Assembly, Washington 2002.

A. INTRODUCTION

1. The World Medical Association has developed the Declaration of Helsinki as a statement of ethical principles to provide guidance to physicians and other participants in medical research involving human subjects. Medical research involving human subjects includes research on identifiable human material or identifiable data.

2. It is the duty of the physician to promote and safeguard the health of the people. The physician's knowledge and conscience are dedicated to the fulfillment of this duty.

3. The Declaration of Geneva of the World Medical Association binds the physician with the words, "The health of my subject will be my first consideration," and the International Code of Medical Ethics declares that, "A physician shall act only in the subject's interest when providing medical care which might have the effect of weakening the physical and mental condition of the subject."

4. Medical progress is based on research which ultimately must rest in part on experimentation involving human subjects.

5. In medical research on human subjects, considerations related to the well-being of the human subject should take precedence over the interests of science and society.

6. The primary purpose of medical research involving human subjects is to improve prophylactic, diagnostic and therapeutic procedures and the understanding of the etiology and pathogenesis of disease. Even the best proven prophylactic, diagnostic, and therapeutic methods must continuously be challenged through research for their effectiveness, efficiency, accessibility and quality.

7. In current medical practice and in medical research, most prophylactic, diagnostic and therapeutic procedures involve risks and burdens.

8. Medical research is subject to ethical standards that promote respect for all human beings and protect their health and rights. Some research populations are vulnerable and need special protection. The particular needs of the economically and medically disadvantaged must be recognized. Special attention is also required for those who cannot give or refuse consent for themselves, for those who may be subject to giving consent under duress, for those who will not benefit personally from the research and for those for whom the research is combined with care.

9. Research Investigators should be aware of the ethical, legal and regulatory requirements for research on human subjects in their own countries as well as applicable international requirements. No national ethical, legal or regulatory requirement should be allowed to reduce or eliminate any of the protections for human subjects set forth in this Declaration.

B. BASIC PRINCIPLES FOR ALL MEDICAL RESEARCH

10. It is the duty of the physician in medical research to protect the life, health, privacy, and dignity of the human subject.

11. Medical research involving human subjects must conform to generally accepted scientific principles, be based on a thorough knowledge of the scientific literature, other relevant sources of information, and on adequate laboratory and, where appropriate, animal experimentation.

12. Appropriate caution must be exercised in the conduct of research which may affect the environment, and the welfare of animals used for research must be respected.

13. The design and performance of each experimental procedure involving human subjects should be clearly formulated in an experimental protocol. This protocol should be submitted for consideration, comment, guidance, and where appropriate, approval to a specially appointed ethical review committee, which must be independent of the Investigator, the Sponsor or any other kind of undue influence. This independent committee should be in conformity with the laws and regulations of the country in which the research experiment is performed. The committee has the right to monitor ongoing trials. The researcher has the obligation to provide monitoring information to the committee, especially any serious adverse events. The researcher should also submit to the committee, for review, information regarding funding, Sponsors, institutional affiliations, other potential conflicts of interest and incentives for subjects.

14. The research protocol should always contain a statement of the ethical considerations involved and should indicate that there is compliance with the principles enunciated in this Declaration.

15. Medical research involving human subjects should be conducted only by scientifically qualified persons and under the supervision of a clinically competent medical person. The responsibility for the human subject must always rest with a medically qualified person and never rest on the subject of the research, even though the subject has given consent.

16. Every medical research project involving human subjects should be preceded by careful assessment of predictable risks and burdens in comparison with foreseeable benefits to the subject or to others. This does not preclude the participation of healthy volunteers in medical research. The design of all studies should be publicly available.

17. Physicians should abstain from engaging in research projects involving human subjects unless they are confident that the risks involved have been adequately assessed and can be satisfactorily managed. Physicians should cease any investigation if the risks are found to outweigh the potential benefits or if

there is conclusive proof of positive and beneficial results.

18. Medical research involving human subjects should only be conducted if the importance of the objective outweighs the inherent risks and burdens to the subject. This is especially important when the human subjects are healthy volunteers.

19. Medical research is only justified if there is a reasonable likelihood that the populations in which the research is carried out stand to benefit from the results of the research.

20. The subjects must be volunteers and informed participants in the research project.

21. The right of research subjects to safeguard their integrity must always be respected. Every precaution should be taken to respect the privacy of the subject, the confidentiality of the subject's information and to minimize the impact of the study on the subject's physical and mental integrity and on the personality of the subject.

22. In any research on human beings, each potential subject must be adequately informed of the aims, methods, sources of funding, any possible conflicts of interest, institutional affiliations of the researcher, the anticipated benefits and potential risks of the study and the discomfort it may entail. The subject should be informed of the right to abstain from participation in the study or to withdraw consent to participate at any time without reprisal. After ensuring that the subject has understood the information, the physician should then obtain the subject's freely-given informed consent, preferably in writing. If the consent cannot be obtained in writing, the non-written consent must be formally documented and witnessed.

23. When obtaining informed consent for the research project the physician should be particularly cautious if the subject is in a dependent relationship with the physician or may consent under duress. In that case the informed consent should be obtained by a well-informed physician who is not engaged in the investigation and who is completely independent of this relationship.

24. For a research subject who is legally incompetent, physically or mentally incapable of giving consent or is a legally incompetent minor, the Investigator must obtain informed consent from the legally authorized representative in accordance with applicable law. These groups should not be included in research unless the research is necessary to promote the health of the population represented and this research cannot instead be performed on legally competent persons.

25. When a subject deemed legally incompetent, such as a minor child, is able to give assent to decisions about participation in research, the Investigator must obtain that assent in addition to the consent of the legally authorized representative.

26. Research on individuals from whom it is not possible to obtain consent, including proxy or advance consent, should be done only if the physical/mental condition that prevents obtaining informed consent is a necessary characteristic of the research population. The specific reasons for involving research subjects with a condition that renders them unable to give informed consent should be stated in the experimental protocol for consideration and approval of the review committee. The protocol should state that consent to remain in the research should be obtained as soon as possible from the individual or a legally authorized surrogate.

27. Both authors and publishers have ethical obligations. In publication of the results of research, the Investigators are obliged to preserve the accuracy of the results. Negative as well as positive results should be published or otherwise publicly available. Sources of funding, institutional affiliations and any possible conflicts of interest should be declared in the publication. Reports of experimentation

not in accordance with the principles laid down in this Declaration should not be accepted for publication.

C. ADDITIONAL PRINCIPLES FOR MEDICAL RESEARCH COMBINED WITH MEDICAL CARE

28. The physician may combine medical research with medical care, only to the extent that the research is justified by its potential prophylactic, diagnostic or therapeutic value. When medical research is combined with medical care, additional standards apply to protect the subjects who are research subjects.

29. The benefits, risks, burdens and effectiveness of a new method should be tested against those of the best current prophylactic, diagnostic, and therapeutic methods. This does not exclude the use of placebo, or no treatment, in studies where no proven prophylactic, diagnostic or therapeutic method exists.

30. At the conclusion of the study, every subject entered into the study should be assured of access to the best proven prophylactic, diagnostic and therapeutic methods identified by the study.

31. The physician should fully inform the subject which aspects of the care are related to the research. The refusal of a subject to participate in a study must never interfere with the subject-physician relationship.

32. In the treatment of a subject, where proven prophylactic, diagnostic and therapeutic methods do not exist or have been ineffective, the physician, with informed consent from the subject, must be free to use unproven or new prophylactic, diagnostic and therapeutic measures, if in the physician's judgment it offers hope of saving life, re-establishing health or alleviating suffering. Where possible, these measures should be made the object of research, designed to evaluate their safety and efficacy. In all cases, new information should be recorded and, where appropriate, published. The other relevant guidelines of this Declaration should be followed.

*** FOOTNOTE: Note of Clarification on Paragraph 29 of the WMA Declaration of Helsinki**

The WMA hereby reaffirms its position that extreme care must be taken in making use of a placebo-controlled trial and that in general this methodology should only be used in the absence of existing proven therapy. However, a placebo-controlled trial may be ethically acceptable, even if proven therapy is available, under the following circumstances:

- Where for compelling and scientifically sound methodological reasons its use is necessary to determine the efficacy or safety of a prophylactic, diagnostic or therapeutic method; or
- Where a prophylactic, diagnostic or therapeutic method is being investigated for a minor condition and the patients who receive placebo will not be subject to any additional risk of serious or irreversible harm.

All other provisions of the Declaration of Helsinki must be adhered to, especially the need for appropriate ethical and scientific review.

APPENDIX IV

RESEARCH PARTICIPANT INFORMED CONSENT FORM

STUDY TITLE

A Phase III, randomized, non-inferiority trial, to assess the efficacy and safety of Dihydroartemisinin+Piperaquine (DHA+PPQ, Artekin) in comparison with Artesunate+Mefloquine (AS+MQ) in patients affected by acute, uncomplicated Plasmodium *falciparum* malaria.

- Multicentre Study In Asia -

Study Protocol No: ST3073-ST3074 DM040010

Please read the background information and informed consent form carefully. The background information explains your rights and our responsibilities to you.if you have any questions concerning the study please do not hesitate to ask any of the doctors. Before you decide, it is important for you to understand why the research is being done and what it will involve. You will be given a copy of this document to take home with you.

<u>YOU MUST KEEP THIS BACKGROUND INFORMATION WITH YOU</u>

<u>THROUGHOUT THE STUDY PERIOD.</u>

PURPOSE OF THE STUDY

This research study is being done to learn more about the treatment of malaria. We would like to know what the best and safest treatment is for malaria in your country. To do this, we are carrying out a research study to compare different combinations of malaria drugs. About 1050 participants will be involved in this study.

HOW THE STUDY IS DONE

You, or the child under your care, will be treated for malaria with dihydroartemisinin + piperaquine (DHA+PPQ) or artesunate + mefloquine (AS+MQ). After the treatment, you (or your child) will be followed up for 63 days to see if the malaria infection is cured. If you (or your child) do not get better after treatment, you will be able to get the treatment according to the usual standard of care in your country. As two antimalaria drugs are being tested, the option given to you or the child will be decided by chance, similar to pulling a number out of a hat. The chance of being placed into each of the treatment groups is one in two. You are being asked to allow yourself, or your child (or the child under your care in the case of a legal guardian), to participate in this study for up to 63 days or until such time as you or the study doctors decide that you (or your child) should no longer participate in the study. The study may be discontinued by the sponsor at any time, and for any reason, for example:

1) If you (or your child) receive malaria medicines not prescribed by the study doctors
2) If you chose to withdraw your consent to participate in the study

3) If we are unable to locate you (or your child) within 48 hours on Days 1-14 or for more than 2 subsequent visits between Days 15-63.

PROCEDURES

1) The study doctors will examine you (or your child) today.
2) A blood sample will be collected. A small amount of blood will be taken by fingerprick to examine for malaria parasites, to measure the haematocrit, to store blood samples on filter paper for future laboratory tests that will not impact on the health care of you (or your child).
3) If you (or your child) are eligible for the study, treatment with dihydroartemisinin + piperaquine (DHA+PPQ) or artesunate + mefloquine (AS+MQ) will be given.
4) You will be asked to return to the clinic at least 12 more times over the next 2 months so that the success of the treatment can be judged. At each of the follow-up visits, you (or your child) will be examined by the study doctors and, a small amount of blood will taken by fingerprick to examine for malaria parasites.
5) If you (or your child) miss an appointment, the home health visitor will visit you at your home to find out why you missed the appointment and bring you (or your child) to the clinic for assessment.
6) If, at any time, the treatment given to you (or your child) does not seem to be working well, it will be changed to treatment according to the usual standard of care.
7) There will be someone at the study clinic every day. You (or your child) can come to the clinic for evaluation anytime that you (or your child) are ill during the next 63 days.
8) For the haematology and biochemistry, there will be 2-3 blood samples: one before the first dose at D0 (Visit 1), the second one at D28 (Visit 8), i.e. one month after D0, and the last one at D63 (Visit 13) in case of abnormality at D28. Each sample will be of 2 mL and will be collected from an arm vein by an experienced nurse. Blood sampling may cause pain and swelling.

RISKS AND DISCOMFORTS

1) Side effects following treatment with the study medications could occur. Generally, side effects (nausea, headache, dizziness…) are mild and short lived.
   You (or your child) will be monitored closely after receiving treatment for malaria with the study medications for any possible side effects of the drugs and will receive appropriate medical care for any problem that happens during the course of the study.
2) The treatment you (or your child) receive may prove to be less effective or to have more side effects than the other study treatments or than other available treatments. This will not be known until after the study is completed.
3) Severe malaria: You (or your child) may develop malaria that is severe even after receiving treatment with study medications. If you (or your child) shows any evidence of severe malaria treatment with the usual standard of care will be given.
4) Blood draws: The amount of blood removed will be too small to affect your (or your child's) health.
5) Unknown Risks: The research treatments may have side effects that no one knows about yet. The researchers will let you know if they learn anything that might make you change your mind about your (or your child's) participation in the study.
6) Confidentiality: Information about you (or your child) will be handled as confidentially as possible. Medical information related to malaria will be collected on your child, but only the people working on the study will see it. Anyone assigned to review this study

will be granted direct access to your child's medical records, if necessary, for verification of the study procedures and data. Records will be kept as confidential as possible.

BENEFITS

1) The potential benefit to you (or your child) is that the treatment received may prove to be more effective than the other study treatments or than other available treatments, although this cannot be guaranteed.
2) You (or your child) will receive clinical care from the medical officers and nurses of the project staff in the study clinic. This will include care for unscheduled sick visits.
3) The knowledge gained from this study will help to determine the best treatment for uncomplicated malaria.

COST/PAYMENT

After enrolment in the study, you will not be charged for clinic visits or treatment. You (or your child) will not be paid for participation in the study. You will be reimbursed for transport costs to and from the clinic for any visit that you (or your child) require.

ALTERNATIVES TO PARTICIPATION

Your (or your child's) participation in this study is completely voluntary. If you decide that you do not want to participate in the study or decide to withdraw yourself (or your child) from the study at any time and for any reason, this will not affect your (or your child's) care at the outpatient department, where standard care for all medical problems is available.

CONSEQUENCES OF WITHDRAWAL

Should you or your study doctors decide to withdraw you (or your child) from the study, you (or your child) will still be eligible for care.

USE OF THE RESULTS

The findings from this study may be published in a medical journal. The study participants will not be identified by name. After the study is completed, you may request an explanation of the study results and ask more information about the treatment you (or your child) received.

TREATMENT AND COMPENSATION FOR INJURY

If you are injured or have questions about injuries as a result of being in the study, please contact the doctors in the study clinic. The services at the public health facility will be open to you in case of any such injury.

QUESTIONS

This study has been explained to you by the person who signed below and your questions were answered.

JOINING OF YOUR OWN FREE WILL

PARTICIPATION IN RESEARCH IS VOLUNTARY. You and your child have the right to refuse to participate or to withdraw at any point in this study without penalty or loss of benefits to which you are otherwise entitled.

WHAT YOUR SIGNATURE OR THUMBPRINT MEANS

Your signature or thumbprint means that you understand the information given to you about your (or your child's) participation in the study and in this consent form. If you wish to participate, or wish for your child to participate in this study, you should sign or place your thumbprint below.

CONSENT FORM

CONSENT FORM FOR PARTICIPATION IN RESEARCH PROJECTS AND CLINICAL TRIALS

Study Title

A Phase III, randomized, non-inferiority trial, to assess the efficacy and safety of Dihydroartemisinin+Piperaquine (DHA+PPQ, Artekin) in comparison with Artesunate+Mefloquine (AS+MQ) in patients affected by acute, uncomplicated Plasmodium *falciparum* malaria.

- Multicentre Study In Asia -

Principal Investigator: ____

Address:____

Contact number____

I, …………………………………. mother/father/legally acceptable representative declare that I have understood the objectives and purposes of this study. I voluntarily agree that me or my child may participate in it.

____

Name of Participant (printed)

____

Signature or Fingerprint * of participant over 14 years   Date/Time

For children / adolescents:

____

Name of Parent/Guardian

____

Signature or Fingerprint * of Parent/Guardian   Date/Time

*If the patient, parent or guardian is unable to read and/or write, an impartial witness should be present during the informed consent discussion. After the written informed consent form is read and explained to the participant, parent or guardian, and after they have orally consented to their or their child's participation in the trial, and have either signed the consent form or provided their fingerprint, the witness should sign and personally date the consent form. By signing the consent form, the witness attests that the information in the consent form and any other written information was accurately explained to, and apparently understood by, the patient, parent or guardian, and that informed consent was freely given by the patient, parent or guardian.

____

Name of Person Witnessing Consent (printed)

____

Signature of Person Witnessing Consent Date/Time

APPENDIX V

Criteria for Severe Malaria/Danger Signs

Severe Malaria

- Unarousable coma *(if after convulsion, > 30 min)*
- Repeated convulsions *(> 2 within 24 h)*
- Severe anaemia *(Hb < 5.0 g/dL)*
- Respiratory distress *(laboured breathing at rest)*
- Jaundice *(yellow coloring of eyes)*

Danger Signs

- Recent convulsions *(1-2 within 24 h)*
- Altered consciousness *(confusion, delerium, psychosis)*
- Lethargy
- Unable to drink or breast feed
- Vomiting everything
- Unable to stand/sit due to weakness

APPENDIX VI. PARTICIPANT SELECTION AND ENROLLMENT

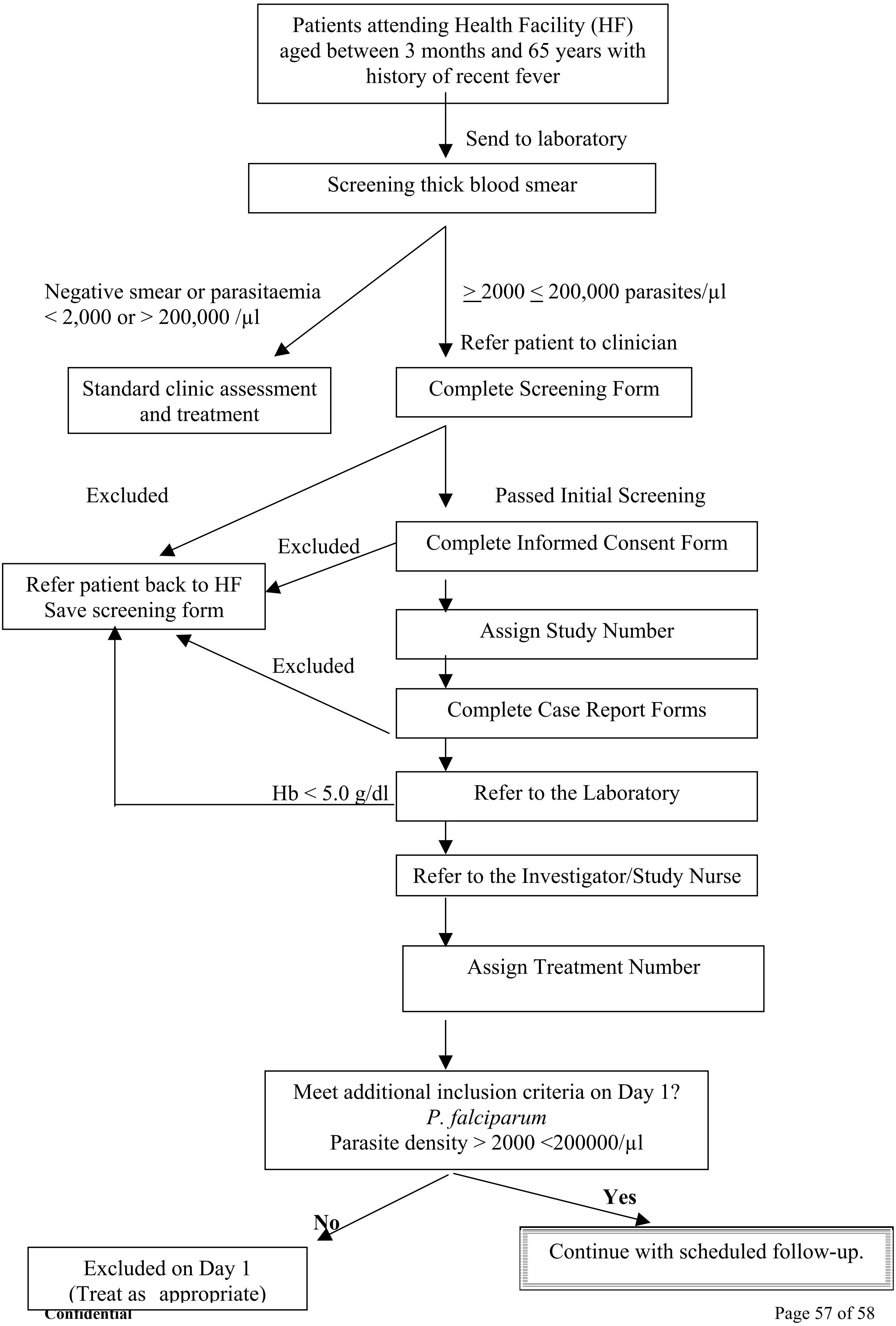

Supplement: Protocol S1 — Trial protocol. (1.32 MB PDF) [file pone.0011880.s002.pdf]
