# Supplementary material for: An Open-Label, Randomised Study of Dihydroartemisinin-Piperaquine Versus Artesunate-Mefloquine for Falciparum Malaria in Asia
Source: PLoS One. 2010 Jul 30;5(7):e11880. doi: 10.1371/journal.pone.0011880 (PMC2912766; doi:10.1371/journal.pone.0011880)
Supplement: Protocol S2 — Protocol amendment 1. (0.14 MB PDF) [file pone.0011880.s003.pdf]

Amendment N° : **1**

Protocol N° : **ST3073-ST3074 DM040010**

Title:

A Phase III, randomized, non-inferiority trial, to assess the efficacy and safety of Dihydroartemisinin+Piperaquine (DHA+PPQ, Artekin) in comparison with Artesunate+Mefloquine (AS+MQ) in patients affected by acute, uncomplicated *Plasmodium falciparum* malaria.

- MULTICENTRE STUDY IN ASIA-

Final protocol dated : **10 January, 2005**

Amendment N° 1 dated : **29 March 2005**

PURPOSE :

The main goal of this amendment is to correct duplications and clarify study procedures

MODIFICATION:
Page 2: Sites

Reason for modification : Increased number of sites involved in this study.

Therefore:

" 5 sites in Thailand, 1 in China, 1 in Laos ”

is modified as follows :

" **6** sites in Thailand, 1 in China, 1 in Laos”

MODIFICATION:
Page 3: Study Acknowledgment/ Confidentiality

Reason for modification: To add sentence regarding manuscript review.

Therefore:
However, to prevent premature disclosure of confidential information, the timing of a separate presentation or publication of the study by the Investigator will be subject to mutual agreement in advance between Sigma-Tau i.f.r. S.p.A., Pomezia (Rome) - Italy and the Investigator. Both parties will have an opportunity to review and comment on any manuscripts or abstracts arising from this study.

is modified as follows
However, to prevent premature disclosure of confidential information, the timing of a separate presentation or publication of the study by the Investigator will be subject to mutual agreement in advance between Sigma-Tau i.f.r. S.p.A., Pomezia (Rome) - Italy and the Investigator. Both parties will have an opportunity to review and comment on any manuscripts or abstracts arising from this study **within 3 months of presentation of the first draft**.

MODIFICATION:
Page 5: Synopsis Study Centres

Reason for modification: Increased number of study centres involved in this study.

Therefore:

" 5 sites in Thailand, 1 in China, 1 in Laos ”

is modified as follows :
" **6** sites in Thailand, 1 in China, 1 in Laos

MODIFICATION:
Page 21: Flow Chart.

Reason for modification : Gametocyte prevalence was not presented in the flow-chart but was mentioned in Section 3.8.

Page 21

Therefore:

"-"

is modified as follows :

"Gametocyte prevalence for D7, D14, D21, D28, D35, D42, D49, D56, D63 and Day of recurrent parasitaemia."

MODIFICATION:
Page 21: Flow Chart.

Reason for modification : Addition of Urinalysis on the Day of any recurrent parasitaemia.

Page 21

Therefore:

Urinalysis is not included for Day of any recurrent parasitaemia.

is modified as follows:

Urinalysis is marked in Day of any recurrent parasitaemia column.

Figure 1: Flow-chart

	Day 0 (pre-dose)	Day 1	Day 2	Day 3	Day 7	Day 14	Day 21	Day 28	Day 35	Day 42	Day 49	Day 56	Day 63	Day of any recurrent parasitemia
Visits	*V1*	*V2*	*V3*	*V4*(4)	*V5*	*V6*	*V7*	*V8*	*V9*	*V10*	*V11*	*V12*	*V13*	
**Demographic data/Medical history**	x													
**Informed consent recording**	x													
**Physical and Clinical examination**	x	x	x	x	x	x	x	x	x	x	x	x	x	x
**Vital signs and weight**(1)	x(1)	x	x	x	x	x	x	x	x	x	x	x	x	x
**Blood smear**(3) **(thick and thin)**	x	x	x	x	x	x	x	x	x	x	x	x	x	x
**Electrocardiogram (ECG)**	x		x					x					x	x
**Pregnancy test**	x													
**Haemoglobin/Haematocrit**	x				x	x	x	x	x	x	x	x	x	x
**Hematology/biochemistry**	x							x					x(2)	x
**Urinalysis**	x							x					x(2)	x
**Adverse events recording**	x	x	x	x	x	x	x	x	x	x	x	x	x	x
**PCR-sample**	x													x
**Gametocyte prevalence**					x	x	x	x	x	x	x	x	x	x

**Concomitant treatments**	x	x	x	x	x	x	x	x	x	x	x	x	x	x
**Study medications**	x	x	x											

1. *Weight will be measured at D0*
2. *Only if abnormal at D28*
3. *Daily until negative for asexual forms of parasite*
4. *Omit visit 4 if smear negative on visit 3 (day 2)*

MODIFICATION:
Page 22: Section 3.7 Selection of the patients 3.7.1 Inclusion criterion #3

Reason for modification : To reflect parameters used in Appendix 6

Therefore:

“ Microscopically confirmed, monoinfection of *Plasmodium falciparum* (asexual forms parasitaemia ≥ 5/500 WBC or mixed infection).”

is modified as follows:
“ Microscopically confirmed, monoinfection of *Plasmodium falciparum* (asexual forms parasitaemia **≥ 2000/µL ≤ 200,000/µL** or mixed infection).”

MODIFICATION:
Page 23: Section 3.8 Study procedures
Visit 1 (Day 0, pre-dose): screening visit/ administration of the study drug
Point #3 Physical and Clinical Examination

Reason for modification : To correct duplication. Vital signs and weight are specified in point #4.

Therefore:

“ A general physical examination will be performed (see Table B of Appendix I). Vital signs (Systolic and Diastolic Blood Pressure, Heart Rate) and Weight will be also collected. A clinical examination will be performed (see Appendix I): symptoms, hearing, aural temperature (electronic thermometer).”

is modified as follows:

“ A general physical and clinical examination will be performed (see Appendix I Tables **A, B&C). Aural temperature (electronic thermometer) will also be measured**.”

MODIFICATION:
Page 24-25: Section 3.8 Study procedures
Visits 2, 3 and 4

Reason for modification : To correct duplication. Vital Signs are specified in point #2.

Therefore:

“# 1 Physical and Clinical Examination, Vital Signs”

is modified as follows:

"#1 Physical and Clinical Examination"

MODIFICATION:
Page 25: Study procedure
Visits 5 to 7

Reason for modification : To specify the item "Haemoglobin/Haematocrit" in Section 3.8 as stated in the flow-chart page 21.

Therefore:

" Moreover, gametocyte prevalence will be evaluated at D7, D14, D21."

is modified as follows:

" Moreover, gametocyte prevalence **and fractional change in haemoglobin/haematocrit** will be evaluated at D7, D14, D21.

MODIFICATION:
Page 27: Section 3.12 Packaging and Labelling

Reason for modification : All medications will be dispensed to the patient by the clinic staff. The date dispensed will be recorded in the drug accountability form and in the CRF.

Therefore:

Each centre will receive a number of packages containing tablets of study drugs. Each package will be identified by a label indicating at least the following information:

- Product name
- Trial reference
- Name and address of the Sponsor
- Lot number and expiry date
- Storage directions
- Date dispensed
- Patient number

.

is modified as follows:

Each centre will receive a number of packages containing tablets of study drugs. Each package will be identified by a label indicating at least the following information:

- Product name
- Trial reference
- Name and address of the Sponsor
- Lot number and expiry date
- Storage directions

MODIFICATION:
Page 31: Section 6.2 Laboratory Evaluations

Reason for modification : To clarify correct volume of blood to be drawn.

Therefore:
" For laboratory analysis, a total volume of blood of about 6 ml will be drawn from each patient/child throughout the study.

is modified as follows:
"For laboratory analysis, a total volume of blood of about **12 ml (child) or 24 ml (adult) will be drawn from each patient** throughout the study.

MODIFICATION:
Page 33: Section 11 Investigator Responsibility

Reason for modification : To clarify any conflict of interest.

Therefore
"Except where the Principal Investigator's signature is specifically required, it is understood that the term "Investigator" as used in this protocol and on the CRFs refers to the Principal Investigator or a member of the staff that the Investigator designates to perform a certain duty under this protocol. The Investigator is ultimately responsible for the conduct of all aspects of the study.
For all other relevant Investigator responsibilities see "CPMP/ICH/135/95 Topic E6 - Guideline for Good Clinical Practice", Chapter 4."
"

is modified as follows
"Except where the Principal Investigator's signature is specifically required, it is understood that the term "Investigator" as used in this protocol and on the CRFs refers to the Principal Investigator or a member of the staff that the Investigator designates to perform a certain duty under this protocol. The Investigator is ultimately responsible for the conduct of all aspects of the study.
There is no conflict of interest between the Investigators and Sigma-Tau or MDS.
For all other relevant Investigator responsibilities see "CPMP/ICH/135/95 Topic E6 - Guideline for Good Clinical Practice", Chapter 4."
."

MODIFICATION:
Page 51: Appendix IV Research Participant Informed Consent
Procedures Point #8

Reason for modification : To clarify correct volume of blood to be drawn.

Therefore
"Each sample will be of 2 mL and will be collected from an arm vein by an experienced nurse."

is modified as follows
"Each sample will be **of 4 mL and 8 mL for children and adults respectively** and will be collected from an arm vein by an experienced nurse."
