# Supplementary material for: An Open-Label, Randomised Study of Dihydroartemisinin-Piperaquine Versus Artesunate-Mefloquine for Falciparum Malaria in Asia
Source: PLoS One. 2010 Jul 30;5(7):e11880. doi: 10.1371/journal.pone.0011880 (PMC2912766; doi:10.1371/journal.pone.0011880)

**Amendment N° :**	**2**
**Protocol N° :**	**ST3073-ST3074 DM040010**

Title:

A Phase III, randomized, non-inferiority trial, to assess the efficacy and safety of Dihydroartemisinin+Piperaquine (DHA+PPQ, Artekin) in comparison with Artesunate+Mefloquine (AS+MQ) in patients affected by acute, uncomplicated *Plasmodium falciparum* malaria.

- MULTICENTRE STUDY IN ASIA-

**Final protocol dated :**	**10 January, 2005**
**Revised protocol incorporating Amendment N°1 dated:**	**29 March, 2005**
**Amendment N° 2 dated:**	**14 April, 2005**

PURPOSE :

The main goals of this amendment are to accommodate routine medical practice at one of the sites, to clarify the number of participating sites, to decentralize the use of a central lab for blood chemistry and make some administrative changes.

MODIFICATION:
Page 2: Sites

Reason for modification: Increased number of sites involved in this study.

Therefore:

6 sites in Thailand, 1 in China, 1 in Laos

is modified as follows :

8 sites in Thailand, 1 in China, 1 in Laos

MODIFICATION:
Page 5: Synopsis Study Centres

Reason for modification: Increased number of study centres involved in this study.

Therefore:

6 sites in Thailand, 1 in China, 1 in Laos

is modified as follows :

8 sites in Thailand, 1 in China, 1 in Laos

MODIFICATION:
Page 5: Synopsis – Diagnosis and Main Inclusion Criteria

Reason for modification: Temperature will be recorded according to routine clinical practice at investigator's site. Sites are no longer restricted to taking tympanic temperature.

Therefore:

Males and Females aged between 3 months and 65 years inclusive, body weight at least 5 Kg, microscopically confirmed, monoinfection of Plasmodium *falciparum* or mixed infection, history of fever or presence of fever (tympanic temperature at ≥ 37.5 °C), written informed consent.

is modified as follows :

Males and Females aged between 3 months and 65 years inclusive, body weight at least 5 Kg, microscopically confirmed, monoinfection of *Plasmodium falciparum* or mixed infection, history of fever or presence of fever (temperature at ≥ 37.5 °C), written informed consent.

MODIFICATION:
Page 7: Synopsis – First Patient In

Reason for modification: To update the expected date.

Therefore:

April, 2005 (expected date)

is modified as follows :

June, 2005 (expected date)

MODIFICATION:
Page 17: Section 3.2 Primary Endpoint

Reason for modification: Temperature will be recorded according to routine clinical practice at investigator's site. Sites are no longer restricted to taking tympanic/axillary temperature.

Therefore:

The primary endpoint will be the PCR-corrected adequate clinical and parasitological response (PCR corrected ACPR) at D63.
ACPR is defined as the absence of parasitaemia on D63 irrespective of the tympanic temperature and not meeting any of the criteria of early treatment failure or late clinical or parasitological failure.

Patients classified as failures by clinical and parasitological criteria will be considered ACPR if the PCR analysis will show a new infection rather than a recrudescence.

The total treatment failure is defined according to the WHO criteria (WHO 2003) as the sum of early* and late** treatment failures.

* Early Treatment Failure (ETF)
(i) development of danger signs or severe malaria on Days 0, 1, 2 or 3, and the presence of parasitaemia,
(ii) parasitaemia on Day 2 > Day 0 count irrespective of axillary temperature,
(ii) parasitaemia on Day 3 with fever (temperature ≥ 37.5°C),
(iv) parasitaemia on Day 3 ≥ 25 % of count on Day 0.

is modified as follows :

The primary endpoint will be the PCR-corrected adequate clinical and parasitological response (PCR corrected ACPR) at D63.
ACPR is defined as the absence of parasitaemia on D63 irrespective of the temperature and not meeting any of the criteria of early treatment failure or late clinical or parasitological failure.

Patients classified as failures by clinical and parasitological criteria will be considered ACPR if the PCR analysis will show a new infection rather than a recrudescence.

The total treatment failure is defined according to the WHO criteria (WHO 2003) as the sum of early* and late** treatment failures.

* Early Treatment Failure (ETF)
(i) development of danger signs or severe malaria on Days 0, 1, 2 or 3, and the presence of parasitaemia,
(ii) parasitaemia on Day 2 > Day 0 count irrespective of temperature,
(ii) parasitaemia on Day 3 with fever (temperature ≥ 37.5°C),
(iv) parasitaemia on Day 3 ≥ 25 % of count on Day 0.

MODIFICATION:

Page 19: Section 3.3 Secondary Endpoints

Reason for modification: To clarify that weekly evaluation of fractional change is performed either for haemoglobin OR haematocrit.

Therefore:

- Fractional change in haemoglobin (Hb) and haematocrit.

The fractional change in haemoglobin/haematocrit will be evaluated weekly.

is modified as follows:

- Fractional change in haemoglobin (Hb) **or** haematocrit.

The fractional change in haemoglobin/haematocrit will be evaluated weekly.

MODIFICATION:

Page 20: Section 3.6 Flow Chart

Reason for modification: To accommodate routine medical practice at some centres.

Therefore

As part of routine practice in outpatient care facilities, all febrile patients have a thick blood smear for malaria before antimalarial treatment is administered. Patients attending the study health facility who have a positive blood smear for *P. falciparum* will be informed by the health facility staff about the malaria clinical trial and they will be requested to freely consent to participate in the trial. Those who decline to participate will receive the standard treatment for uncomplicated falciparum malaria**.**

Patients who accept to participate in the study will receive detailed explanations about the trial from the study staff. Specifically they will be informed that two antimalarial drugs are being tested and the option given to the patient will be decided randomly (by chance). They will be asked to sign the informed consent form (see Appendix VI for enrolment).

At enrolment, patients will be assigned a sequential study number. Patients will be managed as outpatients and treatment doses will be given under direct medical supervision.

Patients will be encouraged to return to the clinic for follow up assessment on days 1, 2, 3, 7, 14, 21, 28, 35, 42, 49, 56, 63 and any unscheduled day that they feel ill.

is modified as follows:

As part of routine practice in outpatient care facilities, all febrile patients have a thick blood smear for malaria before antimalarial treatment is administered. Patients attending the study health facility who have a positive blood smear for *P. falciparum* will be informed by the health facility staff about the malaria clinical trial and they will be requested to freely consent to participate in the trial. Those who decline to participate will receive the standard treatment for uncomplicated falciparum malaria**.**

Patients who accept to participate in the study will receive detailed explanations about the trial from the study staff. Specifically they will be informed that two antimalarial drugs are being tested and the option given to the patient will be decided randomly (by chance). They will be asked to sign the informed consent form (see Appendix VI for enrolment).

At enrolment, patients will be assigned a sequential study number. Patients will be managed as outpatients and treatment doses will be given under direct medical supervision. **At some centres patients can be hospitalised for at least 3 or 7 days according to local practice.**
Patients will be encouraged to return to the clinic for follow up assessment on days 1, 2, 3, 7, 14, 21, 28, 35, 42, 49, 56, 63 and any unscheduled day that they feel ill.

MODIFICATION:

Page 21: Figure 1 Flow Chart.

Reasons for modification:

i. Blood smear (thick and thin) will be performed on every visit irrespective of results.
ii. **Additional** ECG to be performed at Day 7.
iii. To clarify window period for Day 63.
iv. Inclusion/Exclusion Checklist is specified in Section 3.8 Procedures for Visit 1 (Day 0) but not included in the flow chart.

Therefore:

Figure 1: Flow-chart

	Day 0 (pre-dose)	Day 1	Day 2	Day 3	Day 7	Day 14	Day 21	Day 28	Day 35	Day 42	Day 49	Day 56	Day 63	Day of any recurrent parasitemia
Visits	*V1*	*V2*	*V3*	*V4(4)*	*V5*	*V6*	*V7*	*V8*	*V9*	*V10*	*V11*	*V12*	*V13*	
Demographic data/Medical history	x													
Informed consent recording	x													
Physical and Clinical examination	x	X	x	x	x	x	x	x	x	x	x	x	x	x
Vital signs and weight(1)	x(1)	X	x	x	x	x	x	x	x	x	x	x	x	x
Blood smear(3) (thick and thin)	x	X	x	x	x	x	x	x	x	x	x	x	x	x
Electrocardiogram (ECG)	x		x		x								x	x
Pregnancy test	x													
Haemoglobin/Haematocrit	x				x	x	x	x	x	x	x	x	x	x
Hematology/biochemistry	x							x					x(2)	x
Urinalysis	x							x					x(2)	x
Adverse events recording	x	X	x	x	x	x	x	x	x	x	x	x	x	x
PCR-sample	x													x
Gametocyte prevalence					x	x	x	x	x	x	x	x	x	x
Concomitant treatments	x	X	x	x	x	x	x	x	x	x	x	x	x	x
Study medications	x	X	x											

1. *Weight will be measured at D0*
2. *Only if abnormal at D28*
3. *Daily until negative for asexual forms of parasite*
4. *Omit visit 4 if smear negative on visit 3 (day 2)*

is modified as follow:

i. Superscripts #3 and #4 are removed from 'Blood smear (thick and thin)' and from 'V4' respectively. Footnotes #3 'Daily until negative for asexual forms of parasite' and #4 'Omit visit 4 if smear negative on visit 3 (day 2)' are deleted.
ii. ECG is marked for Day 7 on the flow chart. An ECG is to be repeated at Day 28 only if abnormal at Day 7 and at Day 63 if abnormal at Day 28.
iii. Footnotes #5 'Only if abnormal at D7' and #6 'Only if abnormal at D28' are moved up to #3 and #4 respectively.
iv. Superscript #5 is added to 'Day 63' along with corresponding explanation in the footnote.
v. Inclusion/Exclusion Checklist is marked for Day 0 on the flow chart.

Figure 2: Flow-chart

	**Day 0 (pre-dose)**	**Day 1**	**Day 2**	**Day 3**	**Day 7**	**Day 14**	**Day 21**	**Day 28**	**Day 35**	**Day 42**	**Day 49**	**Day 56**	**Day 63(5)**	**Day of any recurrent parasitemia**
Visits	***V1***	***V2***	***V3***	***V4***	***V5***	***V6***	***V7***	***V8***	***V9***	***V10***	***V11***	***V12***	***V13***	
**Demographic data/Medical history**	x													
**Informed consent recording**	x													
**Physical and Clinical examination**	x	X	x	x	x	x	x	x	x	x	x	x	x	x
**Vital signs and weight(1)**	x(1)	X	x	x	x	x	x	x	x	x	x	x	x	x
**Blood smear (thick and thin)**	x	X	x	x	x	x	x	x	x	x	x	x	x	x
**Electrocardiogram (ECG)**	x		x		x			x(3)					x(4)	x
**Pregnancy test**	x													
**Haemoglobin/Haematocrit**	x				x	x	x	x	x	x	x	x	x	x
**Hematology/biochemistry**	x							x					x(2)	x
**Urinalysis**	x							x					x(2)	x
**Adverse events recording**	x	X	x	x	x	x	x	x	x	x	x	x	x	x
**PCR-sample**	x													x
**Gametocyte prevalence**					x	x	x	x	x	x	x	x	x	x
**Concomitant treatments**	x	X	x	x	x	x	x	x	x	x	x	x	x	x
**Inclusion/Exclusion Checklist**	x													
**Study medications**	x	X	x											

1. *Weight will be measured at D0*
2. *Only if abnormal at D28*
3. *Only if abnormal at D7*
4. *Only if abnormal at D28*
5. *Day 63(-1/+7)*

MODIFICATION:

Page 22: Section 3.7 Selection of the patients 3.7.1 Inclusion criterion #3

Reason for modification: To correct lower limit of parasitaemia

Therefore:

Microscopically confirmed, monoinfection of *Plasmodium falciparum* (asexual forms parasitaemia ≥ 2000/µL ≤ 200,000/µL or mixed infection).

is modified as follows:

Microscopically confirmed, monoinfection of *Plasmodium falciparum* (asexual forms parasitaemia **≥ 80/µL** ≤ 200,000/µL or mixed infection).

MODIFICATION:

Page 22: Section 3.7 Selection of the patients 3.7.1 Inclusion criterion #4

Reason for modification : Temperature will be recorded according to routine clinical practice at investigator's site. Sites are no longer restricted to taking tympanic temperature.

Therefore:

History of fever or presence of fever (tympanic temperature at ≥ 37.5 °C).

is modified as follows:

History of fever or presence of fever (temperature at ≥ 37.5 °C).

MODIFICATION:

Page 23-25 Section 3.8 Study Procedure
Physical and Clinical Examination

Reason for modification: To clarify how body temperature will be measured

Therefore:

A general physical examination and a clinical examination will be performed: symptoms, aural temperature (electronic thermometer).

is modified as follows:

A general physical examination and a clinical examination will be performed. **See Appendix I, Tables A, B & C. Temperature will be recorded according to routine clinical practice at investigator's site.**

MODIFICATION:

Page 24: Section 3.8 Study Procedure
Visit 1 (Day 0, pre-dose)

Reason for modification : To include concomitant treatments and adverse events recording as specified in the flow chart

Therefore:

9. Inclusion/Exclusion Checklist
   All inclusion and exclusion criteria must be met before enrolment in the study.

10. Administration of the study drugs

11. Pregnancy test
In women of child bearing age (unless menstruating), a urine test for β-HCG will be performed

is modified as follows:

9. Concomitant Pharmacological Treatments
Prior and concomitant medications taken by the patients will be recorded. For a list of allowed and disallowed medications, see section 4.

10. Adverse Event Report
The appearance of adverse events during the visit will be recorded.

11. Pregnancy test
In women of child bearing age (unless menstruating), a urine test for β-HCG will be performed

12. Inclusion/Exclusion Checklist
All inclusion and exclusion criteria must be met before enrolment in the study.

13. Administration of the study drugs

MODIFICATION:
Page 25 Section 3.8 Study Procedure
Visits 5, 6, 7 (Days 7, 14, 21)

Reason for modification: To include ECG done on D7 as specified in the flow chart

Therefore:

During these visits, the same procedures as the visit 4 will be applied.

Moreover, gametocyte prevalence and fractional change in haemoglobin/haematocrit will be evaluated at D7, D14, D21.

is modified as follows:

During these visits, the same procedures as the visit 4 will be applied. **For Visit 5 (D7), ECG will also be performed.**

Moreover, gametocyte prevalence and fractional change in haemoglobin/haematocrit will be evaluated at D7, D14, D21.

MODIFICATION:
Page 26 Section 3.8 Study Procedure
Visits 9, 10, 11, 12 (Days 35, 42, 49, 56)

Reason for modification: To clarify that ECG will not be performed on these visits.

Therefore:

During these visits, the same procedures as the visit 5 will be applied.

is modified as follows:

During these visits, the same procedures as the visit 5 will be applied **but ECG will not be performed**.

MODIFICATION:
Page 26 Section 3.8 Study Procedure
Visit 13 (Day 63)

Reason for modification: To include window period for Visit 13 (Day 63) as specified in the flow chart

Therefore:

During this visit, the same procedures as the visit 8 will be applied.

is modified as follows:

During this visit, the same procedures as the visit 8 will be applied. **This visit can occur minus 1 or plus 7 days of the scheduled visit.**

MODIFICATION:
Page 29 Section 5 Patient Withdrawal Criteria

Reason for modification: To re-define the withdrawal criterion for lost to follow-up patients and to add one more reason to the withdrawal criteria.

Therefore:

During the study, the following conditions are reasons for excluding the patients from further participation in the trial:

1. Use of antimalarial drugs outside of the study protocol.
2. Withdrawal of informed consent.
3. Lost to follow-up: patients who fail to attend a follow-up visit and are unable to be located within 48 hours on Days 1-14 or for more than 2 subsequent visits between Days 15-63.
4. Patient's request to discontinue for any reason.
5. At the investigator's request for safety reasons

is modified as follows:

During the study, the following conditions are reasons **for stopping the study treatment and/or for excluding the patients from further efficacy assessments:**

1. **Any treatment failure (including early failures).**
2. Use of antimalarial drugs **(or antibiotics with antimalarial activity)** outside of the study protocol.
3. **Persistent vomiting of study drugs on Day 0 (at least twice the same dose).**
4. **Failure to complete the study treatment.**
5. Withdrawal of informed consent.
6. Investigator's request for safety reason.
7. **Lost to follow-up: patients cannot be located by Day 63.**

Patients with one or more of the conditions listed above will have to be followed-up for the safety assessments until day 63. These patients will be considered failures in the Intention To Treat population (see section 8.1) and, with exclusion of condition 1, they will be excluded from the Evaluable population (see again section 8.1).

<u>MODIFICATION:</u>
Page 31 Section 6.1.4 Reporting of adverse events

Reason for modification: To clarify that all adverse events regardless of severity, should be reported and to clarify the time window for reporting the adverse events.

Therefore:

For all adverse events identified, an adverse event report form will be completed.

For each possible adverse event identified and graded as moderate, severe, an adverse event report form will be completed. An adverse event report form will not be completed for events classified as mild as these symptoms are common and difficult to distinguish from signs and symptoms due to malaria.

is modified as follows:

For all adverse events identified **between signature of the informed consent and Day 63**, an adverse event report form will be completed.

<u>MODIFICATION:</u>
Page 31 Section 6.2 Laboratory Evaluations

Reason for modification: Biochemistry samples will no longer be sent to MDS Pharma Services central laboratory. The blood chemistry assessment will be performed locally at sites.

Therefore:

Blood samples will be properly labelled with patients' initials, randomisation number, protocol number, and the date the sample is taken. Haematology assessments will be performed locally at sites. Blood chemistry tests will be sent to MDS Pharma Services central laboratory.

is modified as follows:

Blood samples will be properly labelled with patients' initials, randomisation number, protocol number, and the date the sample is taken. Haematology and blood chemistry assessments will be performed locally at sites.

MODIFICATION:
Page 32 Section 8.1 Populations analysed

Reason for modification: To clarify the composition of the populations to be considered for the statistical analysis.

Therefore:

The following populations will be considered for the statistical analysis.

Intention-to-Treat (ITT) Population: Defined as all randomized patients who will take at least one dose of the study treatment.
Evaluable Population (or Per-Protocol (PP) Population): Defined as all randomized patients who will be eligible according to the study protocol, will take at least 80% of the study medication, will not take other anti-malarial drugs and, if presence of malarial symptoms or signs, will have an evaluable PCR.

is modified as follows:

The following populations will be considered for the statistical analysis **of efficacy**.

Intention-to-Treat (ITT) Population: Defined as all randomized patients who will take at least one dose of the study treatment. **All patients who withdrew from the study for one of the reasons listed in section 5 will be evaluated as failures in this population.**
Evaluable Population (or Per-Protocol (PP) Population): Defined as all randomized patients who will be eligible according to the study protocol, will take **all** the study medication, **will be failures or will have the Day 63 assessment, will not take anti-malarial drugs outside of the study protocol, will have enough follow-up data (i.e. will not have violations 3 and 4 listed under section 16)** and, in presence of **recurrent parasitaemia** will have an evaluable PCR.

MODIFICATION:
Page 33 Section 8.2 Efficacy: primary analysis

Reason for modification: Remove a statement that may create conflict with the description of the analysis populations given in section 8.1 and remove a typo error.

Therefore:

The primary analysis will be based on a 97.5% (one-sided) Confidence Interval (CI) computed on the difference between the 63-day PCR corrected cure rates of the test and the reference treatments, respectively.
In order to claim that DHA+PPQ is efficacious, the lower limit of the CI must be > -0.05 and the point estimate for the 63-day PCR corrected cure rate of DHA+PPQ must be > 0.90, in both the ITT and the Evaluable populations.
The method for computing the CI will be specified in the SAP.
All patients who withdrew from the study for one of the reasons listed in section 5 will be evaluated as failures in the ITT population and, if they qualify as Evaluable patients, also in the Evaluable population.

For computing the PCR-corrected cure rate, the following rules will be applied:

- the patients who are diagnosed to be re-infected are counted as successes (they have to be terminated on that day of follow-up);
- the patients for whom the PCR is not interpretable or is missing are counted as failures in the ITT population, while they are excluded from the Evaluable population. The proportion of these patients is less than 10% of patients who experienced a re-infection (i.e. those in whom the PCR evaluation is performed).

is modified as follows:

The primary analysis will be based on a 97.5% (one-sided) Confidence Interval (CI) computed on the difference between the 63-day PCR corrected cure rates of the test and the reference treatments, respectively.
In order to claim that DHA+PPQ is efficacious, the lower limit of the CI must be > -0.05 and the point estimate for the 63-day PCR corrected cure rate of DHA+PPQ must be > 0.90, in both the ITT and the Evaluable populations.
The method for computing the CI will be specified in the SAP.
For computing the PCR-corrected cure rate, the following rules will be applied:

- the patients who are diagnosed to be re-infected are counted as successes (they have to be terminated on that day of follow-up);
- the patients for whom the PCR is not interpretable or is missing are counted as failures in the ITT population, while they are excluded from the Evaluable population. The proportion of these patients is less than 10% of patients who experienced a **recurrent parasitaemia** (i.e. those in whom the PCR evaluation is performed).

MODIFICATION:
Page 36 Section 16 Protocol Violation

Reason for modification: To re-define the criteria for protocol violation

Therefore:

A protocol violation occurs when a study patient is removed from the study because of an event that does not allow for continued accurate interpretation of response to treatment.
Patients meeting any of the following criteria will be withdrawn from follow-up:

- withdrawal of consent,
- failure to complete the treatment,
- persistent vomiting of study drugs on day 0,
- severe side effects necessitating hospitalisation,
- PCR unclassifiable results,
- Self-medication with antimalarial (or antibiotics with antimalarial activity),
- Severe malaria at Day 0,
- failure to attend enough of the scheduled visits,
- loss to follow-up

is modified as follows:

A protocol violation occurs when **an event happens that does not allow for accurate interpretation of response to treatment.**
Patients meeting any of the following criteria will be **considered protocol violators:**

1. **violation of any eligibility criteria (see section 3.7);**
2. PCR unclassifiable **or missing** results;
3. **failure to attend visits between Days 0 – 2 and unable to be located within 6 hours of the scheduled visits;**
4. **failure to attend two consecutive visits between Days 3 and 63 (every visit between Day 3 and Day 56 must take place within 48 hours of the scheduled time; visit at Day 63 can occur minus 1 or plus 7 days of the scheduled visit);**

The violations listed above are not valid reasons for withdrawing the patients from follow-up (both efficacy and safety assessments). On the contrary, the Investigator must do his/her best to respect the allowed time windows at every visit, even if a violation occurred at a previous visit (i.e. a previous visit did not take place or occurred outside the allowed time window).
The protocol violations listed above will not taken into account for establishing patient inclusion in the Intention-To-Treat population (see section 8.1) while they will be a reason for excluding the patients from the Evaluable population (see again section 8.1).

MODIFICATION:
Page 41:Table A. Guidelines for Grading Patient Symptoms

Reason for modification: Grade 4 description for behavioural changes not legible.

Therefore, within the table:

Toxic psychosis; □nrolment□ation required

is modified as follows:

Toxic psychosis; **hospitalization for treatment**

MODIFICATION:
Page 43:Table C. Grading Physical Examination Findings

Reason for modification: Temperature will be recorded according to routine clinical practice at investigator's site. Sites are no longer restricted to taking axillary temperature.

Therefore, within the table:

Temperature
(axillary)

is modified as follows:

Temperature

MODIFICATION:
Page 53:Appendix IV Research Participant Informed Consent Form
How The Study Is Done, point #3

Reason for modification: To re-define the reason for discontinuing study participation

Therefore:

If we are unable to locate you (or your child) within 48 hours on Days 1-14 or for more than 2 subsequent visits between Days 15-63.

is modified as follows:

If we are unable to locate you (or your child) by Day 63.

MODIFICATION:
Page 53:Appendix IV Research Participant Informed Consent Form
Procedures

Reason for modification:
i. To add the statement that patient will be hospitalized for at least 3 or 7 days depending on the site's local practice.
ii. To include urinalysis, ECG, urine pregnancy test

Therefore, this section is modified as follows:

1) The study doctors will examine you (or your child) today. **If it is the clinic's local practice, you (or your child) will be required to stay at the clinic for at least 3 and up to 7 days**.
2) A blood sample will be collected. A small amount of blood will be taken by fingerprick to examine for malaria parasites, to measure the haematocrit, to store blood samples on filter paper for future laboratory tests that will not impact on the health care of you (or your child).
3) If you (or your child) are eligible for the study, treatment with dihydroartemisinin + piperaquine (DHA+PPQ) or artesunate + mefloquine (AS+MQ) will be given.
4) You will be asked to return to the clinic at least 12 more times over the next 2 months so that the success of the treatment can be judged. At each of the follow-up visits, you (or your child) will be examined by the study doctors and, a small amount of blood will taken by fingerprick to examine for malaria parasites.
5) If you (or your child) miss an appointment, the home health visitor will visit you at your home to find out why you missed the appointment and bring you (or your child) to the clinic for assessment.
6) If, at any time, the treatment given to you (or your child) does not seem to be working well, it will be changed to treatment according to the usual standard of care.
7) There will be someone at the study clinic every day. You (or your child) can come to the clinic for evaluation anytime that you (or your child) are ill during the next 63 days.
8) For the haematology and biochemistry, there will be 2-3 blood samples: one before the first dose at D0 (Visit 1), the second one at D28 (Visit 8), i.e. one month after D0, and the last one at D63 (Visit 13) in case of abnormality at D28. Each sample will be of 4 mL and

8 mL for children and adults respectively and will be collected from an arm vein by an experienced nurse. Blood sampling may cause pain and swelling. **Urine tests will also be performed on the same visits.**

9) **You will have an ECG performed to test for heart function on D0 (Visit 1), D2 (Visit 3) and D7 (Visit 5). This test will be repeated on D28 (Visit 8) and on D63 (Visit 13) if results are abnormal on D7 and D28 respectively.**
10) **If you are female of childbearing age, a urine pregnancy test will be performed before you start the treatment. You are not able to participate in this study if you are pregnant or lactating.**

MODIFICATION:
Page 54:Appendix IV Research Participant Informed Consent Form
Confidentiality

Reason for modification: To remove this paragraph from 'Risks and Discomforts' and place it under a separate heading.

Therefore:

6) Confidentiality: Information about you (or your child) will be handled as confidentially as possible. Medical information related to malaria will be collected on your child, but only the people working on the study will see it. Anyone assigned to review this study will be granted direct access to your child's medical records, if necessary, for verification of the study procedures and data. Records will be kept as confidential as possible.

is modified as follows:

CONFIDENTIALITY

Information about you (or your child) will be handled as confidentially as possible. Medical information related to malaria will be collected on **you (or your child),** but only the people working on the study will see it. Anyone assigned to review this study will be granted direct access to **your (or your child's)** medical records, if necessary, for verification of the study procedures and data. Records will be kept as confidential as possible.

<u>MODIFICATION:</u>
Page 59 : Appendix VI PARTICIPANT SELECTION AND ENROLLMENT

Reason for modification: To correct lower limit of parasitaemia as reflected in Inclusion criterion #3

Therefore:

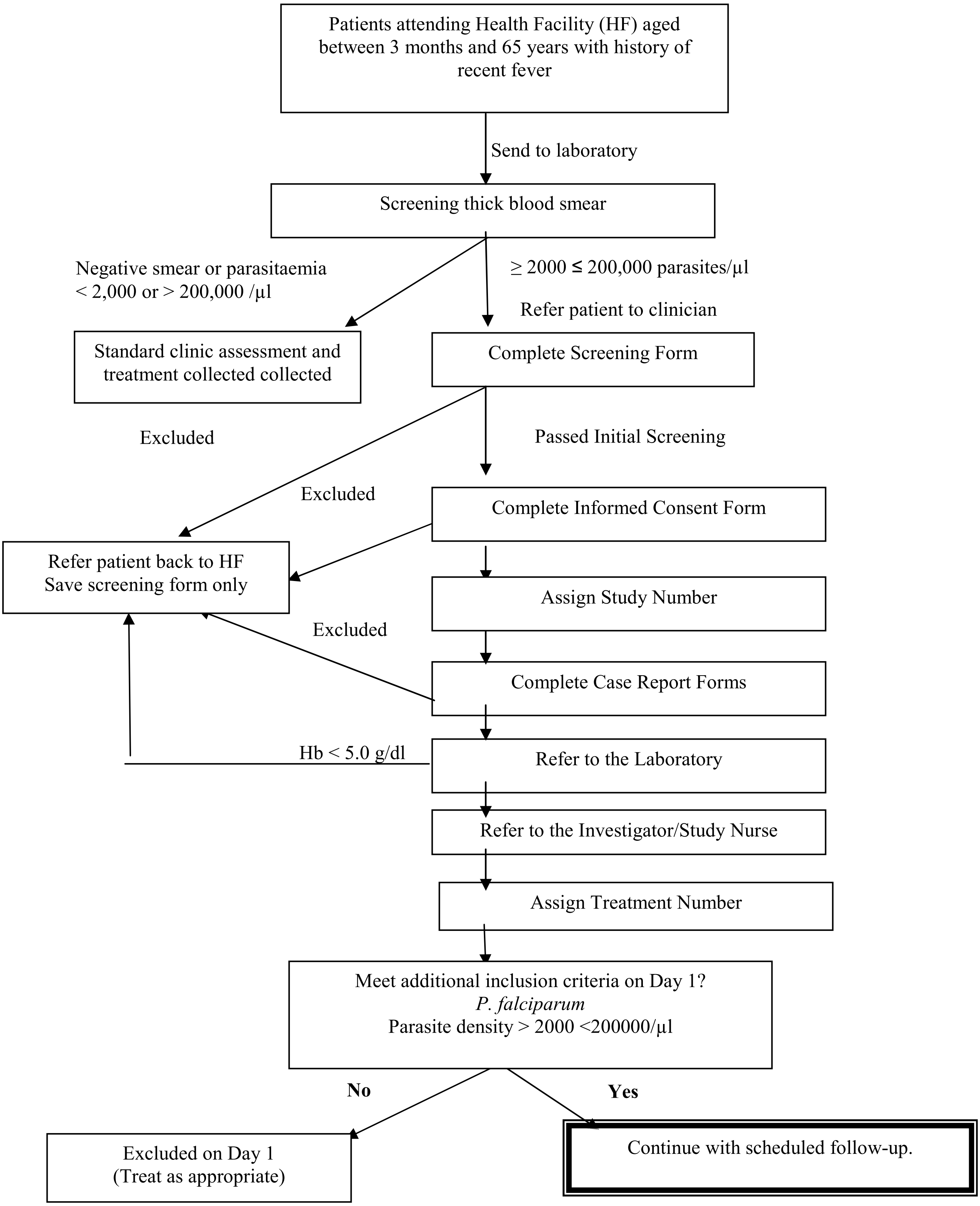

is modified as follows:

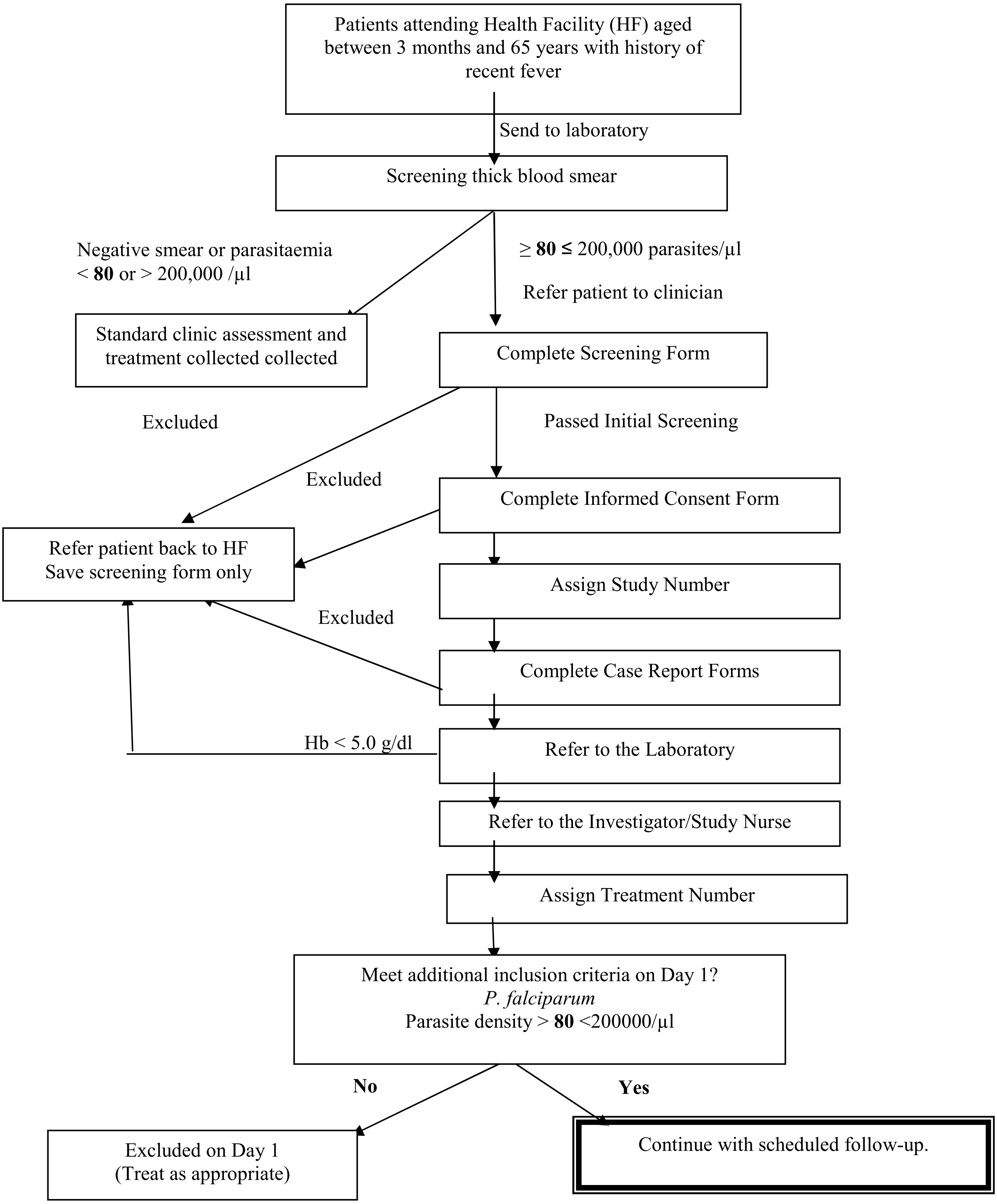

Supplement: Protocol S3 — Protocol amendment 2. (0.23 MB PDF) [file pone.0011880.s004.pdf]
