# Supplementary material for: An Open-Label, Randomised Study of Dihydroartemisinin-Piperaquine Versus Artesunate-Mefloquine for Falciparum Malaria in Asia
Source: PLoS One. 2010 Jul 30;5(7):e11880. doi: 10.1371/journal.pone.0011880 (PMC2912766; doi:10.1371/journal.pone.0011880)
Supplement: Protocol S4 — Protocol amendment 3. (0.19 MB PDF) [file pone.0011880.s005.pdf]

Amendment N°: **3**

Protocol N°: **ST3073-ST3074 DM040010**

Title:

A Phase III, randomized, non-inferiority trial, to assess the efficacy and safety of Dihydroartemisinin+Piperaquine (DHA+PPQ, Artekin) in comparison with Artesunate+Mefloquine (AS+MQ) in patients affected by acute, uncomplicated *Plasmodium falciparum* malaria.

- MULTICENTRE STUDY IN ASIA-

**Final protocol dated :**	**10 January, 2005**
**Revised protocol incorporating Amendment N° 1 & 2 dated:**	**14 April 2005**
**Amendment N° 3 dated:**	**24 November 2005**

PURPOSE:

The present study is part of a clinical development programme of Artekin which includes another phase III study to be conducted in Africa.

The proposed amendment has been formulated in response to specific recommendations of the Data Monitoring Committee (DMC) of the study, as described in the minutes of the first meeting of this Committee.

- The DMC made the recommendation that the Sponsor should harmonize the statistical approach for the African and the Asian studies.
- The DMC raised some concern about the second primary endpoint 'the cure rate of Artekin must be at least 90%'.

According to these recommendations, the following changes are proposed:

  - re-formulation of the primary objective;
  - inclusion of a new patient population, i.e. the modified intention-to-treat population;
  - inclusion of an interim analysis for the sample size re-assessment.

Other changes proposed in the present amendment are minor corrections of the protocol, and the notification that, in addition to the Steering Committee and the DMC, a third Committee (i.e. the Clinical Development Committee) has been constituted with the responsibility of evaluating the recommendations made by the other Committees and possibly transforming them in final decisions.

MODIFICATION:
Page 5: Synopsis Countries
Reason for modification: China will no longer be participating in the study

Therefore:

3 countries: Thailand, China and Laos.

is modified as follows :

2 countries: Thailand, and Laos

MODIFICATION:
Page 5: Synopsis Study Centers
Reason for modification: China will no longer be participating in the study and the number of sites has increased in Laos

Therefore:

8 sites in Thailand, 1 site in China and 1 in Laos

is modified as follows :

8 sites in Thailand and 2 in Laos

MODIFICATION:
Page 5: Synopsis Methodology
Reason for modification: There is no stratification in the study. The synopsis reflects an old version of the protocol

Therefore:

A phase III, randomized, open label, two arms study.
Randomization will stratify infants, children and adults by age.

is modified as follows :

A phase III, randomized, open label, two arms study.

MODIFICATION:

Page 5: Synopsis Objective (Primary objective)

Reason for modification: This change has been proposed to reflect the new formulation of the primary objective (see below).

Therefore:

The primary objective of the study is to measure the Day 63, PCR corrected cure rates of Artekin and AS+MQ and demonstrate that:

- o the cure rate of Artekin is non-inferior to that of AS+MQ (non-inferiority margin = 5%);
- o the cure rate of Artekin is at least 90%.

This cure rate is defined as the proportion of patients with adequate clinical and parasitological response at Day 63 plus those treatment failures identified as new infection by PCR.

is modified as follows :

The primary objective of the study is to measure the Day 63, PCR corrected cure rates of Artekin and AS+MQ and demonstrate that the cure rate of Artekin is non-inferior to that of AS+MQ (non-inferiority margin = 5%). This cure rate is defined as the proportion of patients with adequate clinical and parasitological response at Day 63 plus those treatment failures identified as new infection by PCR.
In order to compare the study results with the historical ones, the criteria described in the WHO document (“Assessment and Monitoring of Antimalarial Drug Efficacy for the Treatment of Uncomplicated Falciparum Malaria – 2003”. See chapter 7, section 7.3) will be used.

MODIFICATION:

Page 18: Section 2.1 – Primary objective

Reason for modification: This change reflects the need to clearly distinguish the two objectives of proving non-inferiority on one side and comparing the study results with the historical ones on the other side. It also clarifies some aspects of the historical comparisons.
The requirement of a cure rate higher than a pre-specified threshold for only one of the two treatments under comparison was in contradiction with the comparative nature of the trial. Suppose that at the end of the study the two cure rates were 85% and 87% for the AS+MQ and the Artekin groups, respectively. What could the conclusion be from the study according to the old formulation of the primary objective?
As declared in the WHO document described above, the cure rate threshold is intended for judging the results of single arm studies, aimed at the development of malaria policies, but not at interpreting results of comparative studies.
Of course, in non-inferiority studies there is the problem of showing the external validity of the trial: in fact, the equivalence (or non-inferiority) of the treatments under comparison could be theoretically shown at a level of non efficacy (see ICH guideline E10). To this extent, the comparison of the study results with historical data is very important. However, this comparison should involve both treatment groups.
Furthermore, the potential “confounding” factors that could impact the interpretation of results from trials with concurrent randomized arms are different from the ones that can

impact the interpretation of historical comparisons: in the former case the primary focus is on bias and variability, while in the latter it is on reproducibility of the experimental setting; in the former case the decision rule is of a black or white nature while in the second the efficacy threshold has the role of a "rule of thumb" (see WHO document). Therefore, the statistical techniques suited for the two analyses are different (see statistical section below).
For these reasons, the two objectives of showing non-inferiority of Artekin vs AS+MQ on one side and comparing the study results with the historical ones on the other side have been described as two separate and well distinguished objectives.

Therefore:

The primary objective of the study is to measure the Day 63, PCR corrected cure rates of Artekin and AS+MQ and demonstrate that:

- the cure rate of Artekin is non-inferior to that of AS+MQ (non-inferiority margin = 5%);
- the cure rate of Artekin is at least 90%.

This cure rate is defined as the proportion of patients with adequate clinical and parasitological response at Day 63.

is modified as follows :

The primary objective of the study is to measure the Day 63, PCR corrected cure rates of Artekin and AS+MQ and demonstrate that the cure rate of the former treatment is non-inferior to that of the latter (non-inferiority margin = 5%). This cure rate is defined as the proportion of patients with adequate clinical and parasitological response at Day 63 plus those treatment failures identified as new infection by PCR.

In order to compare the study results with the historical ones, the criteria described in the WHO document ("Assessment and Monitoring of Antimalarial Drug Efficacy for the Treatment of Uncomplicated Falciparum Malaria – 2003". See chapter 7, section 7.3) will be used, i.e. the failure rates computed in each treatment group individually considered will be judged against the efficacy threshold of 90%.

MODIFICATION:
Page 20: Section 3.4 – Sample size
Reason for modification: The rewording of the first section reflects the new formulation of the primary objective. The inclusion of the interim analysis for sample size re-assessment reflects the need of verifying the assumptions on which the sample size computation has been based, given the high impact on the final figures that even small changes in the assumptions can have and the limitation of the available data.

Therefore:

The study is designed as a non-inferiority trial.
The level of the PCR-corrected cure rate at day 63 for Artesunate+Mefloquine has been estimated, through a literature search, to be in the range 92%-95%.
The opinion of physicians and experts in malaria treatment has been sought for defining the non-inferiority margin, and this has been set at 5%. In addition, the experts have agreed that,

in order for the new treatment to be considered efficacious in Asia, its PCR-corrected cure rate at day 63 should not be lower than 90%. Therefore, in the present trial, DHA+PPQ will be considered efficacious if both the following conditions are met (see section 2.1):
(i) the PCR-corrected cure rate at day 63 of DHA+PPQ is not inferior to the cure rate of AS+MQ by 5% or more, and independently of the reference drug performance,
(ii) the cure rate of DHA+PPQ is at least 90%.

In summary, for the non-inferiority analysis, the clinical and statistical specifications for the sample size computations are as follows:

- Primary endpoint = PCR-corrected cure rate at day 63.
- Primary analysis = based on 97.5% one-sided confidence intervals for the difference in cure rates.
- Alpha = 0.025 (one-sided).
- Power = 80%.
- Cure rate of Artesunate+Mefloquine = at least 92%.
- Non-inferiority margin for the difference (test-reference) = -0.05.
- Randomization: based on a 2 :1 allocation scheme (test : reference).
- Rate of patient attrition in the Evaluable Population (i.e. rate of withdrawals and protocol violations) = 15%.
- Primary Analysis Populations = both the Intention To Treat (ITT) and the Evaluable Populations.

A simulation was performed for estimating the overall power that this study would have for addressing both of its primary objectives. That power was computed for both the lower bound of a 97.5 one-sided confidence interval for the treatment difference being above –0.05 and the point estimate for the test treatment being at least 90%. The scenario in which both treatments have cure rates of 90% has the contra-indicating limitation of a power of 50% for the point estimate for the test treatment to exceed 0.90, no matter how large the sample size is (in fact, if the true success rate is 90% for the test treatment, then 50% of studies will have an observed success rate > 90% and 50% of studies will have an observed success rate < 90%). Thus, one inherently needs to make the assumption that both cure rates are at least 91% in order to identify a realistic sample size with acceptable power.
Details on the above mentioned simulation are provided in a separate document (see Sample Size and Design Justification for The Pivotal Clinical Trials of Dihydroartemisinin+ Piperaquine).
Based on the above assumptions, the recommended sample size is 350 in the Artesunate+Mefloquine arm and 700 in the Dihydroartemisinin+Piperaquine arm (1050 patients in total).
The sample size of 1050 patients will concern the ITT population. For both the test and the reference compounds, the cure rates are expected to be higher in the Evaluable population as compared to the ITT population. Therefore, even if the Evaluable population is smaller than the ITT population, no impact on sample size is expected.
An unequal randomization with 2:1 structure was chosen because it assures a larger sample size for the DHA+PPQ group which provides the following advantages:

- a more precise estimate of the DHA+PPQ cure rate;
- a bigger sample for the integrated safety data base of DHA+PPQ, that renders more likely the elucidation of rare adverse reactions, if any.

From an ethical point of view, this choice appears reasonable in view of the previous data on DHA+PPQ, in terms of both safety and efficacy

is modified as follows:

3.4.1 Sample Size Computation

The study is designed as a non-inferiority trial.
The level of the PCR-corrected cure rate at day 63 for Artesunate+Mefloquine has been estimated, through a literature search, to be in the range 92%-95% in the Intention-To-Treat.(ITT) population. The opinion of physicians and experts in malaria treatment has been sought for defining the non-inferiority margin, and this has been set at 5%.
An unequal randomization with 2:1 structure was chosen because it assures a larger sample size for the DHA+PPQ group which provides the following advantages:

- a more precise estimate of the DHA+PPQ cure rate;
- a bigger sample for the integrated safety data base of DHA+PPQ, that renders more likely the elucidation of rare adverse reactions, if any.

From an ethical point of view, this choice appears reasonable in view of the previous data on DHA+PPQ, in terms of both safety and efficacy**.**
Sample size was initially (see original version of the protocol) computed considering the original formulation of the primary objective, which required the use of a simulation (details are provided in a separate document - see Sample Size and Design Justification for The Pivotal Clinical Trials of Dihydroartemisinin+ Piperaquine**)**.

Having divided the two criteria of non-inferiority and comparison of study results with historical ones, power has been recomputed for each of the two objectives as described below.
For the non-inferiority analysis, the clinical and statistical specifications for the sample size computations are as follows:

- Primary endpoint = PCR-corrected cure rate at day 63.
- Primary analysis = based on 97.5% one-sided confidence intervals for the difference in cure rates.
- Alpha = 0.025 (one-sided).
- Power = 80%.
- Cure rate of Artesunate+Mefloquine in the pure ITT population = at least 92%.
- Non-inferiority margin for the difference (test-reference) = -0.05.
- Randomization: based on a 2 :1 allocation scheme (test : reference).
- Rate of patient attrition in the modified intention-to-treat population as compared to the pure ITT population (i.e. rate of non-informative withdrawals, i.e. reason number 7 of section 5) = 5%. Expected 63-day PCR corrected cure rate in this population = 93%.
- Rate of patient attrition in the Per Protocol Population as compared to the ITT population (i.e. rate of withdrawals for any reason and protocol violations) = 20%. Expected 63-day PCR corrected cure rate in this population = 95%.
- Primary Analysis Populations = both the modified Intention To Treat (ITT) and the Per Protocol Populations.

With these assumptions, the sample size of 1050 patients (700 in the Artekin group and 350 in the AS+MQ group) provides a power of approximately 80% for the lower bound of a 97.5 one-sided confidence interval for the treatment difference being above –0.05 in the modified ITT and 84% for the same analysis in the Per Protocol population (for the power calculation both continuity correction and inequality of variances under the null hypotheses have been considered).

For the historical comparisons, the clinical and statistical specifications for the sample size computations are as follows:

- Anticipated population proportion of clinical failures = as high as10%.
- Confidence level = 95% (two-sided interval).
- Precision = 5 percentage points.
- Rate of patient attrition in the Per Protocol Population as compared to the ITT population (i.e. rate of withdrawals for any reason and protocol violations) = 20%.

According to Annex 4 of the WHO document "Assessment and Monitoring of Antimalarial Drug Efficacy for the Treatment of Uncomplicated Falciparum Malaria – 2003", a sample size of at least 175 patients would be required in each treatment group for estimating the proportion of clinical failures to within a 5 percentage point of the true value with 95% confidence.

3.4.2 Sample size re-assessment

Due to the limitation of available data on the cure rate of AS+MQ in the target population and to the uncertainty regarding the expected rate of withdrawals, an interim analysis will be performed solely for sample size re-assessment. This analysis will be carried-out when approximately half of the patients originally planned (i.e. ~525 patients) are randomized and have reached the Day 63 evaluation.

This analysis will be performed for checking:

- the overall rate of patients withdrawing from the study considering all the possible reasons;
- the proportion of non evaluable PCR;
- the overall 63-day PCR corrected cure rate.

These evaluations will be carried-out in each of the two primary populations (i.e. modified ITT and PP populations) by the Data Monitoring Board. The sample size will be recomputed based on these findings and changed accordingly, if an increase is needed (i.e. no downsizing will be implemented). As a general rule, the Data Monitoring Board will base the above specified evaluations on blind data.

The sample size resulting from this assessment (either if necessitating an adjustment or not) will not be communicated to the Investigators so as not to reveal any information on the primary outcome (rate of cure) of the study.

The method for performing this analysis could be based on the papers by A.L. Gould[34] and J Herson and J. Wittes[35]. However, such methods apply to superiority studies and therefore need to be adapted for the non-inferiority case. All details of this interim analysis will be provided in the Statistical Analysis Plan (or a separate document) that will be finalized before starting any interim analysis.

MODIFICATION:

Page 28: Section 5 Patient Withdrawal Criteria

Reason for modification: Criterion number 3 specified only day 0, but actually persistent vomiting is relevant at any day.

Need to address the modified intention-to-treat population.

Therefore:

During the study, the following conditions are reasons for excluding the patients from further participation in the trial:

1. Use of antimalarial drugs outside of the study protocol.
2. Withdrawal of informed consent.
3. Lost to follow-up: patients who fail to attend a follow-up visit and are unable to be located within 48 hours on Days 1-14 or for more than 2 subsequent visits between Days 15-63.
4. Patient's request to discontinue for any reason.
5. At the investigator's request for safety reasons

is modified as follows

During the study, the following conditions are reasons for stopping the study treatment and/or for excluding the patients from further efficacy assessments.

1 Any treatment failure (including early failures).
2 Use of antimalarial drugs (or antibiotics with antimalarial activity) outside of the study protocol.
3 Persistent vomiting of study drugs (at least twice the same dose).
4 Failure to complete the study treatment.
5 Withdrawal of informed consent.
6 Investigator’s request for safety reason.
7 Lost to follow-up: patients cannot be located by Day 63.

Patients with one or more of the conditions listed above will have to be followed-up for the safety assessments until day 63. These patients will be considered failures in the intention-to-treat population, will be considered partly failures and partly excluded in the modified intention-to-treat analysis and, with exclusion of condition 1, they will be excluded from the Per Protocol population (see sections 8.1 and 8.2).

MODIFICATION:
Page 32: Section 8.1 Population Analysed
Reason for modification: There are several reasons for proposing this change:

- to consider a population, i.e. the modified intention-to-treat population that represents a reasonable compromise between the need of not distorting the effect of randomization and the need of not rendering the results too much dependent upon the rate of drop-outs;
- to specify how to treat patients with a non interpretable or missing PCR result in the ITT and modified ITT populations;
- to harmonize this study with the other phase III study of the Artekin project currently conducted in Africa.

Therefore:

The following populations will be considered for the statistical analysis of efficacy.
Intention-to-Treat (ITT) Population: Defined as all randomized patients who will take at least one dose of the study treatment. All patients who withdrew from the study for one of the reasons listed in section 5 will be evaluated as screen failures in this population.

Evaluable Population (or Per-Protocol (PP) Population): Defined as all randomized patients who will be eligible according to the study protocol, will take all the study medication, will be failures or will have the Day 63 assessment, will not take anti-malarial drugs outside of the study protocol, will have enough follow-up data (i.e. will not have violations 3 and 4 listed under section 16) and in presence of recurrent parasitaemia will have an evaluable PCR.

is modified as follows:

The following populations will be considered for the statistical analysis of efficacy.
Intention-to-Treat (ITT) Population. Defined as all randomized patients who will take at least one dose of the study treatment. All patients who withdrew from the study for one of the reasons listed in section 5 as well as the patients for whom the PCR is not interpretable or missing will be evaluated as failures. For the robustness analyses, these patients will be evaluated in different ways in this population (see section 8.2).

Modified Intention-to-Treat (modified ITT) Population. Defined as the ITT population with the exclusion of the lost-to-follow-up patients before Day 63 for unknown reasons (non informative drop-outs). More specifically, patients withdrawing from the study for reason 7 (see section 5) will be excluded, while all other withdrawals will be counted as failures (these reasons might be potentially considered correlated with lack of efficacy, i.e. are referred to as informative drop-outs). The patients for whom the PCR is not interpretable or missing will be assigned the result "recrudescence" or "re-infection" according to the ratio between these outcomes which was observed among the evaluable PCRs within each treatment group. This rule about PCR will be applied for the primary analysis. For all analyses for which the knowledge of the outcome is needed for each patient individually (for example, the Breslow Day test or the logistic regression or the analyses where a baseline covariate is needed), different scenarios will be considered , i.e. the most two extreme ones of considering all patients with not interpretable or missing PCRs as failures or as successes and the intermediate one of excluding them all.

Per-Protocol (PP) Population. Defined as all randomized patients who will be eligible according to the study protocol, will receive at least 80% of the study medication, will have the Day 63 assessment, will not take other anti-malarial drugs and, in case of the presence of asexual parasite stages on thick or thin blood smears, will have an evaluable PCR. The protocol violations listed below will also be considered reasons for excluding the patients from this population:

- failure to attend visits between Days 0 – 2 and unable to be located within 6 hours of the scheduled visits;

failure to attend two consecutive visits between Days 3 and 63 (every visit between Day 3 and Day 56 must take place within 48 hours of the scheduled time; visit at Day 63 can occur minus 1 or plus 7 days of the scheduled visit)..

MODIFICATION:
Page 32: Section 8.2 Efficacy: primary analysis
Reason for modification: There are several reasons for proposing this change:

- To specify statistical techniques that are suited for the two different objectives of proving non-inferiority on one side and comparing the study results with the historical ones on the other side (as mentioned at point 3 above, in the former case the primary focus is on bias and variability while in the latter it is on reproducibility of the experimental setting).

- To consider a method for assigning a value to the missing or non-interpretable PCR analyses in the modified intention-to-treat population.
- To provide more details on additional analyses, such as the robust analysis and the evaluation of homogeneity across centres, that are very important for the interpretation of the study results but were not provided in the original version of the protocol.
- To harmonize this study with the other phase III study of the Artekin project currently conducted in Africa.

Therefore:

The primary analysis will be based on a 97.5% (one-sided) Confidence Interval (CI) computed on the difference between the 63-day PCR corrected cure rates of the test and the reference treatments, respectively.
In order to claim that DHA+PPQ is efficacious, the lower limit of the CI must be > -0.05 and the point estimate for the 63-day PCR corrected cure rate of DHA+PPQ must be > 0.90, in both the ITT and the Evaluable populations.
The method for computing the CI will be specified in the SAP.
For computing the PCR-corrected cure rate, the following rules will be applied:

- the patients who are diagnosed to be re-infected are counted as successes (they have to be terminated on that day of follow-up);
- the patients for whom the PCR is not interpretable or is missing are counted as failures in the ITT population, while they are excluded from the Evaluable population. The proportion of these patients is less than 10% of patients who experienced a recurrent parasitaemia (i.e. those in whom the PCR evaluation is performed).

is modified as follows:

8.2.1 Analysis for showing non-inferiority of Artekin vs AS+MQ
The primary analysis will be based on a 97.5% (one-sided) Confidence Interval (CI) computed on the difference between the 63-day PCR corrected cure rates of the test and the reference treatments, respectively.
In order to claim that Artekin is non-inferior to AS+MQ the lower limit of the CI must be > -0.05 in both the modified ITT and the Per Protocol populations. The analysis on the more pure ITT population (see section 8.1) will be used for investigating robustness of results (see below).
The method for computing the CI will be specified in the SAP.
For computing the PCR-corrected cure rate, the patients who are diagnosed to be re-infected will be counted as successes (they have to be terminated on that day of follow-up) while the patients for whom the PCR is not interpretable or the sample is unavailable will be treated differently depending on the population considered (see section 8.1).
Robust analysis will be performed on the more pure ITT population by applying the following methods:
1. All patients who withdrew from the study for one of the reasons listed in section 5 (from 1 to 7) will be evaluated as failures as well as the patients for whom the PCR is not interpretable or missing
2. Patients who withdrew from the study for reasons that cannot be considered uncorrelated with the study treatments (i.e. reasons from 1 to 6) will be counted as failures (i.e. assigned with a 0 value), while the patients prematurely interrupting the study for reason

7 (lost to follow-up) will be assigned with a value ranging from 0.5 to 0.9 (imputation of a postulated probability of success for the patients for whom the treatment outcome could not be evaluated). Finally, all cured patients (or with re-infection by PCR) will be assigned with a 1 and this transformed endpoint will be summarized in terms of means and corresponding standard errors by treatment group. This analysis will be repeated for taking into account different ways of treating the patients with a non-interpretable or missing PCR (from failure to exclusion).

3. The analysis of the 63-day PCR corrected cure rates will be performed by means of the survival analysis techniques, where the non informative lost-to-follow-up patients (i.e. unable to locate the patient) and the patients with a non-interpretable or missing PCR will be censored at the time of exiting the study.

The impact of these strategies on the conclusions will be carefully evaluated and discussed.

The Breslow Day test or logistic regression will be used to evaluate homogeneity across centers. In this respect, each center would need to have at least two failures for each treatment in order for large sample chi-square approximations to be applicable. If this type of condition does not apply, exact logistic regression will be used to address homogeneity across centers. In addition, if the sample sizes are sufficiently large within centers so that each treatment has at least five failures within each center, a confidence interval adjusted for centers as strata will be computed (see Koch and others, Statistics in Medicine, 1998).

8.2.2 Historical comparisons

In order to compare the study results with the historical ones, the criteria described in the WHO document ("Assessment and Monitoring of Antimalarial Drug Efficacy for the Treatment of Uncomplicated Falciparum Malaria – 2003". See chapter 7, section 7.3) will be used. Therefore, the failure rates will be estimated through survival analysis (Kaplan-Meier method) in each treatment group individually considered. This analysis will be carried-out in the per-protocol population and the results obtained in each of the two treatment groups will be judged against the threshold of 90% (there is consensus among the malaria experts in recognising that the threshold of 90% should be considered as a "rule of thumb" for judging efficacy).

MODIFICATION:

Page 36: Section 8.4 Safety and Tolerability

Reason for modification The new definition is the standard definition of safety population (inclusion of patients not having taken any treatment would be anti-conservative).

Therefore

All randomised patients will be considered for the following analysis.

is modified as follow:

All analyses described in this section will be carried out in the Safety Population defined as all randomized patients who will receive at least one dose of the study treatment.

MODIFICATION:

Page 36: Section 15.1 Steering Committee

Reasons for modification: The composition of the Steering Committee has been changed because of the new constitution of the Clinical Development Committee

Therefore:

The Steering Committee (SC) comprises at least one investigator from each participating region and will assess the progress of the trial. The members of the SC will address policy and operational issues related to the protocol. The SC has responsibility for protecting the scientific conduct and integrity of the trial. Its functions include:
Review of the protocol before ethic committee approval,
Formulation of recommendation for any change in the design and operations of the trial during the course of the trial, when needed,
Exclusion of patients from the per protocol analysis.
The members of the Steering Committee are:
- Professor Umberto D'Alessandro (Prince Leopold Institut of Tropical Medicine,Nationalestraat 155, B-2000 Antwerp-Belgium).
- Dr John Solomon (MDS Pharma Services).
- Professor Nicholas J White (Wellcome Trust Southeast Asian Tropical Medicine Research Units, Faculty of Tropical Medicine Mahidol University, 420/6 Rajvithi Rd, Phayathai 10400, Bangkok, THAILAND).
- Dr. Antonella Bacchieri (Sigma-Tau - Medical Department, Head of Biostatistics and Data Management).

is modified as follow:

The Steering Committee (SC) comprises at least one investigator from each participating region and will assess the progress of the trial. The members of the SC will address policy and operational issues related to the protocol. The SC has responsibility for protecting the scientific conduct and integrity of the trial. Its functions include:

- Review of the protocol before ethic committee approval,
- Formulation of recommendation for any change in the design and operations of the trial during the course of the trial, when needed,
- Exclusion of patients from the per protocol analysis.

The members of the Steering Committee will be specified in the Terms of Agreement document that will be prepared no later than the first face-to-face meeting of this Committee.

MODIFICATION:

Page 22: Section 15.3 Clinical Development Committee

Reason for modification: Need to consider a Committee responsible for transforming the recommendations from both the Data Monitoring Committee and the study Steering Committee into final decisions, safeguarding at the same time the harmonization among the studies of the Artekin project.

Therefore:

The following is added:

The Clinical Development Committee will be responsible for:

- evaluating the recommendations of both the Data Monitoring Committee and the study Steering Committee and, if deemed appropriate, transforming them in operational decisions;
- protecting the scientific conduct, the ethical integrity, and the regulatory acceptability of the Artekin project;
- harmonizing all the studies of the Artekin project;
- addressing policies and operational issues that can impact the Artekin project.

The members appointed to this Committee will be specified in the Terms of Agreement document that will be prepared no later than the first face-to-face meeting of this Committee.
