# Supplementary material for: An Open-Label, Randomised Study of Dihydroartemisinin-Piperaquine Versus Artesunate-Mefloquine for Falciparum Malaria in Asia
Source: PLoS One. 2010 Jul 30;5(7):e11880. doi: 10.1371/journal.pone.0011880 (PMC2912766; doi:10.1371/journal.pone.0011880)
Supplement: Protocol S5 — Protocol amendment 3A. (0.08 MB PDF) [file pone.0011880.s006.pdf]

**Amendment N°:**	**3**
**Protocol N°:**	**ST3073-ST3074 DM040010**

Title:

A Phase III, randomized, non-inferiority trial, to assess the efficacy and safety of Dihydroartemisinin+Piperaquine (DHA+PPQ, Artekin) in comparison with Artesunate+Mefloquine (AS+MQ) in patients affected by acute, uncomplicated *Plasmodium falciparum* malaria.

- MULTICENTRE STUDY IN ASIA-

**Final protocol dated :**	**10 January, 2005**
**Revised protocol incorporating Amendment N° 1, 2& 3 dated:**	**24 November 205**
**Amendment N° 3A (SMRU sites only)dated:**	**6 January 2006**

PURPOSE:

.

This amendment has been formulated in response to specific recommendations from the Thai MoPH.

MODIFICATION:
Page 5: Synopsis Diagnosis and Main Inclusion Criteria
Reason for modification: To amend the age range to reflect that only adults will be recruited

Therefore:

Males and Females aged between 3 months and 65 years inclusive, body weight at least 5 Kg, microscopically confirmed, monoinfection of *Plasmodium falciparum* or mixed infection, history of fever or presence of fever (temperature at ≥ 37.5 °C), written informed consent

is modified as follows :

Males and Females ≥18 years with microscopically confirmed, monoinfection of *Plasmodium falciparum* or mixed infection, history of fever or presence of fever (temperature at ≥ 37.5°C), written informed consent
.

MODIFICATION:
Page 22: Section 3. Selection of the Patients
Inclusion Criteria Number 1
Reason for modification: To amend the age range to reflect that only adults will be recruited.

Therefore:

Males and Females aged between 3 months and 65 years inclusive.

is modified as follows :

Males and Females aged ≥ 18 years

.
