# Supplementary material for: An Open-Label, Randomised Study of Dihydroartemisinin-Piperaquine Versus Artesunate-Mefloquine for Falciparum Malaria in Asia
Source: PLoS One. 2010 Jul 30;5(7):e11880. doi: 10.1371/journal.pone.0011880 (PMC2912766; doi:10.1371/journal.pone.0011880)
Supplement: Protocol S6 — Protocol amendment 3B. (0.09 MB PDF) [file pone.0011880.s007.pdf]

**Amendment N°:**	**3B**
**Protocol N°:**	**ST3073-ST3074 DM040010**

Title:

A Phase III, randomized, non-inferiority trial, to assess the efficacy and safety of Dihydroartemisinin+Piperaquine (DHA+PPQ, Artekin) in comparison with Artesunate+Mefloquine (AS+MQ) in patients affected by acute, uncomplicated *Plasmodium falciparum* malaria.

- MULTICENTRE STUDY IN ASIA-

**Final protocol dated:**	**10 January 2005**
**Revised protocol incorporating Amendment N° 1 & 2 dated:**	**14 April 2005**
**Revised protocol incorporating Amendment N° 1, 2 & 3 dated:**	**24 November 2005**
**Amendment N° 3B (India sites only) dated:**	**21 April 2006**

PURPOSE:

.
The main goal of this amendment is to accommodate local requirements of the sites in India.

<u>MODIFICATION:</u>
Page 5: Synopsis: Diagnosis and Main Inclusion Criteria
Reason for modification: To amend the lower age limit to reflect that only adults will be recruited.

Therefore:

Males and Females aged between 3 months and 65 years inclusive, body weight at least 5 Kg, microscopically confirmed, monoinfection of *Plasmodium falciparum* or mixed infection, history of fever or presence of fever (temperature at ≥ 37.5 °C), written informed consent

is modified as follows :

Males and Females aged ≥18 years with microscopically confirmed, monoinfection of *Plasmodium falciparum* or mixed infection, history of fever or presence of fever (temperature at ≥ 37.5°C), written informed consent
.

<u>MODIFICATION:</u>
Page 24: Section 3.7. Selection of the Patients
Inclusion Criterion Number 1
Reason for modification: To amend the lower age limit to reflect that only adults will be recruited.

Therefore:

Males and Females aged between 3 months and 65 years inclusive.

is modified as follows :

Males and Females aged ≥18 years.

<u>MODIFICATION:</u>
Page 30: Section 4.1. Concomitant Drug Therapies Disallowed
Reason for modification: As chloroquine may still be effective on *Plasmodium falciparum* in some areas in India, the use of chloroquine during the study would be prohibited.

Therefore:

Any antimalarial, or antibiotic with antimalarial activity (erythromycin or other macrolides, co-trimoxazole or other sulfonamides, any tetracycline including doxycycline, and quinolones). The exception to this is chloroquine which may be prescribed for non-falciparum infections during follow up.

is modified as follows :

Any antimalarial (including chloroquine), or antibiotic with antimalarial activity (erythromycin or other macrolides, co-trimoxazole or other sulfonamides, any tetracycline including doxycycline, and quinolones).

MODIFICATION:

Page 30: Section 4.2. Concomitant Drug Therapies Allowed

Reason for modification: As chloroquine may still be effective on *Plasmodium falciparum* in some areas in India, it would not be categorised as one of the drug treatments allowed.

Therefore:

During the trial patients can receive certain prescribed drugs e.g. paracetamol, and non malarial antibiotics (penicillins, cephalosporins), chloroquine.

.

is modified as follows :

During the trial patients can receive certain prescribed drugs e.g. paracetamol, and non malarial antibiotics (penicillins, cephalosporins).
