# Supplementary material for: An Open-Label, Randomised Study of Dihydroartemisinin-Piperaquine Versus Artesunate-Mefloquine for Falciparum Malaria in Asia
Source: PLoS One. 2010 Jul 30;5(7):e11880. doi: 10.1371/journal.pone.0011880 (PMC2912766; doi:10.1371/journal.pone.0011880)
Supplement: Protocol S7 — Protocol amendment 4. (0.09 MB PDF) [file pone.0011880.s008.pdf]

**Amendment N°:**	**4**
**Protocol N°:**	**ST3073-ST3074 DM040010**

Title:

A Phase III, randomized, non-inferiority trial, to assess the efficacy and safety of Dihydroartemisinin+Piperaquine (DHA+PPQ, Artekin) in comparison with Artesunate+Mefloquine (AS+MQ) in patients affected by acute, uncomplicated *Plasmodium falciparum* malaria.

- MULTICENTRE STUDY IN ASIA-

**Final protocol dated:**	**10 January 2005**
**Revised protocol incorporating Amendment N° 1 & 2 dated:**	**14 April 2005**
**Revised protocol incorporating Amendment N° 1, 2 & 3 dated:**	**24 November 2005**
**Amendment N° 3B (Indian Sites only) dated**	**21 April 2006**
**Amendment N° 4 dated**	**13 Sept 2006**

PURPOSE:

The main goal of this amendment is to include in the statistical section a test for the cohort effect.

MODIFICATION:

Page 32 - Section 8.2 Efficacy: primary analysis

Reason for modification: Due to delays in obtaining the regulatory authorization for some of the Thai centres, the study enrolled only almost half of the planned sample size in the malaria season of 2005. The sites that received the regulatory authorization only in 2006 will complete the study in the malaria season of this year. Therefore, patient enrolment will be split into two different malaria seasons with different sites working in the two periods.

Therefore the following text must be added in the statistical section after "The method for computing the CI will be specified in the SAP" and before "All patients who withdrew from …"

Due to delays in obtaining the regulatory authorization for some of the participating centres, patient enrolment will be split into two malaria seasons, with different sites working in the two study periods. In order to evaluate how the 63-day PCR corrected cure rates vary across these two cohorts, a logistic regression model will be fit with cohort and treatment as the explanatory variables. The treatment by cohort interaction will be evaluated as a candidate to enter this model through a residual score test at the 0.10 significance level. If either the treatment by cohort interaction by the score test or the cohort effect in the logistic regression model have $p<0.10$, the CI for the primary analysis will be stratified. Also, if the test for treatment by cohort interaction has $p< 0.10$, separate CI's will be provided for the two cohorts.
