# Supplementary material for: An Open-Label, Randomised Study of Dihydroartemisinin-Piperaquine Versus Artesunate-Mefloquine for Falciparum Malaria in Asia
Source: PLoS One. 2010 Jul 30;5(7):e11880. doi: 10.1371/journal.pone.0011880 (PMC2912766; doi:10.1371/journal.pone.0011880)
Supplement: Protocol S8 — Protocol amendment 5. (0.10 MB PDF) [file pone.0011880.s009.pdf]

σ sigma-tau
Industrie Farmaceutiche Riunite S.p.A.
Sede Legale: Roma, V.le Shakespeare 47
Cap. Sociale: 30.500.000.000 inter. versato
Cod. Fiscale: 00410650584
P. IVA n°: 00885531004
Canc. Comm. Trib. di Roma n° 1468/57
Iscriz. Sch. Naz. Ric. Cod. G0120YKG
c.c.i.a.a.: Roma n° 205785

MEDICAL DEPARTMENT - *Research & Development Department*

Artekin

Study Protocol No.: ST3073-ST3074 DM040010

(Revised Final Protocol v3.00 incorporating Amendments N° 1,2, 3, 4 &5 dated 27th Dec 2006)

A Phase III, randomized, non-inferiority trial, to assess the efficacy and safety of Dihydroartemisinin+Piperaquine (DHA+PPQ, Artekin) in comparison with Artesunate+Mefloquine (AS+MQ) in patients affected by acute, uncomplicated *Plasmodium falciparum* malaria.

- MULTICENTRE STUDY IN ASIA-

Co-ordinating Investigator
Professor Nicholas J White
Wellcome Trust Southeast Asian Tropical Medicine Research Units
Faculty of Tropical Medicine Mahidol University
420/6 Rajvithi Rd, Phayathai 10400
Bangkok, THAILAND

**Approval**	**Signature**	**Date**
Prof./Dr.	Investigator	____/____/2006
Prof. Nick White	Co-ordinating Investigator	____/____/2006 Resigned 8/5/07
Dr. Marco Corsi	Sigma-Tau - Medical Department<br>Director	____/____/2006
Dr. Antonella Bacchieri	Sigma-Tau - Medical Department<br>Head of Biostatistics and Data Management	____/____/2006

Signing this document I declare to have read the paragraph relevant to study acknowledgement and confidentiality and authorise Sigma-Tau i.f.r. S.p.A., Pomezia (Rome) - Italy to record my data on a computerised archive containing all the data pertinent to the study.

**Approval**	**Signature**	**Date**
Prof./Dr. ……………..…….	Investigator	____/____/2006
Prof. Nick White	Co-ordinating Investigator	____/____/2006
Dr. Marco Corsi	[signature] Sigma-Tau - Medical Department Director	27/12/2006
Dr. Antonella Bacchieri	[signature] Sigma-Tau - Medical Department Head of Biostatistics and Data Management	27/12/2006

Signing this document I declare to have read the paragraph relevant to study acknowledgement and confidentiality and authorise Sigma-Tau i.f.r. S.p.A., Pomezia (Rome) - Italy to record my data on a computerised archive containing all the data pertinent to the study.

Amendment N°: 5

Protocol N°: **ST3073-ST3074 DM040010**

Title:
A Phase III, randomized, non-inferiority trial, to assess the efficacy and safety of Dihydroartemisinin+Piperaquine (DHA+PPQ, Artekin) in comparison with Artesunate+Mefloquine (AS+MQ) in patients affected by acute, uncomplicated Plasmodium falciparum malaria.

-MULTICENTRE STUDY IN ASIA -

**Final protocol dated:**	**10 January 2005**
**Revised protocol incorporating Amendment N° 1 & 2 dated:**	**14 April 2005**
**Revised protocol incorporating Amendment N° 1, 2 & 3 dated:**	**24 November 2005**
**Amendment N° 3B (Indian Sites only) dated**	**21 April 2006**
**Amendment N° 4 (Indian Sites only) dated**	**13 September 2006**
**Amendment N° 5 dated**	**27 December 2006**

PURPOSE:

The main goal of this amendment is that the number of patients to be recruited in India to be increased to 150 which would exceed the total sample size of 1050. This is to ensure the minimum representative sample size from the country.

MODIFICATION:

Page 5 - Synopsis
Reason for modification: In order to ensure that there is sufficient representative sample size from India, the total number of patients to be recruited by the Indian sites is 150.

Therefore:

Number of Subjects	1050 patients (700 DHA+PPQ; 350 AS+MQ).

is modified as follows :

Number of Subjects	1150 patients (767 DHA+PPQ; 383 AS+MQ).

Page 17 - Section 3.1 Study Design
Reason for modification: In order to ensure that there is sufficient representative sample size from India, the total number of patients to be recruited by the Indian sites is 150.

Therefore:
This is a phase III, randomized, open label, two arms study.
Investigational centres will be involved in the enrolment of 1050 patients (700 DHA+PPQ; 350 AS+MQ).

is modified as follows :
This is a phase III, randomized, open label, two arms study.
Investigational centres will be involved in the enrolment of 1150 patients (767DHA+PPQ; 383 AS+MQ).

Page 19 - Section 3.4 Sample Size
Reason for modification: In order to ensure that there is sufficient representative sample size from India, the total number of patients to be recruited by the Indian sites is 150.

Therefore:
With these assumptions, the sample size of 1050 patients (700 in the Artekin group and 350 in the AS+MQ group) provides a power of approximately 80% for the lower bound of a 97.5 one-sided confidence interval for the treatment difference being above –0.05 in the modified ITT and 84% for the same analysis in the Per Protocol population (for the power calculation both continuity correction and inequality of variances under the null hypotheses have been considered).

is modified as follows :

With these assumptions, the sample size of 1150 patients (767 in the Artekin group and 383 in the AS+MQ group) provides a power of approximately 80% for the lower bound of a 97.5 one-sided confidence interval for the treatment difference being above –0.05 in the modified ITT and 84% for the same analysis in the Per Protocol population (for the power calculation both continuity correction and inequality of variances under the null hypotheses have been considered).

Investigational centres will be involved in the enrolment of 1150 patients (767 DHA+PPQ; 383 AS+MQ).
